# UBE2M-mediated neddylation modification stabilizes VEGFR2 to delay pulmonary vascular endothelial cell senescence

**DOI:** 10.1038/s41419-026-08881-0

**Published:** 2026-05-28

**Authors:** Yunhao Chang, Qiao Chen, Xia Xu, Xinxing Lyu, Linlin Xu, Qingxia Hu, Yu Han, Yutao Wu, Xiaomeng Zhang, Li Qiao, Zhihao Cai, Shuhong Huang, Jianqing Wu, Bo Chen

**Affiliations:** 1 Department of Geriatrics, Jiangsu Province Hospital, The First Affiliated Hospital with Nanjing Medical University, Nanjing, China; 2 Geriatric Medicine Center, Department of Geriatric Medicine, Zhejiang Provincial People’s Hospital, People’s Hospital of Hangzhou Medical College, Hangzhou, Zhejiang China; 3 Department of Geriatrics & Key Laboratory of Cardiovascular Proteomics of Shandong Province, Qilu Hospital, Cheeloo College of Medicine, Shandong University, Jinan, China; 4 Hospital for Skin Diseases, Shandong First Medical University, Jinan, China; 5 Department of Dermatology, Shandong Provincial Hospital, Shandong University, Jinan, China; 6 School of Clinical and Basic Medicine, Shandong First Medical University & Shandong Academy of Medical Sciences, Jinan, China

**Keywords:** Senescence, Ubiquitins, Respiratory tract diseases

## Abstract

Pulmonary aging is characterized by progressive structural and functional decline. Neddylation is recognized as a crucial mechanism for maintaining cellular homeostasis; however, its function in pulmonary aging has not been fully elucidated. In this study, we found that the core neddylation E2 enzyme UBE2M was downregulated in aged lung tissues. *Ube2m* knockdown mice exhibited premature pulmonary aging, including vascular degeneration and structural disruption. Notably, in the lungs of knockout mice, although VEGF expression—primarily secreted by epithelial cells—remained unchanged, the protein level and phosphorylation of its receptor VEGFR2 on endothelial cells were significantly reduced. Mechanistic investigation confirmed that UBE2M directly regulates VEGFR2 stability in pulmonary endothelial cells via neddylation. In a doxorubicin-induced endothelial cell senescence model, UBE2M downregulation was accompanied by impaired VEGFR2 signaling, whereas UBE2M reconstitution partially alleviated cellular senescence. In elastase-induced emphysema mouse models and in lung tissues from COPD patients, both UBE2M and VEGFR2 levels were markedly reduced, and Ube2m deficiency exacerbated lung injury. In conclusion, this study demonstrates that UBE2M delays pulmonary aging by stabilizing VEGFR2 through neddylation, suggesting its potential as a therapeutic target for age-related pulmonary diseases.

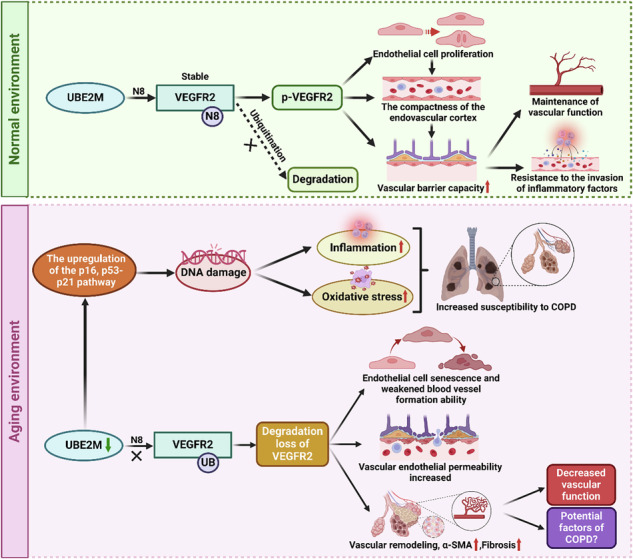

## Introduction

A gradual decline in vascular function is considered a key factor driving organ aging [1]. This degenerative change affects not only the stability of the circulatory system but also the health of multiple organs [1, 2]. Vascular aging is characterized by a series of interconnected pathophysiological processes, including oxidative stress, chronic low-grade inflammation, and endothelial cell dysfunction. These processes lead to vascular wall stiffening, thickening, and loss of elasticity, ultimately contributing to vascular stiffness and reduced blood flow [2, 3]. Moreover, endothelial dysfunction impairs the ability of blood vessels to dilate in response to stimuli, thereby exacerbating the decline in circulatory efficiency [3]. These vascular changes are closely linked to various age-related diseases such as hypertension, atherosclerosis, and chronic obstructive pulmonary disease (COPD), which are among the leading causes of morbidity and mortality in the older population [3, 4].

In the lungs, the decline in vascular function is particularly critical, as it compromises the integrity of the pulmonary gas-blood barrier—the structural foundation for efficient gas exchange and hemodynamic stability [4]. The pulmonary vasculature plays a pivotal role in supporting the exchange of oxygen and carbon dioxide between the blood and alveoli. As vascular function declines with age, it can lead to pulmonary hypertension and impaired lung function, contributing to the onset of chronic respiratory diseases, such as emphysema and pulmonary fibrosis [5, 6].

Endothelial cells form a single layer that lines the inner surface of blood vessels, regulates vascular permeability, maintains hemodynamic balance, and participates in inflammatory responses [7–9]. These cells also produce signaling molecules such as nitric oxide (NO), prostacyclin (PGI₂), and endothelin-1 (ET-1) that modulate vascular tone and integrity [9]. Recent studies have shown that restoring pulmonary endothelial cell function can significantly relieve conditions such as emphysema, highlighting the crucial role of endothelial cells in maintaining pulmonary vascular homeostasis [10].

Among the numerous signaling pathways involved in maintaining the pulmonary gas-blood barrier, the VEGFA-VEGFR2 axis holds a central position. This barrier is composed of vascular endothelial and alveolar epithelial cells, where VEGF, secreted by epithelial cells, specifically acts on VEGFR2, located on endothelial cells, thereby precisely regulating angiogenesis and barrier repair [11]. VEGFR2 is a receptor tyrosine kinase that, upon binding to its ligand, VEGF, activates downstream signaling pathways crucial for regulating endothelial function, including cell proliferation, migration, and survival. Its activity is closely associated with endothelial health, and disruption of the VEGF–VEGFR2 signaling pathway is known to impair endothelial cell function [11]. Recent research has shown that activating the VEGF–VEGFR2 pathway can significantly improve organ function, alleviate aging-related pathology, and even extend lifespan [12].

Neddylation is a post-translational modification that has emerged as a significant regulator of protein stability, activity, and localization. This process involves the activation of neural precursor cell-expressed developmentally downregulated 8 (NEDD8), a small ubiquitin-like protein that is covalently attached to target proteins to regulate their function [13, 14]. Neddylation plays a critical role in various cellular processes, including protein degradation, cell cycle regulation, and the DNA damage response. Ubiquitin-conjugating enzyme E2M (UBE2M) is a key E2 enzyme in the neddylation process and is responsible for transferring NEDD8 to target proteins. By modulating the stability and activity of target proteins such as Cullins, UBE2M is involved in regulating cellular pathways related to cancer, chronic inflammation, and metabolic diseases [14–19]. However, its specific roles in aging and pulmonary vascular health remain largely unknown.

This study reveals that UBE2M is significantly downregulated in naturally aged mice and COPD models and that UBE2M plays a key role in pulmonary vascular endothelial cell aging by regulating VEGFR2 stability through neddylation. Further experiments show that the knockdown of *Ube2m* accelerates the occurrence of emphysema and vascular stiffening during COPD progression, whereas its overexpression demonstrates anti-aging potential. These results provide new insights into the pivotal role of UBE2M in the regulation of pulmonary vascular aging, laying the foundation for its potential use as a therapeutic target in chronic lung diseases.

## Materials and Methods

### Patients

The study was conducted in accordance with the principles of the Helsinki Declaration and was approved by the Ethics Committee of Qilu Hospital of Shandong University (Approval No. KYLL-202204-035). Written informed consent was obtained from all participants prior to their inclusion. Lung tissue specimens were collected from individuals undergoing thoracoscopic surgery or pulmonary resection at Qilu Hospital of Shandong University. Participants were stratified into two groups based on clinical diagnosis: the COPD group and the non-COPD group. All tissue specimens were obtained from tumor-adjacent normal lung tissues. Following resection, tissues were fixed in 4% neutral buffered formalin for histopathological evaluation. Furthermore, lung tissue samples underwent HE staining, Masson’s trichrome staining, Sirius Red staining, and immunofluorescence staining to assess histological alterations, extracellular matrix remodeling, collagen deposition, and specific protein expression patterns.

### Mice studies

All animal experiments were reviewed and approved by the Institutional Animal Care and Use Committee of Shandong First Medical University (Approval No. W202406130635) and conducted in adherence to the NIH guidelines for the care and use of laboratory animals. Both male and female mice were examined in this study, and similar findings were reported for both sexes, with the exact numbers per sex specified in the figure legends.

UBE2M-deficient mice were generated by Gempharmatech Co., Ltd (Nanjing, China; Strain ID: T003161) using the CRISPR/Cas9 gene-editing system. Based on the gene structure, exons 2 to 4 of the Ube2m-201 transcript (ENSMUST00000005714.13) were selected as the knockout region, which contains 238 bp of coding sequence. Disruption of this region was designed to result in loss of protein function. CRISPR/Cas9 system components were microinjected into fertilized eggs of C57BL/6JGpt mice, and the injected eggs were transplanted into pseudopregnant females to obtain F0 founder mice. Positive F0 mice were confirmed by PCR and sequencing, and a stable F1 generation was obtained by mating positive F0 mice with wild-type C57BL/6JGpt mice. Consistent with existing MGI data, mice homozygous for this knockout allele exhibited early embryonic lethality; therefore, all experiments were performed using heterozygous (Ube2m⁺/⁻) mice.

Genotyping was performed by polymerase chain reaction (PCR) using primers Ube2m-tF1 (5’-CCGTGTCGTGAAGATTGTGAAGG-3’) and Ube2m-tR3 (5’-AGGCTGCTACAGGCCCTGCTAATAC-3’). This primer set produced a 1435 bp product for the wild-type allele and a 288 bp product for the knockout allele. Heterozygous mice were identified by the presence of both the 1435 bp and 288 bp bands. The absence of UBE2M protein expression in heterozygous mice was further confirmed by Western blot analysis.

C57BL/6 J wild-type mice were obtained from the Animal Center of Shandong First Medical University. All mice were housed under standard environmental conditions (25 ± 2 °C, 12-hour light/dark cycle) with free access to standard food and water. Age-matched wild-type mice were used as controls: 8-week-old mice for the COPD experiments and 3-month-old mice for all other experiments. No animals or samples were excluded from the analysis. All data points were included. Mice were randomly assigned to experimental groups using a computer-generated random number sequence. Blinding was not performed due to the nature of the study. This study was conducted in accordance with the ARRIVE guidelines (PLoS Biol. 8(6), e1000412, 2010).

### Cell culture

HUVECs were purchased from OriCell (cat# HUVEC-20001) and cultured in HUVEC complete medium (cat# HUVEC-90011) following the manufacturer’s instructions. HEK 293 T cells and mouse lung vascular endothelial cells were obtained from ATCC and cultured in DMEM complete medium containing 10% fetal bovine serum, 1% penicillin-streptomycin, and 1% glutamine. Cells were maintained at 37 °C in a humidified incubator with 5% CO₂. The medium was replaced every 2–3 days, and cells were passaged when they reached 80–90% confluence. All cell lines were routinely tested for mycoplasma contamination using a PCR-based method and were negative.

### Antibodies and reagents

VEGFR2 (cat# 26415­1­AP), α­SMA (cat# 67735­1­Ig), CD31 (cat# 66065­2­Ig), p21 (cat# 10355­1­AP), p16 (cat# 10883­1­AP), IgG (cat# 30000­0­AP), NF­κB (p65) (cat# 10745­1­AP), caspase 3 (cat# 9677­1­AP), p53 (cat# 10442­1­AP), GAPDH (cat# 50004­1­Ig), UBC12 (UBE2M) (cat# 14520­1­AP), FLAG (cat# 20543­1­AP), NEDD8 (cat# 16777­1­AP), Vimentin (cat# 10366­1­AP), Occludin (cat# 27260­1­AP), Claudin 5 (cat# 29767­1­AP), TGF­beta 1 (cat# 21898­1­AP), p38 MAPK (cat# 14064-1-AP), mTOR (cat# 28273-1-AP), p-p38 MAPK (Thr180/Tyr182) (CAT#28796-1-AP), Beta Catenin (cat# #51067-2-AP), Cullin1(cat# 12895-1-AP), RBX1(cat# 14895-1-AP), STAT3 (cat# 10253-2-AP) and MYC tag (cat# 16286­1­AP) antibodies were purchased from Proteintech. VE cadherin (cat# A12416), E cadherin (cat# A3149), VEGFA (cat# A12303), and IL­6 (cat# A11115) antibodies were purchased from Abclonal. phospho­VEGFR2 (Tyr1175) (cat# #3770) was purchased from Cell Signaling Technology. p-STAT3(S727) (cat# ab32143) and p-mTOR(S2448) (cat# ab109268) were purchased from abcam. HRP­labeled goat anti­mouse IgG (cat# b 10003) and goat anti­rabbit IgG (cat# b10002) were purchased from DiagBio. CoraLite594­conjugated goat anti­mouse IgG (SA00013­3), CoraLite594­conjugated goat anti­rabbit IgG (SA00013­4), 488­goat anti­rabbit recombinant secondary antibody (RGAR002), and CoraLite488­conjugated goat anti­mouse IgG were purchased from Proteintech. MLN4924 (cat# HY­10484) and doxorubicin hydrochloride (cat# HY­15142) were purchased from MedChemExpress. Puromycin (solution 10 mg/mL) was purchased from YEASEN.

### Plasmid and siRNA transfection

The Flag-VEGFR2, MYC-NEDD8, UBE2M, and UBE2MC111S plasmids were purchased from Youbio. siRNAs targeting UBE2M (5′-CAGAGGUCCUGCAGAACAA-3′), CUL1 (5′-GGTCGCTTCATAAACAACA-3′), CUL2 (5′-GCCCUUACGUCAGUUGUAAAUUACA-3′), CUL3 (5′-GAAGGAATGTTTAGGGATA-3′), CUL4A (5′-GAAGCUGGUCAUCAAGAAC-3′), CUL4B (5′-AAGCCUAAAUUACCAGAAA-3′), and CUL5 (5′-GTCTCACTTCCTACTGAACTG-3′) were purchased from Research Cloud Biology. Lipofectamine 2000 (cat# 11668) and Lipofectamine RNAiMAX (cat # 13778) were purchased from Invitrogen. The transfection experiments followed the manufacturer’s instructions. The control group cells were transfected with related empty vector plasmids or negative control siRNA.

### Western blot

Proteins were extracted from cells or tissues using RIPA buffer (cat# CW2333S, CWBIO) supplemented with protease inhibitor (cat# HY­K011, MedChemExpress) and phosphatase inhibitor (cat# CW2383S, CWBIO). The protein concentration was determined using the BCA Protein Assay Kit (cat# CW00145, CWBIO) according to the manufacturer’s instructions. The proteins were mixed with 4× sodium dodecyl sulfate (SDS) loading buffer (cat# P1016, Solarbio) and heated at 95 °C for 5 min.

The protein samples were loaded onto 15% or 6% SDS­polyacrylamide gels for electrophoretic separation, transferred to polyvinylidene difluoride membranes (cat# ISEQ00010, Millipore), and blocked with 5% skimmed milk powder dissolved in Tris­buffered saline with Tween (TBS­T) for 1 h at 25 ± 2 °C temperature. The membrane was incubated with the primary antibodies in TBS­T overnight at 4 °C. After washing with TBS­T (3 × 10 min), the membrane was incubated with secondary antibody for 1 h at 25 ± 2 °C. Protein bands were visualized using a chemiluminescence kit (cat# PK10002, Proteintech) and imaged using a chemiluminescent imaging system (4800CE, Tanon). Densitometric analysis was performed using ImageJ.

### Co­immunoprecipitation

Cells were harvested and lysed in ice­cold lysis buffer (50 mM Tris­HCl, pH 7.4; 50 mM NaCl; 0.2% NP­40; and 2 mM DTT) supplemented with protease inhibitor cocktail (cat# HY­K011, MedChemExpress) and phosphatase inhibitor cocktail (cat# CW2383S, CWBIO). The lysates were centrifuged at 41,720 ×*g* for 15 min at 4 °C to remove debris, and the supernatants were collected.

Protein A/G magnetic beads (cat# L­1204, BioLinkedin) were pre­incubated with the indicated primary antibody for 2 h at 4 °C with gentle rotation to allow antibody conjugation. After washing the beads to remove unbound antibodies, the prepared antibody–bead complexes were added to the protein lysates and incubated overnight at 4 °C with gentle rotation.

The beads were washed thoroughly three times with ice­cold lysis buffer to remove non­specific interactions. The bound proteins were eluted by heating the beads in 4× SDS loading buffer at 95 °C for 5 min. The eluted samples were then separated by SDS-polyacrylamide gel electrophoresis and analyzed by standard Western blot using specific primary and secondary antibodies. Cell lines were authenticated by the suppliers.

### Quantitative real­time PCR

Total RNA was isolated from cells using TRIzol reagent (cat# CW0850S, CWBIO) according to the manufacturer’s protocol. The extracted RNA was reverse­transcribed into cDNA using all­in­one RT SuperMix for quantitative real­time PCR (qPCR) (cat# AG0305, SparkJade) with random primers in a total reaction volume of 20 μL. Quantitative real­time PCR (RT-qPCR) was performed using SYBR Green qPCR Mix (cat# AH0104, SparkJade) on a LightCycler 96 Roche system. The RT-qPCR primer sequences with corresponding product sizes (determined via NCBI Primer-BLAST analysis) are provided in Table S1. All primers were validated for specificity against the reference transcriptome using NCBI Primer-BLAST. Amplification was performed at a uniform annealing temperature of 60 °C. Relative gene expression levels were normalized to β­actin. All experiments were performed in biological triplicates, with technical duplicates for each reaction.

### Hematoxylin and eosin (HE) staining

Hematoxylin and eosin (HE) staining was performed on tissue sections. Paraffin-embedded sections were dewaxed, rehydrated, and stained with hematoxylin for 3 min followed by eosin for 15 seconds. After dehydration and clearing, sections were mounted with neutral balsam. Images were acquired using a Nikon A1R HD25 confocal microscope.

### Masson’s trichrome staining

Masson’s trichrome staining was performed using a commercial kit (Servicebio, cat# G1006) according to the manufacturer’s instructions. Paraffin-embedded sections were dewaxed, rehydrated, and incubated in Masson solution A at 65 °C for 30 min. After washing, sections were stained with a mixture of Masson solutions B and C for 1 min, followed by differentiation, then incubated sequentially in Masson solution D for 6 min, Masson solution E for 1 min, and Masson solution F for 2-30 s. Sections were rinsed with 1% acetic acid, dehydrated through graded ethanol, cleared in xylene, and mounted with neutral balsam. Images were acquired using a Nikon A1R HD25 confocal microscope.

### Picro-Sirius red staining

Picro-Sirius red staining was performed using a modified staining kit (Servicebio, cat# G1078) according to the manufacturer’s instructions. For paraffin-embedded sections, samples were dewaxed, rehydrated, and then incubated in Sirius Red A solution at 65 °C for 30 min, followed by staining with modified Sirius Red B solution for 2 min and modified Sirius Red C solution for 1 h. Frozen sections were thawed, fixed with tissue fixative for 15 min, and then processed similarly. After rapid dehydration in three changes of anhydrous ethanol and clearing in xylene, sections were mounted with neutral balsam. Images were acquired using a Nikon A1R HD25 confocal microscope.

### FITC-Dextran permeability assay

Endothelial permeability was assessed using FITC-labeled Dextran (Beyotime, cat# ST2930-50mg, molecular weight 4000) according to the manufacturer’s instructions. FITC-Dextran was added to HUVEC culture medium at a final concentration of 1 mg/mL and incubated for 2 h at 37 °C. After incubation, cells were washed and imaged using a fluorescence microscope (Nikon A1R HD25). Permeability was quantified by measuring fluorescence intensity using ImageJ and normalized to the control group.

### Immunofluorescence staining of tissue sections

For immunofluorescence analysis, two detection methods were employed: conventional indirect immunofluorescence and multiplex tyramide signal amplification (TSA). Tissue sections underwent deparaffinization, rehydration, heat-induced antigen retrieval in citrate buffer, and peroxidase quenching, while cultured cells were fixed with 4% paraformaldehyde and permeabilized with 0.3% Triton X-100. After blocking with 10% normal goat serum (CWBIO, cat# CW0130) and overnight incubation with primary antibodies at 4 °C, samples were processed according to the respective detection protocol: conventional staining involved incubation with fluorophore-conjugated secondary antibodies (Proteintech) for 1 h at room temperature, whereas multiplex TSA involved incubation with HRP-conjugated secondary antibodies followed by tyramide labeling using fluorophore-conjugated tyramide reagents (ABclonal TSA Kit, cat# RK05904P) for 5–10 min in the dark, with HRP inactivation between sequential rounds. All samples were counterstained with DAPI (Abcam, cat# ab104139), mounted with anti-fade medium, and imaged on Olympus FV1200 IXCOV and Nikon A1R HD25 confocal microscopes. Primary antibody omission served as a negative control for all experiments.

### Immunofluorescence staining of HUVECs

For immunofluorescence staining, HUVECs cultured on coverslips were washed with PBS, fixed with 4% paraformaldehyde for 20 min, and permeabilized with 0.3% Triton X-100 for 15 min at room temperature. After blocking with 10% normal goat serum (CWBIO, cat# CW0130) for 1 h, cells were incubated with primary antibodies overnight at 4 °C, followed by incubation with fluorophore-conjugated secondary antibodies for 1 h at room temperature. Coverslips were then counterstained with DAPI (Abcam, cat# ab104139) and mounted with anti-fade mounting medium. Fluorescence images were acquired using a Nikon A1R HD25 confocal microscope. Negative controls omitting primary antibodies were included to confirm specificity.

### Transmission electron microscopy

Pulmonary tissues were dissected into 1–2 mm³ fragments and immediately fixed in 2.5% glutaraldehyde (Sinopharm Chemical Reagent Co., Ltd.) at 4 °C for 24 h. After rinsing with 0.1 M phosphate buffer (pH 7.4, 3 × 15 min), specimens were post-fixed in 1% osmium tetroxide (in 0.1 M phosphate buffer, pH 7.4) at 20 °C for 2 h, followed by additional buffer rinses (0.1 M phosphate buffer, pH 7.2; 3 × 15 min). Samples were dehydrated through a graded ethanol series (30%, 50%, 70%, 80%, 85%, 90%, and 100% × 2; 15–20 min per step) and infiltrated with acetone: EPON™ 812 resin (cat# 90529-77-4, SPI) mixtures (at a ratio of 2:1 for 8–12 h followed by a ratio of 1:1 for 8–12 h) at 37 °C, then pure resin overnight. Specimens were then embedded in fresh resin and polymerized at 60 °C for 48 h. Ultra-thin sections (80–100 nm) were cut using a diamond knife (Diatome, Ultra 45°) on a Leica UC7 ultramicrotome (Leica, Germany). Sections were double-stained with 2% uranyl acetate (aqueous) and lead citrate for 15 minutes each at 25 ± 2 °C temperature, then air-dried overnight. Samples were examined under a TECNAI G2 TWIN transmission electron microscope (FEI Company, USA) at 120 kV accelerating voltage.

### Senescence­associated β­galactosidase staining

Doxorubicin­treated cells and their controls were washed with PBS to remove residual medium and then fixed with 4% paraformaldehyde at 25 ± 2 °C temperature for 15 min. After fixation, the cells were rinsed three times with PBS and incubated with staining solution prepared from the senescence­associated β­galactosidase staining kit (C0626, Beyotime) at 37 °C overnight in a constant temperature incubator.

### CCK-8 assay

Cell viability was assessed using a cell counting kit­8 (CCK-8) assay. HUVECs were divided into three groups: the negative control (NC), a doxorubicin­induced senescence group, and a doxorubicin­induced senescence group with stable overexpression of *UBE2M*. Cells were plated in 96­well plates with four technical replicates per condition at a density of 5000 cells per well. At 0, 24, 48, and 72 h, the culture medium in each well was replaced with freshly prepared complete medium containing CCK-8 solution (10 μL CCK­8: 90 μL medium). The plates were incubated at 37 °C for 90 min, and cell viability was assessed by measuring absorbance at 450 nm using a TECAN Sunrise™ microplate reader.

### Wound healing assay

HUVECs were seeded into six­well plates and divided into three groups: the negative control (NC), *Ube2m* knockdown (si*Ube2m*), and *Ube2m* overexpression (oe*UBE2M*) groups. When the cells reached 100% confluence, a 200 μL sterile pipette tip was used to create a uniform scratch across the cell monolayer, forming a clear wound. After scratching, the supernatant was carefully removed, and the wells were washed twice with PBS to remove cell debris. Low­serum medium was then added to inhibit cell proliferation and emphasize migration. The plates were incubated at 37 °C with 5% CO₂, and images of the wound area were captured at 0 h (immediately after scratching) and 16 h to evaluate wound closure and analyze cell migration ability.

### Network formation assay

To assess 2D angiogenic network formation, HUVECs were seeded on Matrix­Gel™ (C0372, Beyotime) coated in 96­well cell culture plates. Each well was prepared with 50 μL of Matrix­Gel™ solidified at 37 °C for 30 min. Subsequently, cells were seeded at a density of 15,000 cells/well, with 100 μL of complete endothelial cell growth medium added to each well. The plates were incubated at 37 °C with 5% CO₂ for 4–12 h to allow capillary­like structures to form. Images of the network structures were captured using an ECHO microscope (#Revolve Gen2) and analyzed using ImageJ software.

### Proximity ligation assay

Cells were transfected with either the VEGFR2 plasmid or an empty plasmid for 48 h. After transfection, the culture medium was discarded, and the cells were fixed with 4% paraformaldehyde at 25 ± 2 °C for 20 min. The fixation solution was removed, and the cells were washed three times with PBS. Permeabilization was carried out using 0.3% Triton X-100 at 25 ± 2 °C for 15 min, followed by blocking with goat serum at 25 ± 2 °C for 1 h. After blocking, the cells were incubated with primary antibodies at 4 °C overnight. The following day, the PLA probes, anti­rabbit PLUS (#DUO92002­100RXN, Sigma) and anti­mouse MINUS (#DUO92004­100RXN, Sigma), were applied and incubated at 37 °C for 1 h. Subsequently, the cells underwent two washes and were treated with ligation solution at 37 °C for 30 min. Amplification was performed using polymerase solution, as instructed by the manufacturer. Finally, the nuclei were stained with DAPI, and images were acquired using an Olympus BX63F microscope or an Olympus FV12­IXCOV laser confocal microscope.

### The elastase-induced COPD model in mice

Our study examined male and female mice, and similar findings are reported for both sexes. Eight-week-old C57BL/6J mice were treated with either 1.2 U of porcine pancreatic elastase or PBS solution (50 µL) via intranasal instillation for four consecutive weeks. Mice were euthanized at the end of the fourth week after elastase treatment to collect lung tissues.

### Tissue section quantification and image analysis

All quantitative analyses were performed using ImageJ software. For vascular wall thickness, HE-stained lung sections were examined, and 10 randomly selected vessels per mouse were measured at 20 equidistant points and averaged. Data were normalized to the control group and expressed as relative thickness. For fibrosis quantification, Masson’s trichrome-stained sections were analyzed by applying a uniform color threshold across all samples within the same staining batch to isolate blue-stained collagen areas, which were converted to binary images. The fibrotic area was quantified as a percentage of the total vascular wall area from 10 randomly selected vessels per mouse, and data were normalized to controls to obtain relative fibrosis. For immunofluorescence, integrated density was measured using a uniform color threshold applied consistently across all samples within the same staining batch to select positive signals. Six randomly selected fields were analyzed per mouse and averaged. Data were normalized to the control group and presented as relative fluorescence intensity.

### Statistical analysis

All statistical analyses were performed using GraphPad Prism v.9.5.0 with normality confirmed by Shapiro–Wilk testing. Two-group comparisons utilized Welch’s t-test (accommodating unequal variances), applying Bonferroni correction for multiple t-tests. Multi-group analyses employed either Welch’s ANOVA or Brown–Forsythe ANOVA followed by Dunnett’s T3 post-hoc testing for pairwise comparisons. CCK-8 proliferation data were analyzed by two-way ANOVA with Geisser–Greenhouse correction using Bonferroni’s method for multiple comparisons, while protein expression data in COPD mouse models underwent two-way ANOVA with Geisser–Greenhouse correction and Tukey-adjusted p-values. Sample sizes for animal experiments were determined based on preliminary experiments and previous publications in the field to ensure adequate statistical power (≥80%). No formal power calculation was performed.

## Results

### UBE2M decreases with pulmonary vascular aging in aged mouse lungs

**Fig. 1:**
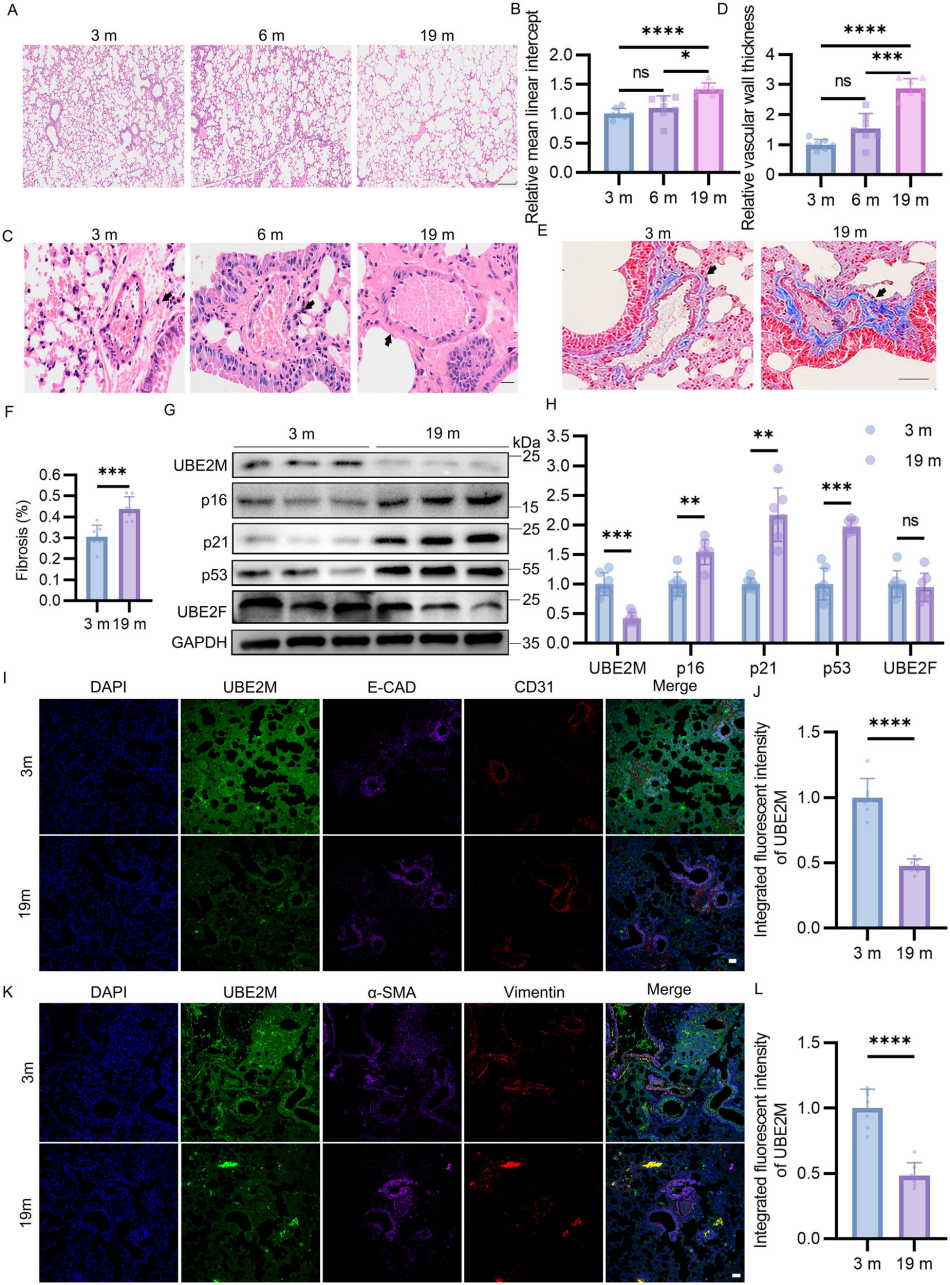
UBE2M decreases in aged mouse lungs with pulmonary vascular aging. **A** Representative microscopic images of HE-stained lung tissues from 3-month, 6-month, and 19-month-old mice, scale bar = 25 μm. **B** Quantification of alveolar linear intercepts as shown in (**A**) with 7 mice per group (4 males and 3 females). **C** Representative microscopic images of lung vasculature in HE-stained lung tissues from 3-month, 6-month, and 19-month-old mice, scale bar = 20 μm. **D** Quantification of vessel wall thickness shown in (**C**) with 7 mice per group (4 males and 3 females) and 6 lung vascular fields selected per mouse. **E** Representative microscopic images of lung vasculature in Masson’s trichrome-stained lung tissues from 3-month and 19-month old mice, scale bar = 20 μm. **F** Quantification of the percentage of elastic fibers in lung vascular walls from **E** with 7 mice per group (4 males and 3 females) and 10 lung vascular fields selected per mouse. **G** Western blot analysis of p16, p21, p53, UBE2M, and UBE2F protein levels in lung tissues from 3-month and 19-month-old mice. **H** Quantification of the grayscale values of Western blot bands shown in (**G**) with 7 mice per group (4 males and 3 females). **I** Representative immunofluorescence images of UBE2M, E-cad, and CD31 in lung tissue from 3-month and 19-month-old mice, scale bar = 50 μm. **J** Quantification of integrated fluorescent intensity of UBE2M in (**I**) with 7 mice per group (4 males and 3 females). **K** Representative immunofluorescence images of UBE2M, α-SMA, and Vimentin in lung tissue from 3-month and 19-month-old mice, scale bar = 50 μm. **L** Quantification of integrated fluorescent intensity of UBE2M in (**K**) with 7 mice per group (4 males and 3 females). The data are presented as the means ± SD. ns, not significant; *P ≤ 0.05; **P ≤ 0.01; ***P ≤ 0.001; ****P ≤ 0.0001.

First, we examined the lung tissue structure of mice of different ages through hematoxylin and eosin (HE) staining. As the mice aged, alveolar expansion became more pronounced, with 19-month-old mice exhibiting the typical emphysema phenotype (Fig. 1A). Statistical analysis of the mean linear intercept of the alveoli (Fig. 1B) showed significant enlargement of the alveoli in 19-month-old mice. Concurrently, the pulmonary vessel walls in 19-month-old mice thickened (Fig. 1C, D), collagen fibers accumulated, and α-smooth muscle actin (α-SMA) expression increased (Fig. 1E, F; Supplementary Fig. 1A, B, D, F). These changes are characteristic of vascular aging and are typically associated with vascular remodeling, smooth muscle cell proliferation, and matrix deposition, which in turn lead to reduced vascular wall elasticity, narrowing of the vessel lumen, and increased blood flow resistance. These alterations ultimately affect vascular permeability and blood supply, impairing oxygen delivery and metabolic function in lung tissue. Notably, UBE2M is widely expressed in various lung tissues, yet its expression level was significantly decreased in the lung tissues of 19-month-old mice (Fig. 1G–L; Supplementary Fig. 1E), suggesting that UBE2M may play a critical role in the lung aging process. Further analysis revealed changes in the transcriptional levels of aging-related markers (p16, p21, IL-1β, and IL-8RA) (Supplementary Fig. 1C) as well as the translational levels of *p16*, *p21*, and *p53* (Fig. 1G, H) in the lung tissue of 19-month-old mice compared with that of 3-month-old mice. These results indicate that UBE2M expression is decreased in the lung tissues of aging mice and may be involved in lung aging.

### *Ube2m* knockdown mice exhibit pulmonary vascular aging and emphysema with reduced VEGFR2 expression and activity

**Fig. 2:**
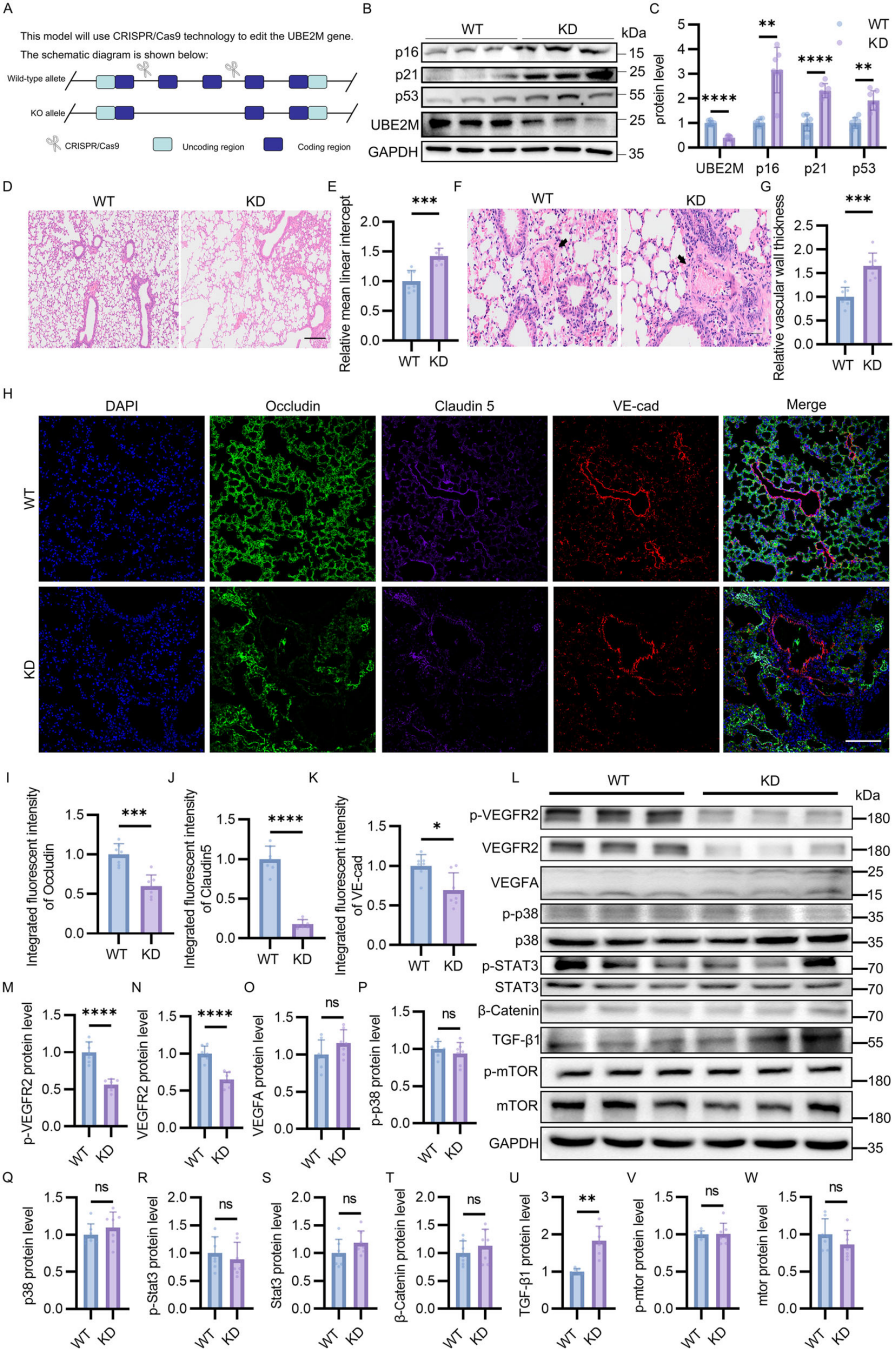
*Ube2m* knockdown mice exhibit pulmonary vascular aging and emphysema with reduced VEGFR2 expression and activity. **A** Schematic diagram of the *Ube2m* knockdown mouse model. **B** Western blot analysis of p16, p21, p53, and UBE2M protein levels in lung tissues from 3-month-old *Ube2m* knockdown and control mice. **C** Quantification of the grayscale values of Western blot bands shown in (**B**) with *n* = 7 mice per group (4 males and 3 females). **D** Representative microscopic images of HE-stained lung tissues from 3-month-old *Ube2m* knockdown and control mice, scale bar = 100 μm. **E** Quantification of alveolar linear intercepts from (**D**) with *n* = 7 mice per group (4 males and 3 females). **F** Representative microscopic images of lung vasculature in HE-stained lung tissues from 3-month-old *Ube2m* knockdown and control mice, scale bar = 20 μm. **G** Quantification of vessel wall thickness shown in (**F**) with *n* = 7 mice per group (4 males and 3 females) and 6 lung vascular fields selected per mouse. **H** Representative immunofluorescence images of Occludin, Claudin 5, and VE-cad in lung tissue from wild-type (WT) and *Ube2m* knockdown (KD) mice, scale bar = 100 μm. **I**–**K** Quantification of integrated fluorescent intensity of Occludin, Claudin 5, and VE-cad in (**H**). **L** Western blot analysis of p-VEGFR2, VEGFR2, VEGFA, p-p38 MAPK, p38 MAPK, p-STAT3, STAT3, β-Catenin, TGF-β1, p-mTOR, and mTOR protein levels in lung tissues from wild-type (WT) and *Ube2m* knockdown (KD) mice. **M**–**W** Quantification of protein expression levels shown in (**L**) with *n* = 7 mice per group (4 males and 3 females). The data are presented as the means ± SD. ns, not significant; *P ≤ 0.05; **P ≤ 0.01; ***P ≤ 0.001; ****P ≤ 0.0001.

We further investigated the role of UBE2M in lung aging and structural abnormalities based on our previous observation that UBE2M protein expression was significantly downregulated in the lung tissue of naturally aged mice. Using CRISPR/Cas9 technology, we generated a *Ube2m* knockdown mouse model (Fig. 2A; Supplementary Fig. 2A). We compared the lung tissue structures of 3-month-old heterozygous knockdown mice and age-matched wild-type mice. Histological analysis through HE staining revealed distinct emphysema features in the lung tissue of *Ube2m* knockdown mice (Fig. 2D, E). In addition, the lung vasculature of *Ube2m* knockdown mice showed thickened vessel walls and collagen accumulation (Fig. 2F, G; Supplementary Fig. 2C–F). These results suggest that UBE2M deficiency exacerbates pathological changes in the lungs and promotes lung aging.

We further assessed key indicators of pulmonary barrier function using immunofluorescence staining. The results showed significantly downregulated expressions of Claudin-5, an endothelial-specific tight junction protein; Occludin, a ubiquitously expressed tight junction protein present in both endothelial and epithelial cells; and VE-cadherin, which mediates endothelial cell–cell adhesion (Fig. 2H–K). The loss of these critical junctional proteins indicates that UBE2M deficiency severely compromises pulmonary barrier integrity.

To further investigate the signaling pathways involved in *Ube2m*-knockdown-mediated lung aging, we performed Western blot analysis on key molecules across multiple potential pathways. The targets included the angiogenic signaling pathway (VEGFA–VEGFR2), stress/senescence-associated pathways (p38 MAPK, p-p38 MAPK; STAT3, p-STAT3; mTOR, p-mTOR), the Wnt/β-catenin pathway (β-catenin), and the fibrosis-related factor TGF-β1 (Fig. 2L–W). Intriguingly, we found that the protein level of VEGFA remained unchanged, whereas the levels of its receptor VEGFR2 and the phosphorylated VEGFR2 (p-VEGFR2) were significantly downregulated. These results suggest that *Ube2m* knockdown may not affect the VEGF ligand but could directly impair VEGFR2 stability. Transcriptional levels of senescence-associated markers, including p21, IL-1β, and IL-8RA (Supplementary Fig. 2B), as well as protein levels of p16, p21, and p53 (Fig. 2B, C), were altered in *Ube2m* knockdown mice, indicating pulmonary tissue aging in these mice. These findings demonstrate that UBE2M deficiency and the consequent downregulation of VEGFR2 are key factors driving the pathological phenotypes of lung aging and emphysema.

### UBE2M deficiency impairs VEGFR2 signaling and promotes aging phenotypes in the pulmonary endothelium

**Fig. 3:**
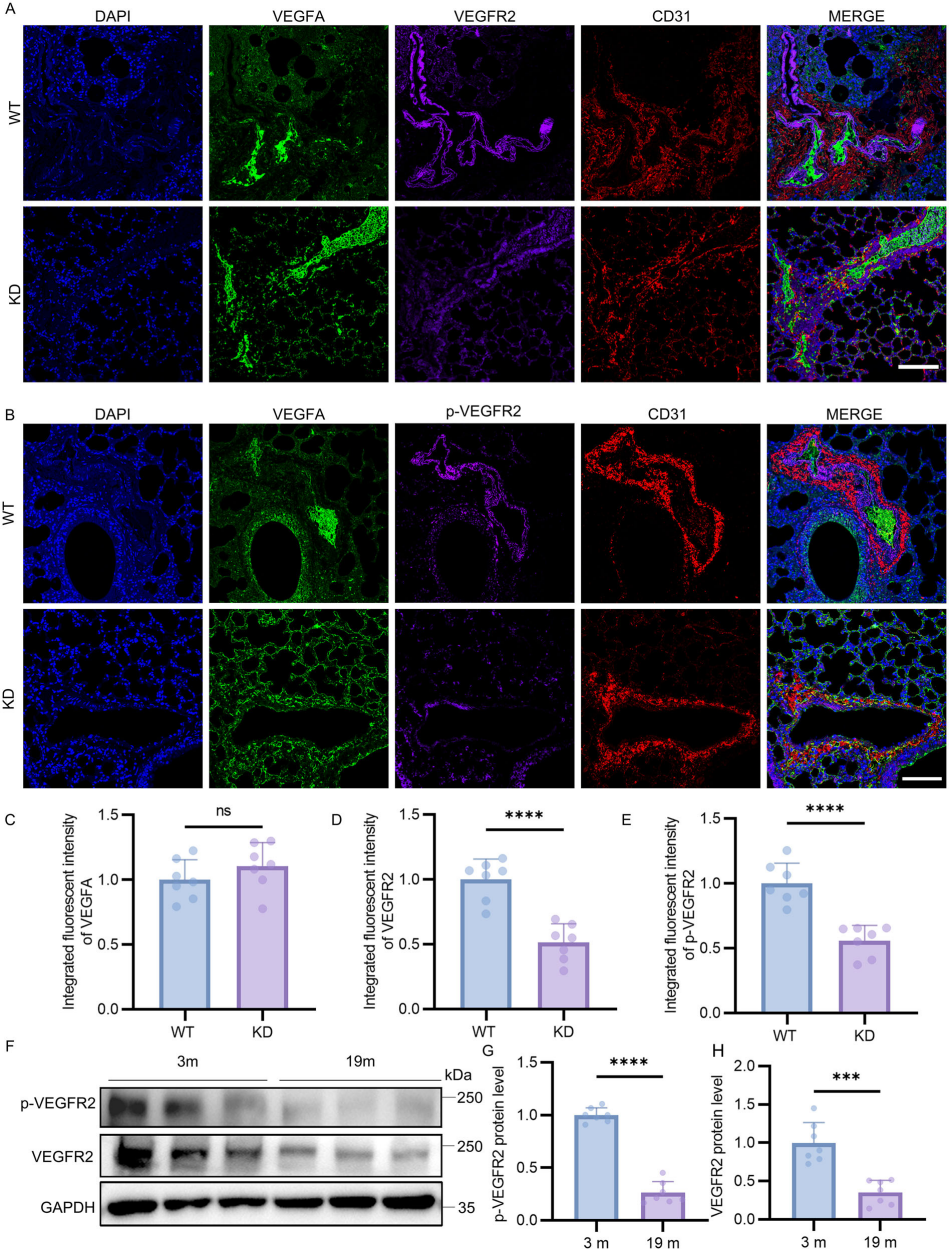
UBE2M deficiency impairs VEGFR2 signaling and promotes aging phenotypes in the pulmonary endothelium. **A** Representative immunofluorescence images of VEGFA, VEGFR2, and CD31 in lung tissue from wild-type (WT) and Ube2m knockdown (KD) mice, scale bar = 100 μm. **B** Representative immunofluorescence images of VEGFA, p-VEGFR2, and CD31 in lung tissue from wild-type (WT) and Ube2m knockdown (KD) mice, scale bar = 100 μm. **C** Quantification of integrated fluorescent intensity of VEGFA in (**A**) with *n* = 7 mice per group (4 males and 3 females). **D** Quantification of integrated fluorescent intensity of VEGFR2 in (**A**) with *n* = 7 mice per group (4 males and 3 females). **E** Quantification of integrated fluorescent intensity of p-VEGFR2 in (**B**) with *n* = 7 mice per group (4 males and 3 females). **F** Western blot analysis of p-VEGFR2 and VEGFR2 protein levels in lung tissues from wild-type (WT) and Ube2m knockdown (KD) mice. **G**–**H** Quantification of protein expression levels shown in (**F**) with *n* = 7 mice per group (4 males and 3 females). The data are presented as the means ± SD. ns, not significant; ***P ≤ 0.001; ****P ≤ 0.0001.

To precisely delineate the role of UBE2M in pulmonary endothelial aging, we first performed immunofluorescence co-staining with the endothelial marker CD31. This analysis revealed a marked reduction in both VEGFR2 and p-VEGFR2 specifically within the pulmonary vascular endothelium of *Ube2m* knockdown mice, while VEGFA expression remained unaltered (Fig. 3A–E). We next conducted a comparative analysis of endothelial aging signatures between naturally aged mice and *Ube2m* knockdown mice. Both groups exhibited upregulated expression of p21 (a key cell cycle arrest and senescence marker) and α-SMA (indicating smooth muscle activation and vascular remodeling), accompanied by downregulation of VE-cadherin (critical for endothelial barrier integrity) and VEGFR2 (essential for endothelial survival and function) (Supplementary Fig. 3A–I).

Consistent with these findings, Western blot analysis confirmed a significant decrease in total VEGFR2 and p-VEGFR2 protein levels in the lung tissues of naturally aged mice (Fig. 3F–H). Given the observed reduction of UBE2M in aged lung tissues, our findings suggest that downregulation of UBE2M may represent a key mechanism underlying impaired VEGFR2 signaling during natural aging. Ultrastructural analysis by transmission electron microscopy provided further evidence of pathological remodeling, showing significantly increased perivascular collagen deposition around pulmonary endothelial cells in 19-month-old mice compared to 3-month-old controls. This age-associated fibrotic change was markedly exacerbated in aged *Ube2m* knockdown mice (Supplementary Fig. 3M).

### UBE2M delays endothelial cell aging, enhances VEGFR2 stability, and improves migration, proliferation, and angiogenesis

To investigate the role of UBE2M in lung vascular endothelial cell aging and its relationship with VEGFR2, we used a doxorubicin-induced endothelial cell aging model. Doxorubicin induces DNA damage and activates cell cycle inhibition pathways, leading to cellular senescence. This model mimics the biological characteristics of endothelial cells during natural aging and provides a reliable experimental basis for identifying key molecular changes.

In this study, we used two in vitro models: primary lung vascular endothelial cells and human umbilical vein endothelial cells (HUVECs). First, we observed nuclear morphology using DAPI staining and found that after doxorubicin treatment, the nuclear volume of endothelial cells increased, which is a hallmark of cellular senescence (Supplementary Fig. 4B, C). Additionally, β-galactosidase staining was performed to quantify the proportion of senescent cells, revealing that 50 nM doxorubicin effectively induced senescence in lung vascular endothelial cells, while 200 nM was the optimal concentration for inducing senescence in HUVECs (Supplementary Fig. 4A, D–F). We also assessed the permeability of HUVECs using an FITC-dextran permeability assay and found that HUVEC permeability increased under aging conditions (Supplementary Fig. 4G, H).

**Fig. 4:**
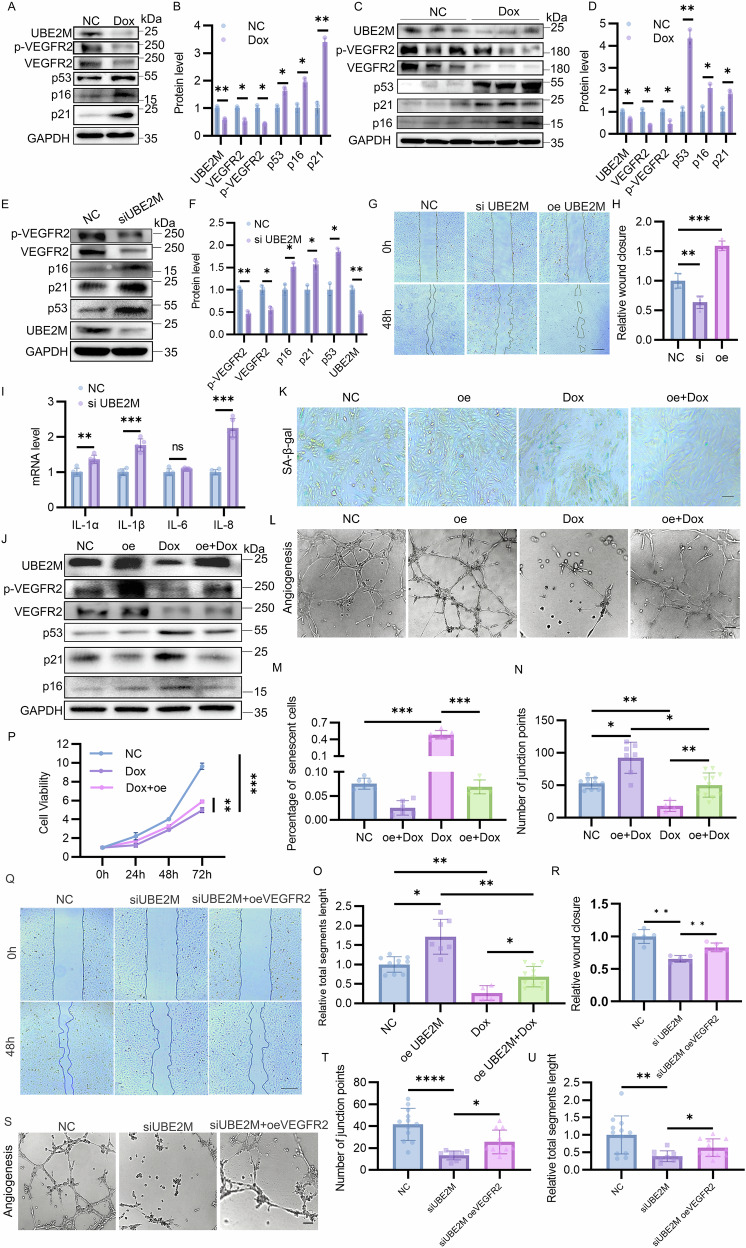
UBE2M delays endothelial aging, stabilizes VEGFR2, and enhances repair functions. **A** Western blot (WB) analysis of UBE2M, VEGFR2, p-VEGFR2, p53, p21, and p16 expression levels in pulmonary vascular endothelial cells at passage 3. **B** Quantification of the grayscale values of WB bands shown in (**A**), *n* = 3. **C** WB analysis of UBE2M, VEGFR2, p-VEGFR2, p53, p21, and p16 expression levels in HUVEC cells at passage 6. **D** Quantification of the grayscale values of Western blot bands shown in (**C**), *n* = 3. **E** WB analysis of p-VEGFR2, VEGFR2, p16, p21, and p53 expression levels in HUVEC cells at passage 6 with Ube2m knockdown. **F** Quantification of the grayscale values of WB bands shown in (**E**), *n* = 3. **G** Scratch assays for HUVEC cells at passage 6 with *UBE2M* knockdown and overexpression, scale bar = 100 μm. **H** Statistical analysis of the change in scratch length from (**G**), *n* = 3. **I** qPCR analysis of IL-1α, IL-1β, IL-6, and IL-8 transcript levels in UBE2M-knockdown HUVEC cells at passage 6, *n* = 5. **J** WB analysis of UBE2M, p-VEGFR2, VEGFR2, p53, p21, and p16 expression levels in stable UBE2M-overexpressing HUVEC cells at passage 6 under doxorubicin induction. **K** Senescence-associated β-galactosidase staining in stable UBE2M-overexpressing HUVEC cells at passage 6 under doxorubicin induction, scale bar = 100 μm. **L** Angiogenesis assay in stable UBE2M-overexpressing HUVEC cells at passage 6 under doxorubicin induction, scale bar = 100 μm. **M** Statistical analysis of the percentage of senescent cells in (**K**), *n* = 6. **N** Statistical analysis of the number of angiogenesis nodes in panel (**L**), *n* = 4–11. (**O**) Statistical analysis of relative angiogenesis length in (**L**), *n* = 4–11. **P** Statistical analysis of cell viability measured by the CCK8 assay in HUVECs at passage 6, *n* = 3. **Q** Scratch assays for HUVEC cells at passage 6 with *UBE2M* knockdown and *UBE2M* knockdown plus VEGFR2 overexpression, scale bar = 100 μm. **R** Statistical analysis of the change in scratch length from panel (**Q**), *n* = 3. **S** Angiogenesis assay in HUVEC cells at passage 6 with Ube2m knockdown and Ube2m knockdown plus VEGFR2 overexpression, scale bar = 100 μm. **T** Statistical analysis of the number of angiogenesis nodes in (**S**), *n* = 4–11. **U** Statistical analysis of relative angiogenesis length in (**S**), *n* = 4–11. The data are presented as the means ± SD. ns, not significant; *P ≤ 0.05; **P ≤ 0.01; ***P ≤ 0.001; ****P ≤ 0.0001.

To further validate the senescent state and associated molecular changes, we assessed the expression of the senescence markers p16, p21, and p53 using Western blotting. The levels of these markers were significantly increased in doxorubicin-treated endothelial cells (Fig. 4A–D). Concurrently, we examined the protein levels of UBE2M, VEGFR2, and p-VEGFR2, and we found that in senescent endothelial cells, UBE2M expression was significantly reduced, and this reduction occurred in parallel with a decrease in the levels of VEGFR2 and p-VEGFR2 (Fig. 4A–D). These findings suggest that UBE2M may play a role in endothelial cell aging by regulating the VEGFR2 pathway and that UBE2M downregulation may contribute to endothelial cell dysfunction.

To investigate the role of UBE2M in endothelial cell aging, we constructed various HUVEC models, including *Ube2m* knockdown and stable overexpression lines, to explore the impact of UBE2M on aging and endothelial cell function. Initially, *UBE2M* expression was knocked down using small interfering RNA (siRNA), resulting in significant aging phenotypes in HUVECs, including an increase in the levels of the aging markers p16 and p21 (Fig. 4E, F). Additionally, markers of the senescence-associated secretory phenotype, such as IL-1α, IL-8, and IL-1β, exhibited significant increases at both transcription levels, suggesting that UBE2M downregulation is linked to endothelial cell aging and inflammation (Fig. 4I).

To verify the protective role of UBE2M against aging, we constructed a stable *UBE2M*-overexpressing HUVEC line and induced aging with doxorubicin. The results showed that *UBE2M*-overexpressing cells exhibited anti-aging properties after doxorubicin treatment. Compared to the control group, the accumulation of p16 and p21 was slower, and aging was delayed (Fig. 4J). Furthermore, VEGFR2 and p-VEGFR2 levels declined more slowly in *UBE2M*-overexpressing cells, suggesting that UBE2M stabilized VEGFR2 and maintained endothelial cell function (Fig. 4J). In contrast, overexpression of the catalytically inactive mutant UBE2M (C111S) failed to rescue VEGFR2 levels (Supplementary Fig. 4I), indicating that UBE2M stabilizes VEGFR2 through its NEDD8-conjugating activity.

We also conducted a series of functional assays, including scratch assays, SA-β-Gal staining, CCK-8 proliferation assays, and angiogenesis assays. In the scratch assay (Fig. 4G, H), *Ube2m* knockdown significantly decreased cell migration, whereas UBE2M overexpression increased the migration speed, highlighting the role of UBE2M in regulating endothelial cell migration.

Next, SA-β-Gal staining revealed that under doxorubicin-induced aging, *UBE2M*-overexpressing cells exhibited significantly less senescence, suggesting that UBE2M overexpression delays aging (Fig. 4K, M). The CCK-8 assay further confirmed the protective role of UBE2M on endothelial cell proliferation (Fig. 4P). In the presence of doxorubicin, *UBE2M*-overexpressing cells had significantly higher proliferation rates than controls, demonstrating that UBE2M partially counteracted the doxorubicin-induced inhibition of cell proliferation. This finding reinforces the importance of UBE2M in maintaining endothelial cell function and proliferative capacity.

We also performed angiogenesis assays (Fig. 4L), in which it was seen that *UBE2M*-overexpressing cells, despite aging, were still able to form blood vessel networks, whereas the control group showed no angiogenesis. Statistical analysis of angiogenic nodes and vessel length showed significant advantages in both parameters for *UBE2M*-overexpressing cells (Fig. 4N, O), further supporting UBE2M’s role in promoting endothelial cell angiogenesis.

To mechanistically link UBE2M with VEGFR2, rescue experiments were conducted using HUVECs. VEGFR2 overexpression partially reversed the impaired migration speed caused by *Ube2m* knockdown in scratch assays (Fig. 4Q, R). Similarly, the compromised network-forming capacity resulting from UBE2M depletion was rescued by VEGFR2 reconstitution (Fig. 4S, T, U).

In conclusion, through a series of in vitro experiments assessing migration, proliferation, aging, and angiogenesis, we found that UBE2M overexpression delays endothelial cell aging and significantly improves migration, proliferation, and angiogenesis, likely through VEGFR2 regulation. These findings suggest that UBE2M not only protects against aging but also supports endothelial cell functioning.

### UBE2M-mediated VEGFR2 neddylation modification enhances VEGFR2 stability

**Fig. 5:**
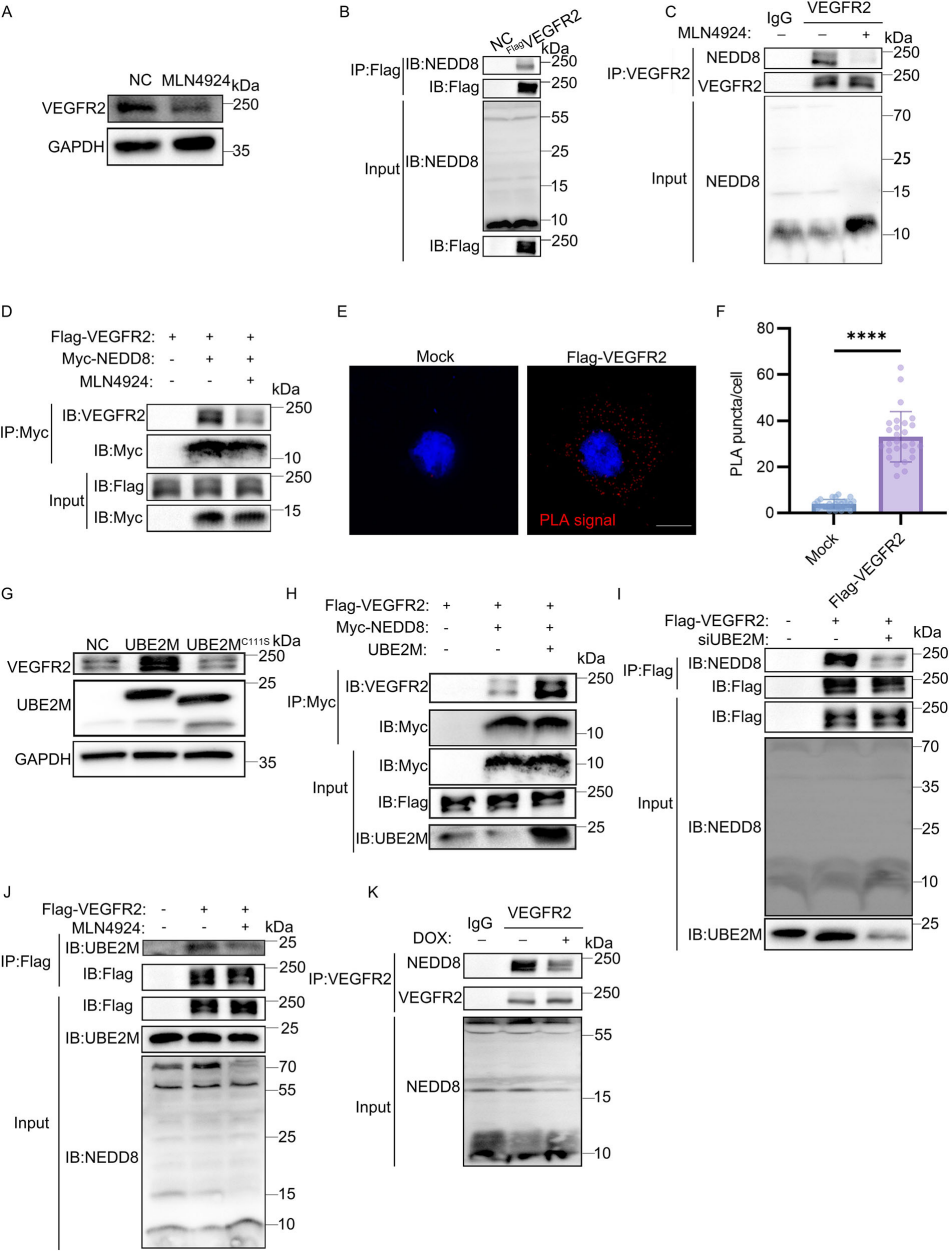
UBE2M-mediated VEGFR2 neddylation modification enhances VEGFR2 stability. **A** HUVEC cells were treated with 1 μM MLN4924, and VEGFR2 protein levels were detected by Western blot (WB). **B** Flag-VEGFR2 was immunoprecipitated from 293 T cells, and the binding of NEDD8 to VEGFR2 was detected. **C** VEGFR2 was immunoprecipitated from HUVEC cells, and the binding of NEDD8 to VEGFR2 was detected with 1 μM MLN4924 treatment. **D** Myc-NEDD8 was immunoprecipitated from 293 T cells, and the binding of Flag-VEGFR2 to Myc-NEDD8 was detected with 0.5 μM MLN4924 treatment. **E** PLA assay was performed in 293 T cells to detect the binding of Flag-VEGFR2 to Myc-NEDD8, with red dots representing PLA signals. **F** PLA signal numbers in individual cells were quantified, *n* = 27. **G** Overexpression of UBE2M and the UBE2M (C111S) mutant in HUVEC cells, and VEGFR2 protein levels were detected by WB. (H) Myc-NEDD8 was immunoprecipitated from 293 T cells, and the binding of Flag-VEGFR2 to Myc-NEDD8 was detected with UBE2M overexpression, enhancing this interaction. **I** Flag-VEGFR2 was immunoprecipitated from 293 T cells, and the binding of NEDD8 to VEGFR2 was detected, with a significant decrease in binding following siUBE2M treatment. **J** Flag-VEGFR2 was immunoprecipitated from 293 T cells, and the binding of UBE2M to VEGFR2 was detected after 0.5 μM MLN4924 treatment. **K** VEGFR2 was immunoprecipitated from HUVEC cells, and the binding of NEDD8 to VEGFR2 was detected with 200 μM Dox treatment. The data are presented as the means ± SD. ****P ≤ 0.0001.

To examine the potential impact of neddylation on the stability of VEGFR2, we first treated HUVECs with the neddylation inhibitor MLN4924. The results showed that VEGFR2 protein levels significantly decreased following MLN4924 treatment (Fig. 5A), suggesting that neddylation may alter VEGFR2 stability in pulmonary vascular endothelial cells.

Immunoprecipitation experiments revealed an interaction between VEGFR2 and NEDD8, indicating that VEGFR2 may be a substrate for neddylation (Fig. 5B). At the endogenous level, the interaction between VEGFR2 and NEDD8 was significantly inhibited upon MLN4924 treatment (Fig. 5C). Further experiments showed that the binding of Myc-tagged NEDD8 to VEGFR2 was weakened after MLN4924 treatment, confirming the occurrence of VEGFR2 neddylation.

Using proximity ligation assay (PLA) technology and confocal microscopy, we observed that VEGFR2–NEDD8 binding signals were primarily located in the cytoplasm (Fig. 5E, F; Supplementary Fig. 5A), suggesting that neddylation may regulate VEGFR2 stability through molecular interactions in the cytoplasm. As an E2 enzyme in the neddylation pathway, UBE2M may play a key role in VEGFR2 neddylation. UBE2M overexpression significantly increased VEGFR2 protein levels, whereas the mutant UBE2M (C111S) did not produce the same effect, indicating that the neddylation transfer ability of UBE2M is crucial for VEGFR2 stability (Fig. 5G).

Immunoprecipitation experiments revealed that UBE2M overexpression enhanced the interaction between VEGFR2 and NEDD8 (Fig. 5H), whereas siUBE2M treatment significantly weakened this interaction (Fig. 5I). Additionally, UBE2M directly interacted with VEGFR2, and this interaction was weakened after MLN4924 treatment (Fig. 5J). In the aging model, the neddylation modification of VEGFR2 was significantly reduced (Fig. 5K). In summary, UBE2M promotes the neddylation of VEGFR2, enhancing its interaction with NEDD8, and thereby increasing VEGFR2 stability.

### RBX1-CUL1 E3 ligase complex mediates UBE2M-dependent neddylation of VEGFR2 at K868 and K871 to protect it from ubiquitination

**Fig. 6:**
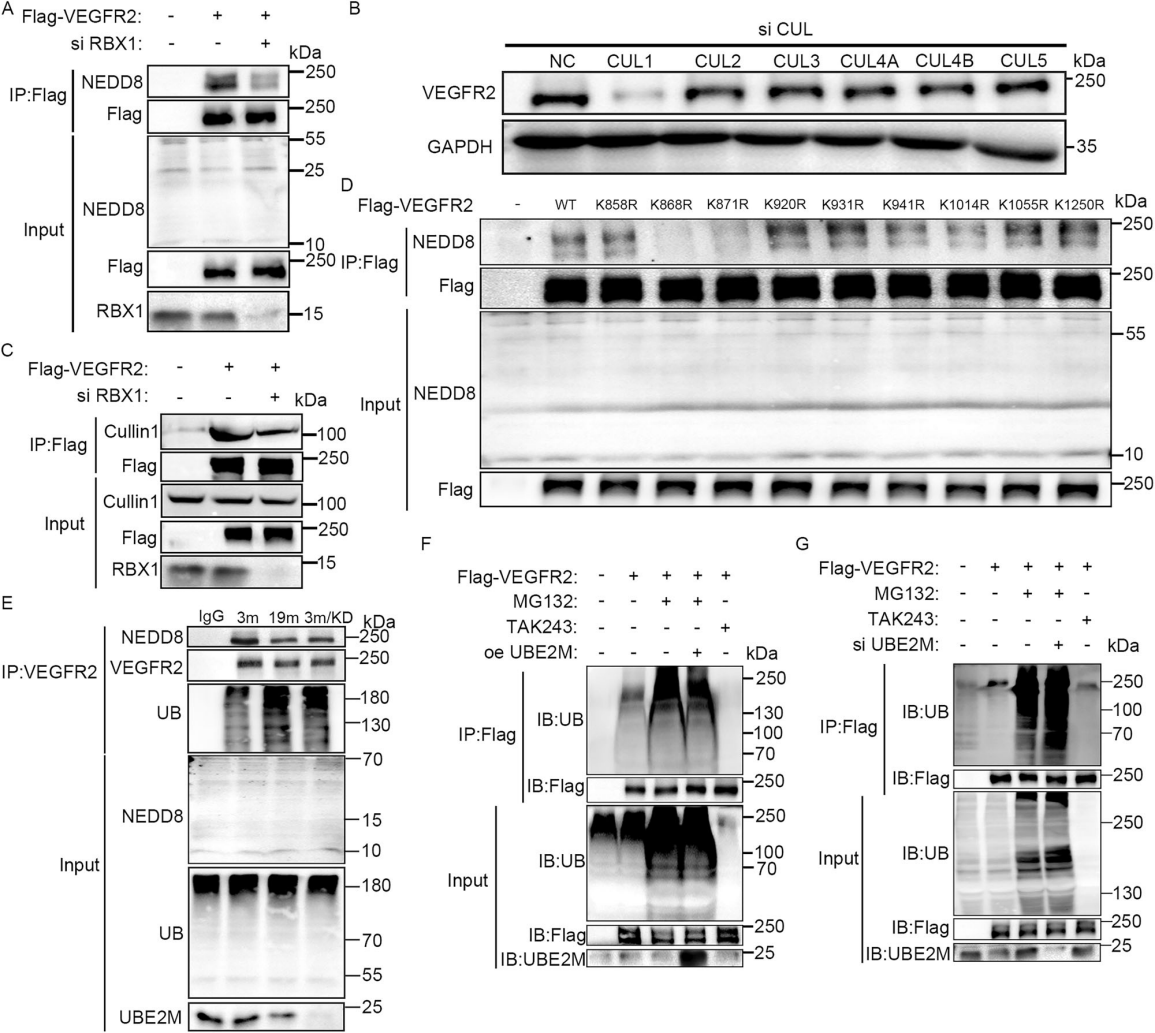
RBX1-CUL1 E3 ligase complex mediates UBE2M-dependent neddylation of VEGFR2 at K868 and K871 to protect it from ubiquitination. **A** 293 T cells were transfected with Flag-VEGFR2, along with control siRNA or siRBX1. Cell lysates were immunoprecipitated with anti-Flag antibody-conjugated magnetic beads, and the neddylation level of VEGFR2 was detected by WB. The effect of RBX1 knockdown on VEGFR2 neddylation was examined. **B** HUVEC cells were transfected with siRNAs targeting CUL1, CUL2, CUL3, CUL4A, CUL4B, and CUL5. VEGFR2 protein expression levels were detected by WB. **C** 293 T cells were transfected with Flag-VEGFR2, along with control siRNA or siRBX1. Cell lysates were immunoprecipitated with anti-Flag antibody-conjugated magnetic beads, and the binding of CUL1 to VEGFR2 was detected by WB. The effect of RBX1 knockdown on this interaction was examined. **D** 293 T cells were transfected with wild-type Flag-VEGFR2 or its lysine-to-arginine point mutants (K858R, K868R, K871R, K920R, K931R, K941R, K1014R, K1055R, K1250R). Cell lysates were immunoprecipitated with anti-Flag antibody-conjugated magnetic beads, and the neddylation levels of wild-type and mutant VEGFR2 were detected by WB. **E** Lung tissue lysates from 3-month-old wild-type mice, 19-month-old wild-type mice, and 3-month-old Ube2m knockdown mice were immunoprecipitated with anti-VEGFR2 antibody-conjugated magnetic beads. IgG immunoprecipitation from 3-month-old wild-type mouse lung tissues served as a negative control. The neddylation and ubiquitination levels of VEGFR2 were detected by WB. **F** 293 T cells were transfected with Flag-VEGFR2, along with a control vector or a UBE2M overexpression plasmid. Cell lysates were immunoprecipitated with anti-Flag antibody-conjugated magnetic beads, and the ubiquitination level of VEGFR2 was detected by WB. TAK243 treatment served as a negative control. **G** 293 T cells were transfected with Flag-VEGFR2, along with control siRNA or siUBE2M. Cell lysates were immunoprecipitated with anti-Flag antibody-conjugated magnetic beads, and the ubiquitination level of VEGFR2 was detected by WB. TAK243 treatment served as a negative control.

To identify the E3 ligase for VEGFR2 neddylation, we first examined RBX1, the RING component of Cullin-RING ligases. Knockdown of RBX1 significantly reduced VEGFR2 neddylation in 293 T cells (Fig. 6A), indicating its involvement. Since RBX1 functions by assembling with different Cullins, we knocked down CUL1, CUL2, CUL3, CUL4A, CUL4B and CUL5 in HUVEC cells. Only CUL1 knockdown markedly decreased VEGFR2 protein levels (Fig. 6B, Supplementary Fig. 5B–H). Co-immunoprecipitation confirmed that VEGFR2 interacts with CUL1, and this interaction was reduced upon RBX1 knockdown (Fig. 6C). These results identify RBX1-CULLIN1 as the E3 ligase for UBE2M-mediated VEGFR2 neddylation. To map the neddylation sites, we generated point mutations at conserved lysines within VEGFR2 intracellular domains (K858R, K868R, K871R, K920R, K931R, K941R, K1014R, K1055R, K1250R). K868R and K871R mutations markedly reduced VEGFR2 neddylation, while other mutations had minimal effects (Fig. 6D). Thus, K868 and K871 are the primary neddylation sites on VEGFR2. Consistent with these findings, the neddylation-deficient mutants VEGFR2^K868R^ and VEGFR2^K871R^ failed to rescue the impaired tube formation caused by *UBE2M* knockdown in HUVECs (Supplementary Fig. S5I–K). We next examined VEGFR2 neddylation in vivo. Compared to 3-month-old wild-type mice, both 19-month-old wild-type mice and 3-month-old Ube2m knockdown mice showed significantly reduced VEGFR2 neddylation along with increased VEGFR2 ubiquitination (Fig. 6E), indicating that age-related UBE2M downregulation impairs this modification. Finally, we assessed the effect of UBE2M on VEGFR2 ubiquitination. UBE2M overexpression decreased VEGFR2 ubiquitination (Fig. 6F), while UBE2M knockdown enhanced it (Fig. 6G), with TAK243 as a negative control. Collectively, UBE2M promotes VEGFR2 neddylation at K868 and K871 via RBX1-CULLIN1, which inhibits VEGFR2 ubiquitination and maintains its stability. This mechanism is compromised during pulmonary aging due to UBE2M downregulation.

### UBE2M reduction exacerbates COPD-associated emphysema and vascular dysfunction

**Fig. 7:**
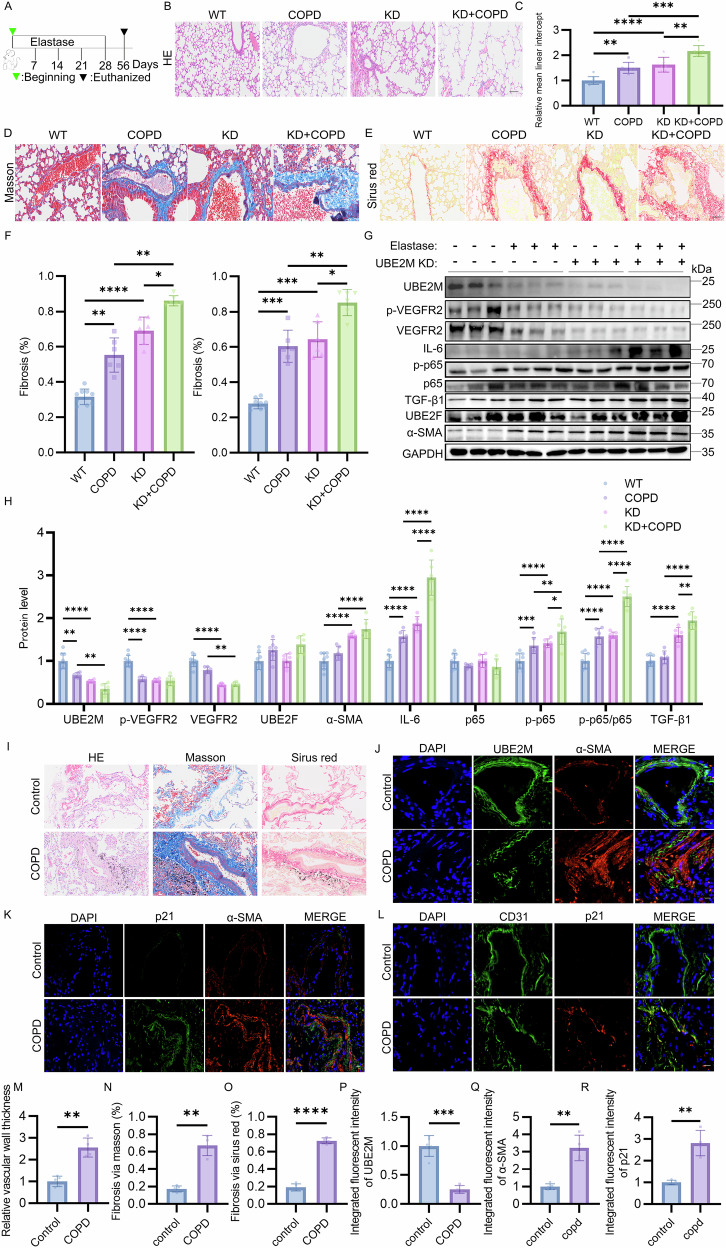
UBE2M reduction exacerbates COPD-associated emphysema and vascular dysfunction. **A** Schematic diagram of the COPD mouse model divided into four groups: WT + PBS, WT + COPD, KD + PBS, and KD + COPD, *n* = 6–9 (WT + PBS: 5 males, 4 females; other groups: 3 males, 3 females). **B** Representative microscopic images of HE-stained lung tissues from the four groups of mice, scale bar = 100 μm. **C** Quantification of the alveolar linear intercepts shown in (**B**), *n* = 6–9. **D** Representative microscopic images of lung vasculature in Masson’s trichrome-stained sections from the four groups of mice, scale bar = 50 μm. **E** Representative microscopic images of lung vasculature in Sirius red-stained sections from the four groups of mice, scale bar = 50 μm. **F** Quantification of collagen fibers in lung vasculature based on Masson’s trichrome and Sirius red staining shown in (**D**) and (**E**), *n* = 6–9. **G** Western blot analysis of UBE2M, p-VEGFR2, VEGFR2, IL-6, p-p65, p65, TGF-β1, UBE2F, and α-SMA protein levels in lung tissues from the four groups of mice. **H** Quantification of protein band intensities from Western blot results shown in (**G**), *n* = 6–9. **I** Representative microscopic images of HE, Masson’s trichrome, and Sirius red-stained lung vasculature from adjacent normal tissues of COPD-related lung cancer patients and lung cancer patients without COPD, scale bar = 100 μm. **J** Representative immunofluorescence images showing co-staining of UBE2M and α-SMA in lung vasculature from adjacent normal tissues of COPD-related lung cancer patients and lung cancer patients without COPD, scale bar = 10 μm. **K** Representative immunofluorescence images showing co-staining of p21 and α-SMA in lung vasculature from adjacent normal tissues of COPD-related lung cancer patients and lung cancer patients without COPD, scale bar = 20 μm. **L** Representative immunofluorescence images showing staining of CD31 and p21 in lung vasculature from adjacent normal tissues of COPD-related lung cancer patients and lung cancer patients without COPD, scale bar = 20 μm. **M**–**O** Statistical analysis of relative vascular wall thickness and fibrosis of pulmonary vessel in (**I**). **P** Quantification of integrated fluorescent intensity of UBE2M in (**J**). (**Q**) Quantification of integrated fluorescent intensity of α-SMA in (**K**). (**R**) Quantification of integrated fluorescent intensity of p21 in (**L**). The data are presented as the means ± SD. *P ≤ 0.05; **P ≤ 0.01; ***P ≤ 0.001; ****P ≤ 0.0001.

To investigate the role of UBE2M in COPD, mice were divided into four groups: wild-type mice treated with PBS, wild-type mice receiving intranasal elastase instillation, *Ube2m* knockdown mice treated with PBS, and *Ube2m* knockdown mice receiving intranasal elastase instillation. COPD models were established through elastase induction (Fig. 7A). HE staining revealed a more pronounced emphysema phenotype in *Ube2m* knockdown mice in the COPD model, indicating that UBE2M deficiency exacerbated emphysema development (Fig. 7B, C). Masson’s trichrome and Sirius Red staining further showed increased collagen fiber deposition in the pulmonary vasculature of *Ube2m* knockdown mice, which could be attributed to vascular wall thickening and excessive elastic fiber accumulation (Fig. 7D–F).

To investigate the underlying mechanisms, we examined the expression of various proteins in the model (Fig. 7G, H). These results demonstrated that UBE2M expression was significantly downregulated in the COPD model, which aggravated the reduction in VEGFR2 expression and activity. Furthermore, evaluation of inflammatory markers, including IL-6, p65, p-p65, and TGF-β1, revealed a markedly enhanced inflammatory response in *Ube2m* knockdown mice under the COPD model, suggesting that UBE2M may regulate COPD pathogenesis through inflammation-related pathways. Immunofluorescence co-staining of UBE2M and α-SMA demonstrated that both COPD and UBE2M downregulation resulted in pulmonary vascular smooth muscle proliferation, closely associated with vascular remodeling and functional impairment (Supplementary Fig. 6A).

To explore the clinical relevance of UBE2M in COPD, we analyzed non-tumor lung tissues from patients with and without COPD. The latter exhibited a sparser pulmonary vascular network (Supplementary Fig. 6B), significantly thickened vascular walls, and increased collagen fiber deposition (Fig. 7I; Fig. 7M–O). Immunofluorescence staining revealed significantly lower VEGFR2 and UBE2M levels in the pulmonary vasculature of patients with COPD, accompanied by vascular smooth muscle proliferation and p21 accumulation, indicating senescence (Fig. 7J–L; Fig. 7P–R; Supplementary Fig. 6C). These findings support the critical role of UBE2M in maintaining pulmonary vascular stability and function.

## Discussion

In this study, we found that, in both natural aging and COPD models, the expression of UBE2M in mouse lung tissue was significantly reduced, revealing its critical role in regulating pulmonary vascular function. Mechanistic investigations demonstrated that UBE2M maintains the stability of VEGFR2 by modulating its neddylation modifications. Functional experiments showed that *Ube2m* knockdown led to endothelial cell senescence and dysfunction, whereas its overexpression confers anti-aging effects. Moreover, *Ube2m*-deficient mice in the COPD model exhibited more pronounced emphysematous changes and increased accumulation of collagen fibers in the pulmonary vasculature. Overall, these results not only uncovered a novel regulatory mechanism––neddylation modification of VEGFR2––but also elucidated the impact of UBE2M on pulmonary vascular function under aging or COPD conditions.

Previous studies predominantly focused on the pro-cancer role of UBE2M. Multiple bioinformatics analyses have shown that *UBE2M* is abnormally overexpressed in highly proliferative tumors, such as lung and liver cancers [18]. Targeting UBE2M can block the neddylation of Cullin proteins, thereby inhibiting the activity of CRL complexes and preventing the ubiquitination-mediated degradation of cell cycle regulatory proteins (p21, p27, and Wee1), ultimately leading to cell cycle arrest in cancer cells [17, 20]. Notably, the functions of UBE2M extend beyond tumorigenesis. As one of only two E2 enzymes in the mammalian neddylation pathway, UBE2M specifically mediates the neddylation of Cullin family members 1–4a, which is essential for maintaining CRL substrate activity.

Recent studies have shown that UBE2M plays a key role in various physiological homeostatic processes. For instance, it sustains the immunosuppressive function of regulatory T cells via the neddylation–CRL axis, with UBE2M deficiency triggering lethal autoimmune responses [21, 22]. Furthermore, UBE2M has been shown to stabilize viral RNA sensor retinoic acid-inducible gene I (RIG-I), thereby modulating antiviral interferon signaling [15]. In addition, the embryonic lethality observed in *Ube2m* knockdown mice further underscores the indispensable role in development and homeostasis. A recent whole-genome CRISPR screen identified neddylation as a key regulator of neuronal aging and neurodegeneration in Alzheimer’s disease [23]. Considering that UBE2M is the core E2 enzyme in the neddylation pathway, its potential role in aging requires further investigation. In this study, we demonstrated for the first time that UBE2M expression is markedly downregulated in the lung tissue and vasculature of aged mice, suggesting that its reduction may contribute to pulmonary aging by affecting protein stability.

Our study further revealed that *Ube2m* knockdown mice exhibit a typical aging phenotype characterized by alveolar expansion. The significant upregulation of senescence markers (p21, p16, p53) and inflammatory factors (IL-1β, IL-8RA) indicates that UBE2M loss accelerates lung aging. In addition, we observed the accumulation of p21 in vascular endothelial cells, along with marked increases in α-SMA and collagen fibers in the pulmonary vessels. These findings are consistent with the theory that endothelial cell senescence impairs vascular remodeling [24] and align with previous reports describing the endothelial network in pulmonary capillaries and arteriovenous vessels as a metabolically active barrier that regulates immune cell migration, vascular tone, and permeability [5, 6].

We also found that both *Ube2m* knockdown mice and 19-month-old mice exhibited downregulation of VEGFR2 and p-VEGFR2. As a key receptor on the surface of endothelial cells, VEGFR2 plays an essential role in angiogenesis and is commonly used as a marker of endothelial function to support cell proliferation and migration [11, 25]. Downregulation of VEGFR2 has been observed under various conditions; for example, in human dermal microvascular endothelial cells, VEGF sends signals through the JNK/c-Jun pathway, inducing endocytosis, nuclear translocation, and downregulation of VEGFR2 via ubiquitination [26]. In aging-related lung diseases such as COPD, elastase-induced models also lead to decreased VEGFR2 expression [10]. Moreover, high glucose conditions promote the ubiquitination of VEGFR2, leading to its degradation [27, 28]. As an NEDD8-conjugating enzyme (E2), UBE2M participates in numerous biological processes by mediating neddylation.

Our findings suggest that UBE2M downregulation reduces VEGFR2 protein levels, likely because of impaired stability. This indicated that UBE2M may stabilize VEGFR2 by inhibiting its ubiquitination and subsequent degradation. Previous studies in breast cancer have shown that UBE2M forms dynamic complexes with CUL3/4A and E6-associated protein (E6AP) to effectively suppress the ubiquitination of estrogen receptor α (ERα), thereby maintaining its stability [29]. Notably, the downregulation of UBE2M observed in natural aging models may alter the dynamic balance between neddylation and ubiquitination, thereby affecting the stability of target proteins. This competitive regulatory mechanism may explain the protective effect of UBE2M on VEGFR2 stability; however, the precise molecular interactions and pathways involved remain unclear.

We further validated the protective role of UBE2M against endothelial senescence using HUVECs. In an Adriamycin-induced senescence model, stable overexpression of *UBE2M* via plasmid transfection significantly reduced the proportion of β-galactosidase-positive cells and suppressed the upregulation of senescence markers such as p21 and p16, suggesting that sustained UBE2M expression can counteract DNA damage-induced senescence. In contrast, the siRNA-mediated knockdown of *Ube2m* induced the accumulation of senescence markers and led to the downregulation of VEGFR2 and p-VEGFR2. These results indicate that UBE2M deficiency may drive endothelial senescence, whereas its overexpression can effectively mitigate this process. VEGFR2 may serve as a key downstream target through which UBE2M downregulation triggers senescence.

Among various post-translational modifications, the phosphorylation, ubiquitination, and acetylation have been extensively studied [25]. For instance, the F-box-containing E3 ubiquitin ligase beta-transducin repeat containing protein 1 (β-TRCP1) mediates the ubiquitination of VEGFR2 [30], and such ubiquitination regulates VEGFR2 stability and clearance via proteasomal degradation or endocytosis/lysosome-dependent pathways [31]. Our study further revealed that VEGFR2 is a novel substrate for neddylation and that its stability is regulated by UBE2M-mediated neddylation modifications. Inhibition of neddylation by MLN4924 resulted in a significant decrease in VEGFR2 protein levels, underscoring the critical role of this modification in maintaining VEGFR2 stability. Co-immunoprecipitation and PLA experiments confirmed an interaction between VEGFR2 and NEDD8, with the binding signal primarily localized in the cytoplasm.

Notably, overexpression of UBE2M enhances the association between VEGFR2 and NEDD8 and increases VEGFR2 protein levels, whereas a catalytically inactive mutant (C111S) does not exhibit such effects [32], indicating that the enzymatic activity of UBE2M directly mediates the modification of VEGFR2. Furthermore, the reduction in VEGFR2 neddylation observed in aging models may provide new molecular insights into aging-related endothelial dysfunction and diminished VEGFR2 signaling, suggesting that dysregulation of neddylation could be a potential mechanism underlying vascular degeneration. In summary, this study provides a new perspective on the post-translational regulation of VEGFR2.

In the context of pulmonary vascular aging, we focused on COPD, a classic obstructive pulmonary disease. Although COPD has traditionally been attributed to smoking, recent studies have revealed that accelerated pulmonary aging is a significant underlying mechanism [33]. Endothelial dysfunction in the pulmonary vasculature plays a critical role in the development and progression of COPD [5, 6]. By establishing a *Ube2m* knockdown mouse model of COPD and analyzing clinical samples, our study demonstrated the central role of UBE2M in maintaining pulmonary vascular homeostasis and in the pathological progression of COPD. UBE2M deficiency significantly exacerbates elastase-induced emphysematous changes, pulmonary vascular collagen deposition, and smooth muscle proliferation, likely due to the loss of UBE2M-mediated VEGFR2 neddylation, which compromises protein stability, thereby impairing endothelial repair capacity and promoting inflammatory responses.

In clinical samples, lung vessels from patients with COPD-associated lung cancer exhibited concomitant downregulation of UBE2M and VEGFR2, along with vascular wall thickening and accumulation of the smooth muscle senescence marker p21, which is highly consistent with the mouse model phenotype. These data demonstrate that the loss of UBE2M-dependent VEGFR2 stabilization is a key driver of pulmonary vascular aging and COPD-related vascular remodeling.

Integrating the “COPD as a clinical syndrome driven by gene–environment interactions” framework proposed in a Lancet review [34] with the GOLD guidelines calling for early pathological intervention [35], our study offers a novel perspective on COPD heterogeneity from the standpoint of post-translational modifications. We propose that UBE2M may serve as a critical regulatory node between environmental stressors (such as smoking and aging) and genetic susceptibility by maintaining VEGFR2 stability to modulate pulmonary vascular development and anti-inflammatory and anti-fibrotic functions. Downregulation or loss of UBE2M expression or activity can lead to vascular barrier disruption, chronic inflammation, and aberrant repair, ultimately leading to a progressive decline in lung function. These findings deepen our understanding of the mechanisms underlying the vascular pathology of COPD.

In summary, our study systematically reveals the key role of UBE2M in the regulation of pulmonary vascular aging. In aged lung tissues, UBE2M is specifically downregulated. By maintaining the neddylation modification of VEGFR2, UBE2M enhances VEGFR2 stability, thus inhibiting the accumulation of endothelial senescence markers and vascular remodeling. Both *Ube2m* knockdown mice and COPD models exhibited diminished VEGFR2 signaling, disrupted alveolar structure, and exacerbated inflammation, and clinical samples further confirmed the association between UBE2M expression and pulmonary vascular aging. This work not only uncovers a novel mechanism by which UBE2M maintains vascular homeostasis but also provides a new strategy for targeting UBE2M in the treatment of aging-related pulmonary diseases.

## Supplementary information

Supplementary materials

Full and uncropped western blots

## Data Availability

All data needed to evaluate the conclusions in the paper are present in the paper. Additional data related to this paper may be requested from the corresponding author.
