# Supplementary material for: UBE2M-mediated neddylation modification stabilizes VEGFR2 to delay pulmonary vascular endothelial cell senescence
Source: Cell Death Dis. 2026 May 28;17(1):659. doi: 10.1038/s41419-026-08881-0 (PMC13402360; doi:10.1038/s41419-026-08881-0)
Supplement: Supplementary file 1 — Supplementary materials [file 41419_2026_8881_MOESM1_ESM.doc]

Table S1 Primer sequences used for RT­qPCR

| Gene | Forward primer | Reverse primer | product length |
| --- | --- | --- | --- |
| *Actin*(mouse) | 5’CATTGCTGACAGGATGCAGAAGG3’ | 3’TGCTGGAAGGTGGACAGTGAGG5’ | 138bp |
| *p16*(mouse) | 5’TGTTGAGGCTAGAGAGGATCTTG3’ | 3’CGAATCTGCACCGTAGTTGAGC5’ | 114bp |
| *p21*(mouse) | 5’TCGCTGTCTTGCACTCTGGTGT3’ | 3’CCAATCTGCGCTTGGAGTGATAG5’ | 124bp |
| *Il8RA*(mouse) | 5’CCATTCCGTTCTGGTACAGTCTG3’ | 3’GTAGCAGACCAGCATAGTGAGC5’ | 129bp |
| *Il1β*(mouse) | 5’TGGACCTTCCAGGATGAGGACA3’ | 3’GTTCATCTCGGAGCCTGTAGTG5’ | 148bp |
| *ACTIN*(human) | 5’CACCATTGGCAATGAGCGGTTC3’ | 3’AGGTCTTTGCGGATGTCCACGT5’ | 135bp |
| *IL1β*(human) | 5’CCACAGACCTTCCAGGAGAATG3’ | 3’GTGCAGTTCAGTGATCGTACAGG5’ | 131bp |
| *IL1α*(human) | 5’TGTATGTGACTGCCCAAGATGAAG3’ | 3’AGAGGAGGTTGGTCTCACTACC5’ | 96bp |
| *IL6*(human) | 5’AGACAGCCACTCACCTCTTCAG3’ | 3’TTCTGCCAGTGCCTCTTTGCTG5’ | 132bp |
| *IL8*(human) | 5’GAGAGTGATTGAGAGTGGACCAC3’ | 3’CACAACCCTCTGCACCCAGTTT5’ | 112bp |
| *CUL1*(human) | 5’CAATGACGCTGGCTTTGTGGCT3’ | 3’CAAGGAGTCACAGTATCGAGCC5’ | 130bp |
| *CUL2*(human) | 5’GTCTTACTCCGTGCTGTGTCCA3’ | 3’CTGACTCCACAAATAGTGTTGGC5’ | 133bp |
| *CUL3*(human) | 5’TCGACAGCTCACACTCCAGCAT3’ | 3’GTGCTTCCGTGTATTAGAGCCAG5’ | 136bp |
| *CUL4A*(human) | 5’GAATGAGCGGTTCGTCAACCTG3’ | 3’CTGTGGCTTCTTTGTTGCCTGC5’ | 131bp |
| *CUL4B*(human) | 5’GAAGCTACAGATGAAGAACTTGAG3’ | 3’GCACTCTTTCCGACTAACAGGC5’ | 134bp |
| *CUL5*(human) | 5’CCTGATGCTGAACTTAGGAGGAC3’ | 3’GGTTCACTGAGAAGAGGGTACC5’ | 133bp |

**Supplementary Figures:**


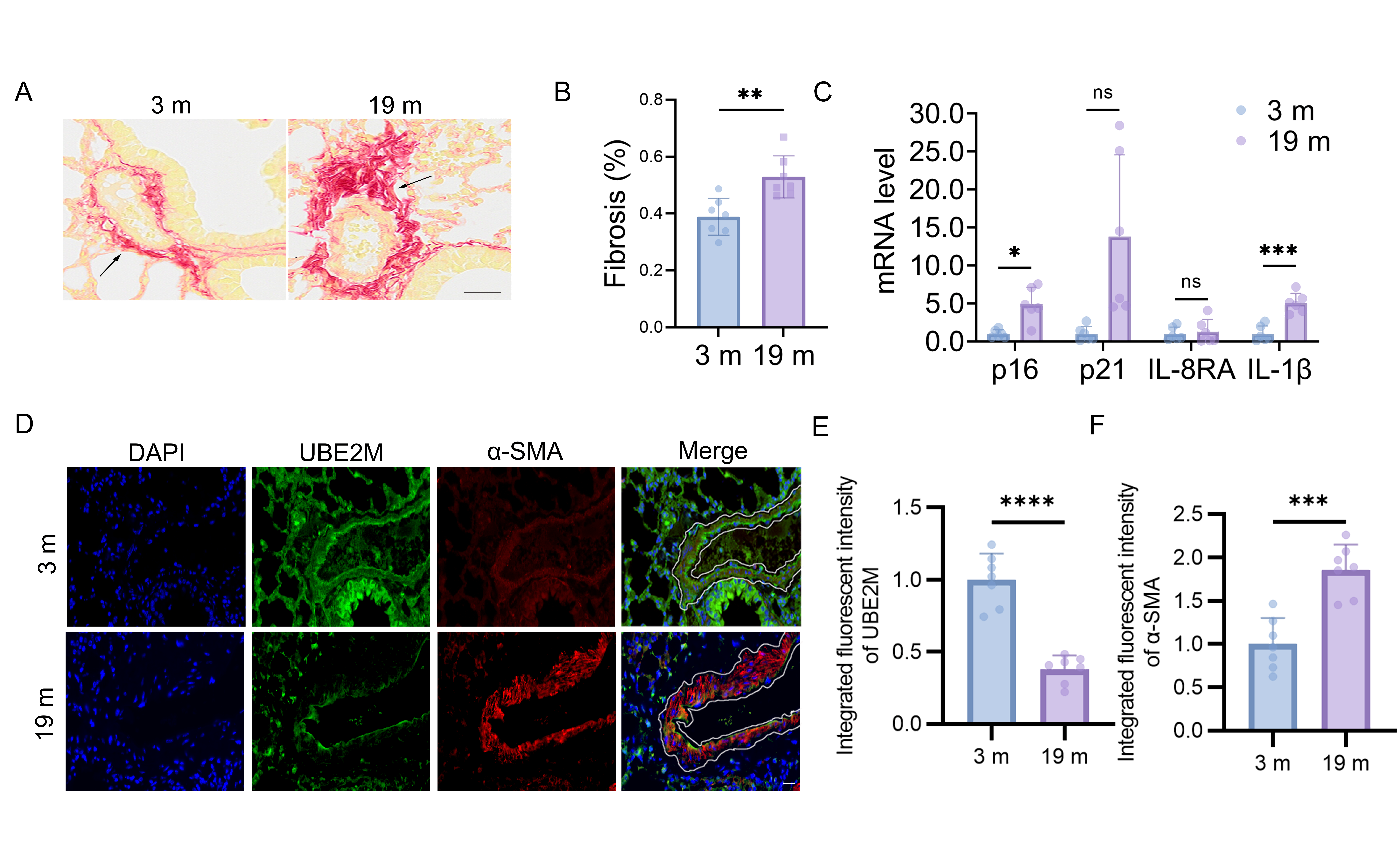


Figure S1 (A) Representative microscopic fields of lung vasculature Sirius red staining in 3-month-old and 19-month-old mice, scale bar = 25 μm. (B) quantitative analysis of collagen fibers shown in (A), n=7 (4 males and 3 females). (C) quantitative analysis of the transcriptional levels of p16, p21, IL-8RA, and IL-1β in 3-month-old and 19-month-old mice, n=6 (3 males and 3 females). (D) Representative immunofluorescence images of UBE2M and α-SMA in lung tissue from 3-month and 19-month old mice, scale bar = 20 μm. (E)-(F) Quantification of integrated fluorescent intensity of UBE2M and α-SMA in (D) (4 males and 3 females per group). The data are presented as the means ± SD. ns, not significant, *P≤0.05, **P≤0.01, ***P≤0.001, ****P≤0.0001.

**

**

Figure S2 (A) Genotyping results of UBE2M knockdown and control mice based on tail samples. The knockdown mice displayed two bands at 288 bp and 1435 bp, whereas the control group showed only a single band at 1435 bp. (B) Transcriptional levels of IL-8RA, IL-1β, and p21 in the lung tissues of UBE2M knockdown and control mice.n=6 (3 males and 3 females). (C) Representative microscopic images of Masson's trichrome staining of lung vasculature from UBE2M knockdown and control mice, n=6 (3 males and 3 females), scale bar = 50 μm (D) Quantification of collagen fibers from Masson's trichrome staining shown in (C), n=6 (3 males and 3 females). (E) Representative microscopic images of Sirius red staining of lung vasculature from UBE2M knockdown and control mice, scale bar = 20 μm. (F) Quantification of collagen fibers from Sirius red staining shown in (E), n=6 (3 males and 3 females).The data are presented as the means ± SD. ns, not significant, *P≤0.05, **P≤0.01, ***P≤0.001, ****P≤0.0001.





Figure S3 (A) Representative immunofluorescence images of VE-CAD and p21 in the lung vasculature of 3-month-old and 19-month-old mice, scale bar 20um. (B) Representative immunofluorescence images of VE-CAD and p21 in the lung vasculature of UBE2M knockdown and control mice, scale bar 10um. (C) Representative immunofluorescence images of VEGFR2 and α-SMA in the lung vasculature of 3-month-old and 19-month-old mice, scale bar = 20um. (D) Representative immunofluorescence images of VEGFR2 and α-SMA in the lung vasculature of UBE2M knockdown and control mice, scale bar = 20um. (E) Quantification of integrated fluorescent intensity of VE-CAD in (A) (4 males and 3 females per group). (F) Quantification of integrated fluorescent intensity of p21 in (A) (4 males and 3 females per group). (G) Quantification of integrated fluorescent intensity of p21 in (B) (4 males and 3 females per group). (H) Quantification of integrated fluorescent intensity of VEGFR2 in (C) (4 males and 3 females per group). (I) Quantification of integrated fluorescent intensity of α-SMA in (D) (4 males and 3 females per group). (J) Transmission electron microscopy (TEM) images of perivascular elastic fibers adjacent to endothelial cells in lung tissue from 3-month-old wild-type (3m WT), 3-month-old UBE2M-knockdown, 19-month-old wild-type, and 19-month-old UBE2M-knockdown mice. Scale bar = 2 μm. The data are presented as the means ± SD. ***P≤0.001, ****P≤0.0001.


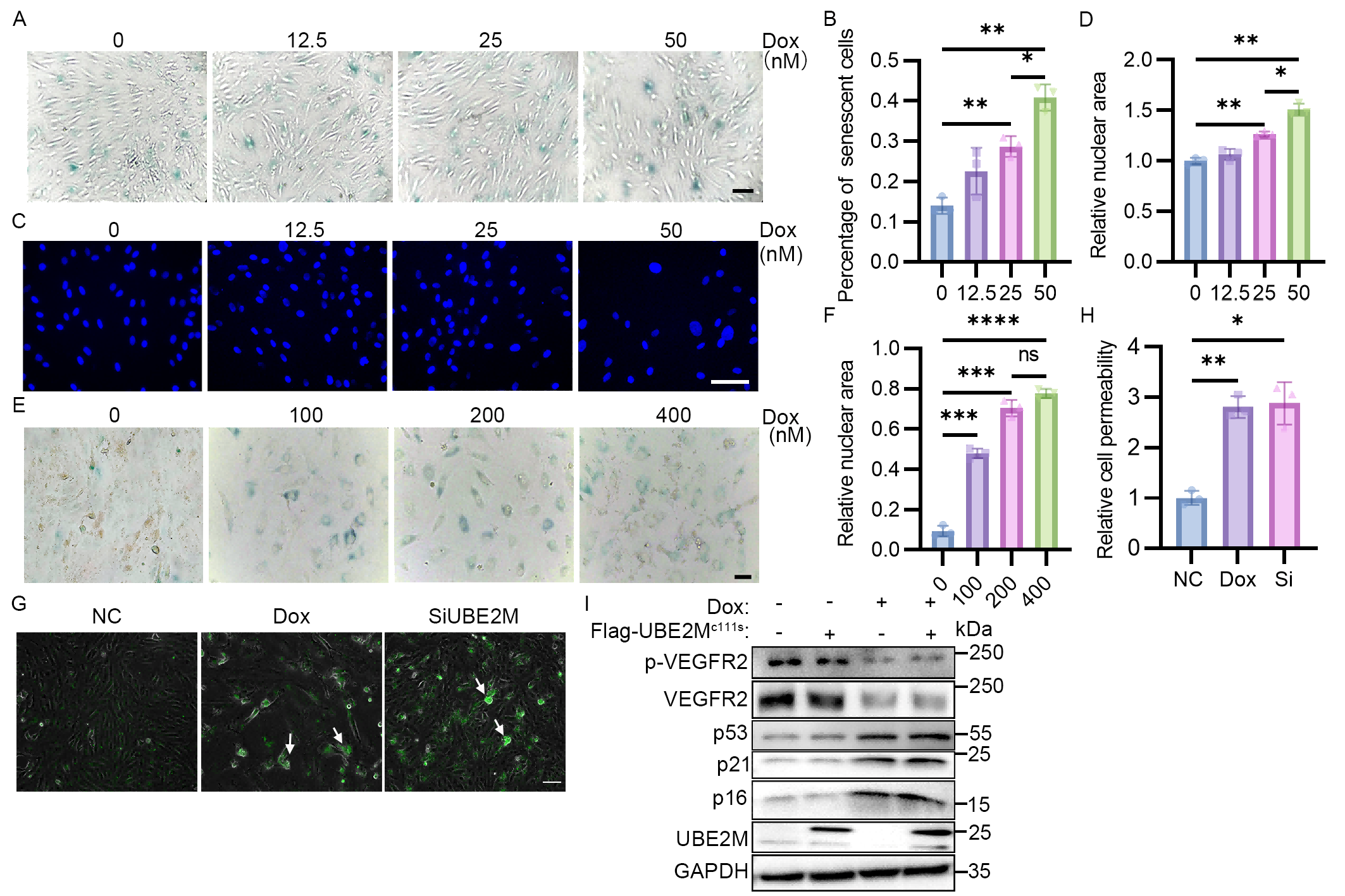


Figure S4 (A) SA-β-Gal staining shows aging of pulmonary vascular endothelial cells at passage 3, scale bar 100μm. (B) Quantitative analysis of the proportion of aging pulmonary vascular endothelial cells at passage 3, n=3. (C) DAPI staining shows the nuclear morphology of pulmonary vascular endothelial cells at passage 3, scale bar 100μm. (D) Quantitative analysis of the nuclear size of pulmonary vascular endothelial cells at passage 3, n=3. (E) SA-β-Gal staining shows aging of HUVEC cells at passage 6, scale bar 100μm. (F) Quantitative analysis of the proportion of aging HUVEC cells at passage 6, n=3. (G) Representative microscopic images of doxorubicin (200nM)-induced senescence and UBE2M-knockdown in HUVECs at passage 6, with green indicating FITC-labeled dextran, scale bar = 100 μm. (H) quantitative analysis of relative cellular permeability in (G). (I) WB analysis of UBE2M, p-VEGFR2, VEGFR2, p53, p21, and p16 expression levels in ^Flag^UBE2M^c111s^-overexpressing HUVEC cells at passage 6 under doxorubicin induction. The data are presented as the means ± SD. ns, not significant, *P≤0.05, **P≤0.01, ***P≤0.001, ****P≤0.0001.





Figure S5 (A) Co-localization of NEDD8 and VEGFR2 in HUVEC cells at passage 6, scale bar = 10 μm. (B)-(G) Knockdown of RBX1-related CUL family members in HUVEC cells, quantitative analysis of knockdown efficiency by qPCR, n=4. (H) Quantitative analysis of VEGFR2 protein expression in cul1-knockdown HUVEC cells detected by Western blot, n=4. (I) Angiogenesis assay in HUVEC cells at passage 6 with Ube2m knockdown and Ube2m knockdown plus VEGFR2^K868R^ or VEGFR2^K871R^ overexpression, scale bar = 200 μm. (J)-(K) (T) Statistical analysis of the number of angiogenesis nodes in panel (I), n = 8. (U) Statistical analysis of relative angiogenesis length in panel (I), n =8. The data are presented as the means ± SD. *P≤0.05, **P≤0.01.





Figure S6 (A) Immunostaining of UBE2M and α-SMA in lung vasculature of the mouse COPD model, scale bar = 20 μm. (B) CD31 staining of adjacent normal tissue from COPD-related lung cancer patients and adjacent normal tissue from lung cancer patients without COPD, scale bar = 100 μm. (C) Immunostaining of VEGFR2 and α-SMA in adjacent normal tissue from COPD-related lung cancer patients and adjacent normal tissue from lung cancer patients without COPD, scale bar = 100 μm. (D)-(E) Quantification of integrated fluorescent intensity of UBE2M and α-SMA in (A). (F) Quantification of integrated fluorescent intensity of CD31 in (B). (G) Quantification of integrated fluorescent intensity of VEGFR2 in (C).
