# Supplementary material for: UBE2M-mediated neddylation modification stabilizes VEGFR2 to delay pulmonary vascular endothelial cell senescence
Source: Cell Death Dis. 2026 May 28;17(1):659. doi: 10.1038/s41419-026-08881-0 (PMC13402360; doi:10.1038/s41419-026-08881-0)
Supplement: Supplementary file 2 — Full and uncropped western blots [file 41419_2026_8881_MOESM2_ESM.pdf]

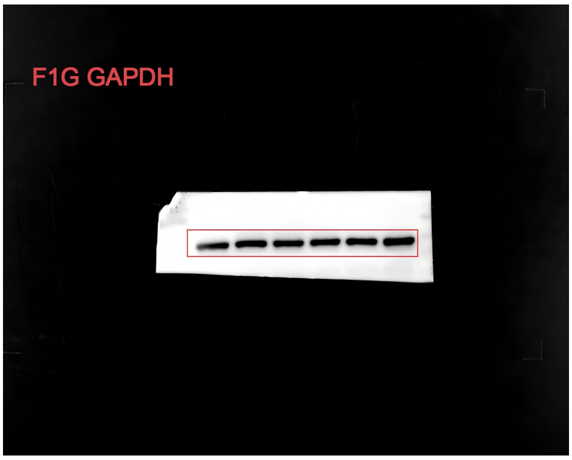
F1G GAPDH

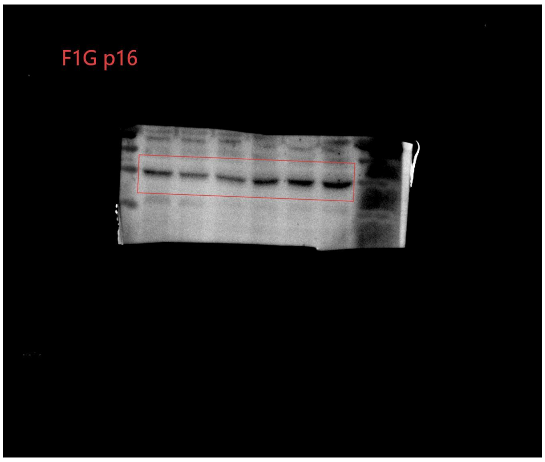
F1G p16

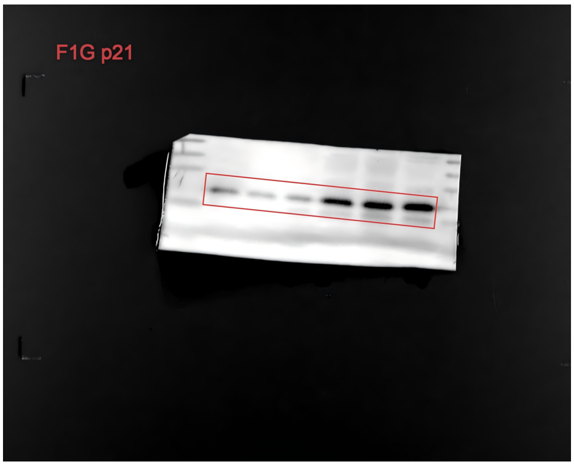
F1G p21

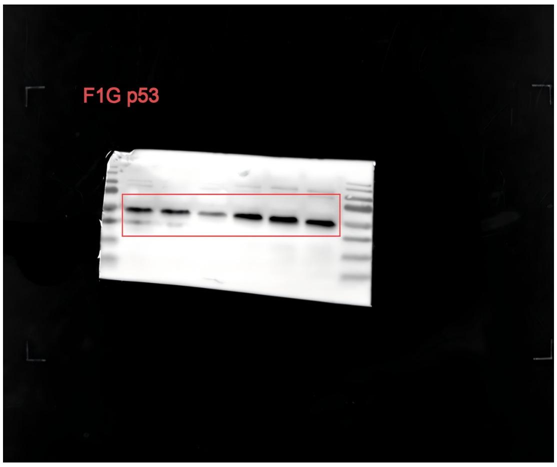
F1G p53

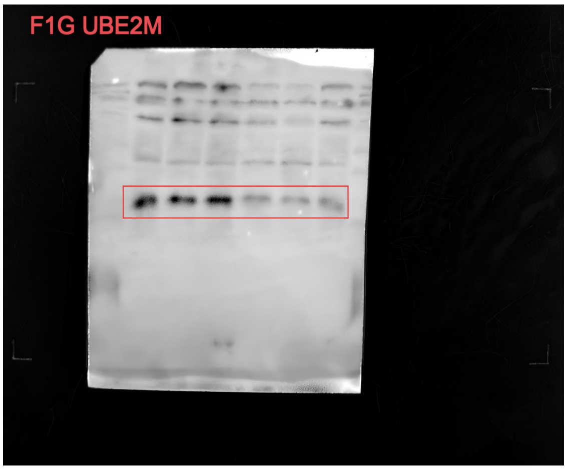
F1G UBE2M

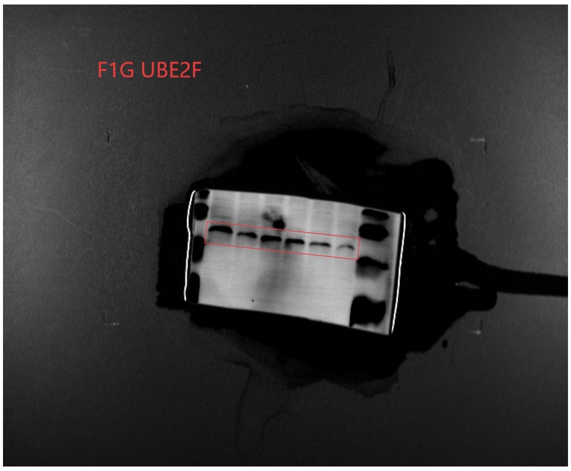
F1G UBE2F

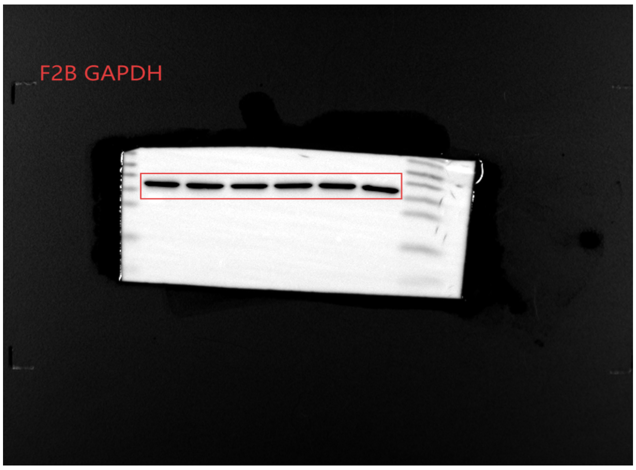
F2B GAPDH

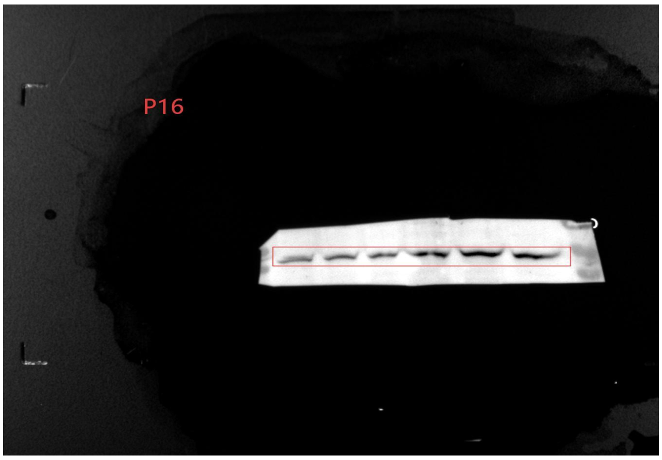
P16

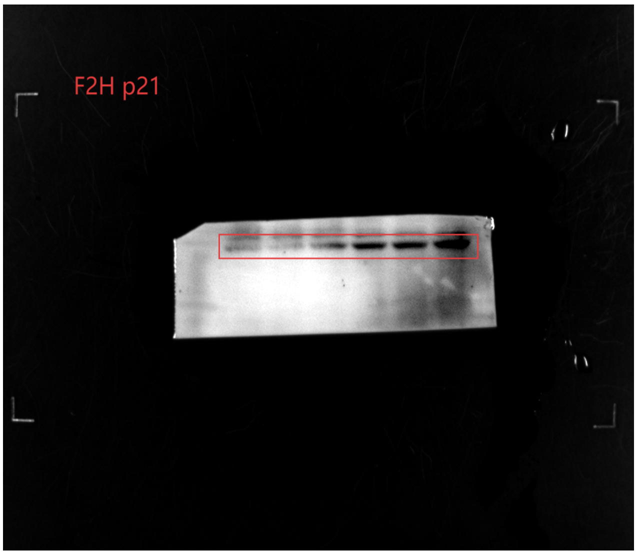
F2H p21

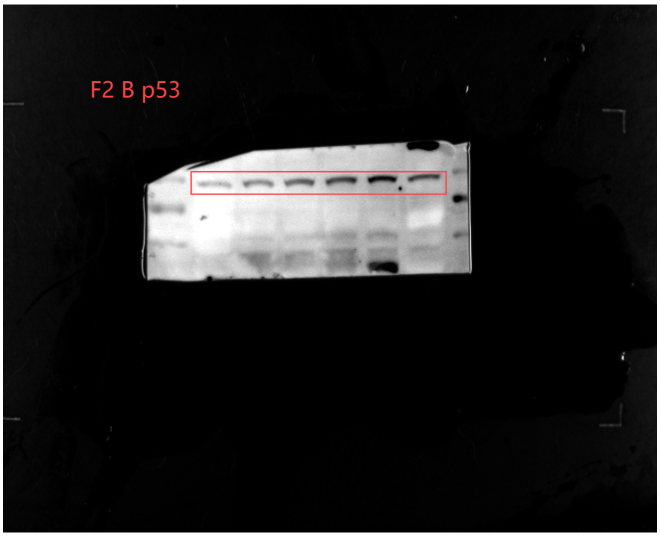
F2 B p53

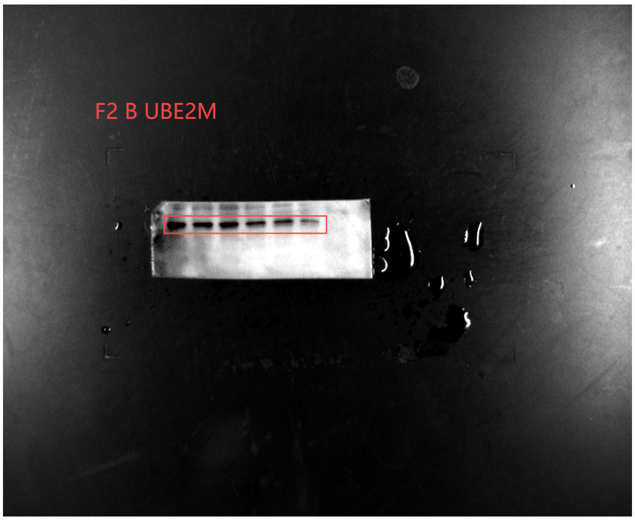
F2 B UBE2M

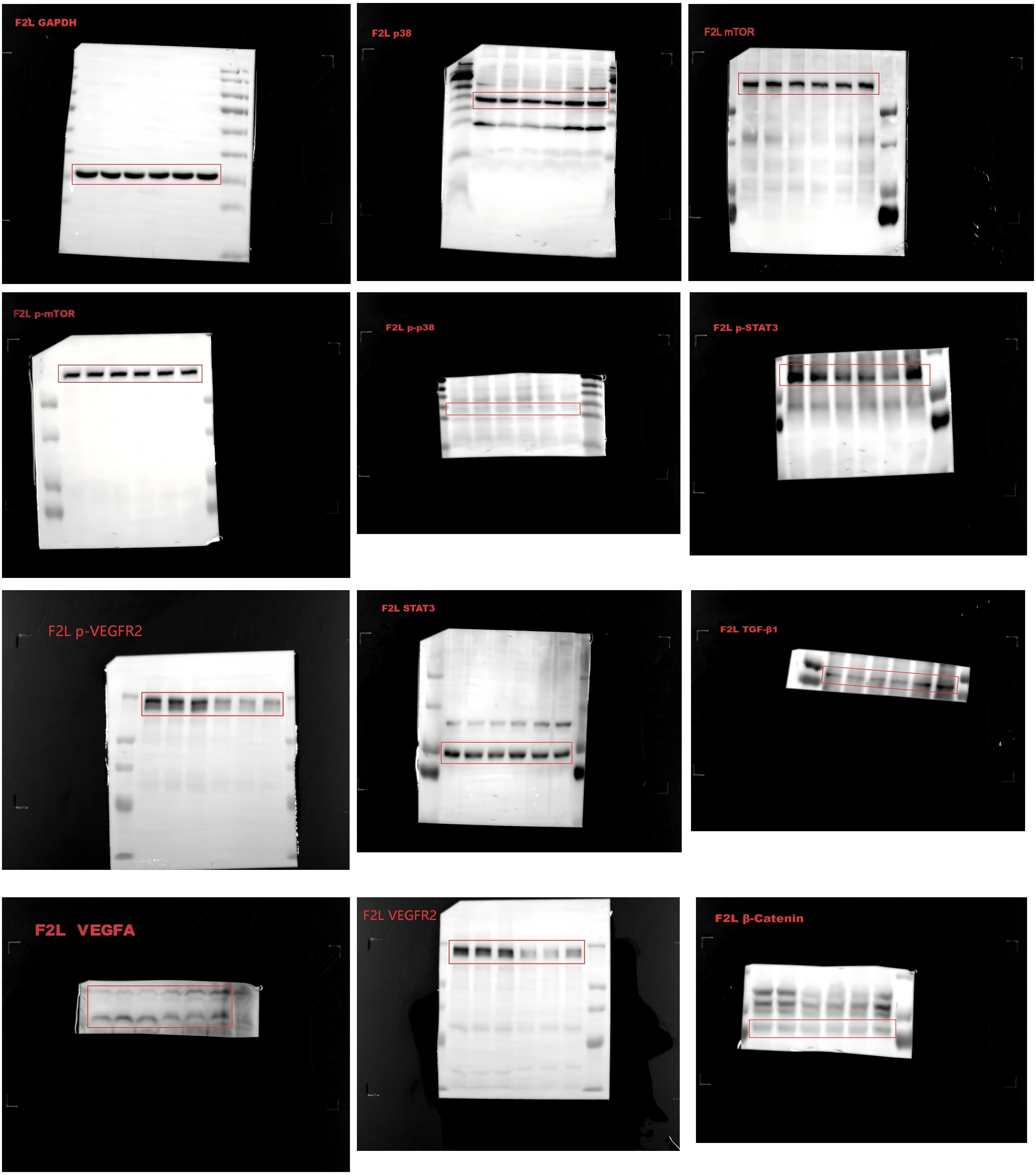
F2L GAPDH
F2L p38
F2L mTOR
F2L p-mTOR
F2L p-p38
F2L p-STAT3
F2L p-VEGFR2
F2L STAT3
F2L TGF-β1
F2L VEGFA
F2L VEGFR2
F2L β-Catenin

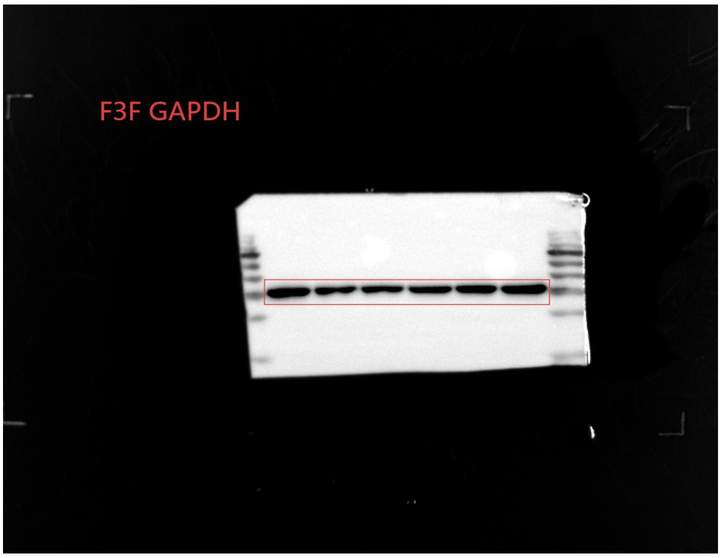
F3F GAPDH

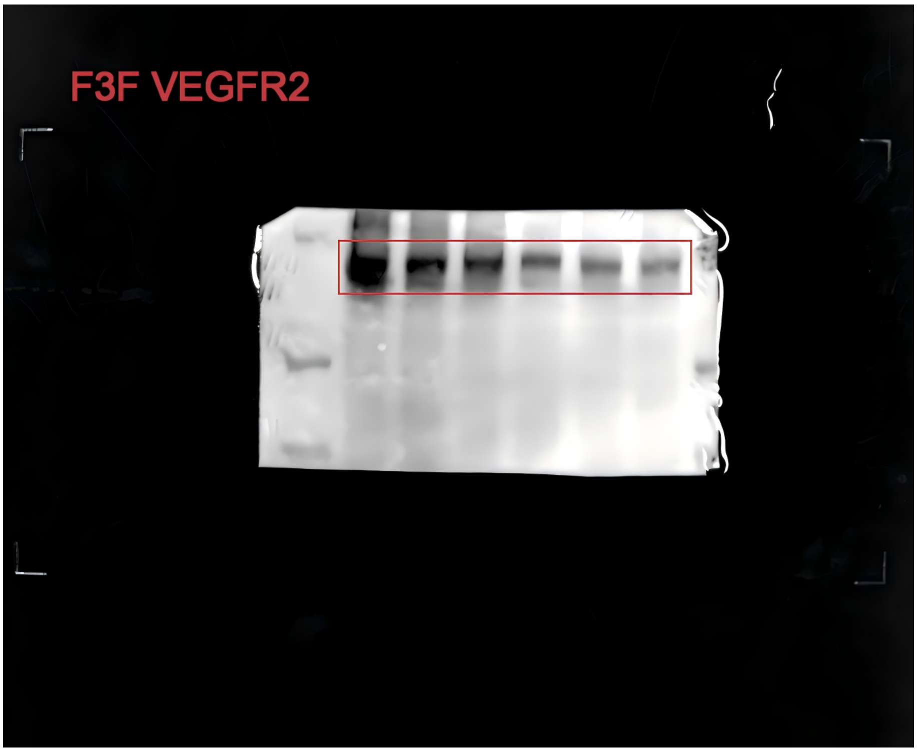
F3F VEGFR2

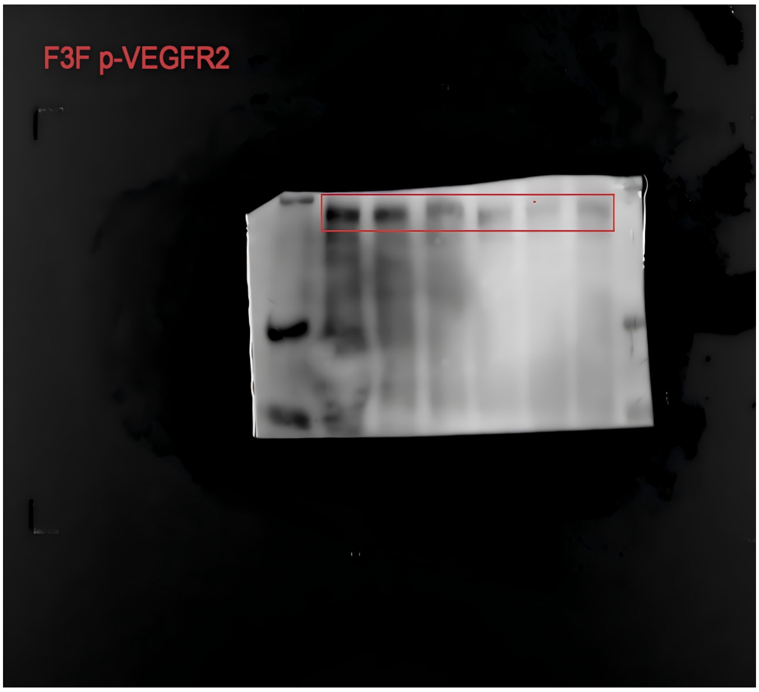
F3F p-VEGFR2

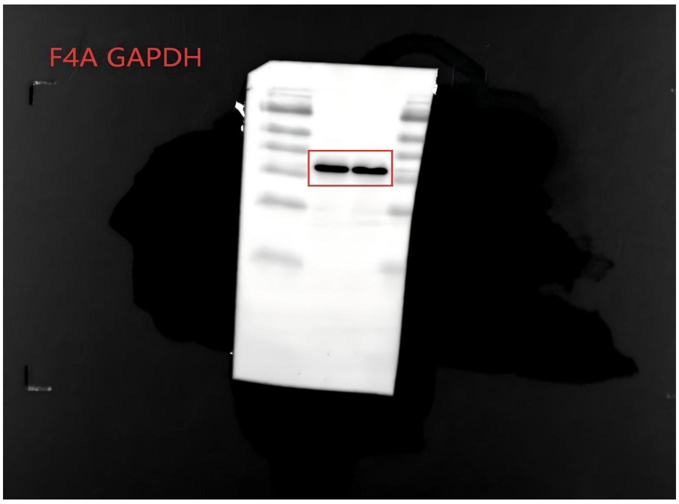
F4A GAPDH

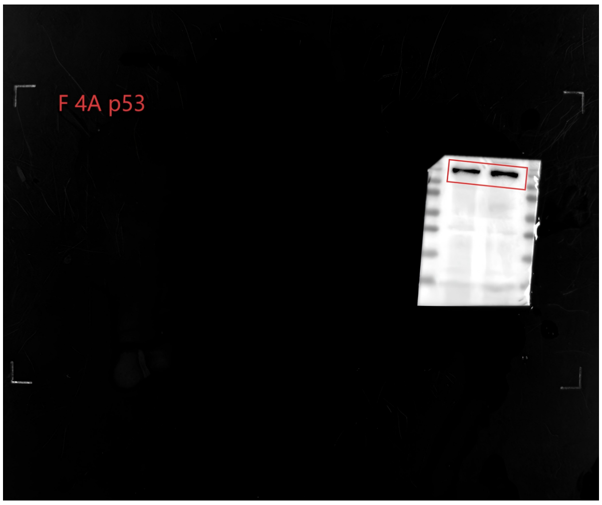
F 4A p53

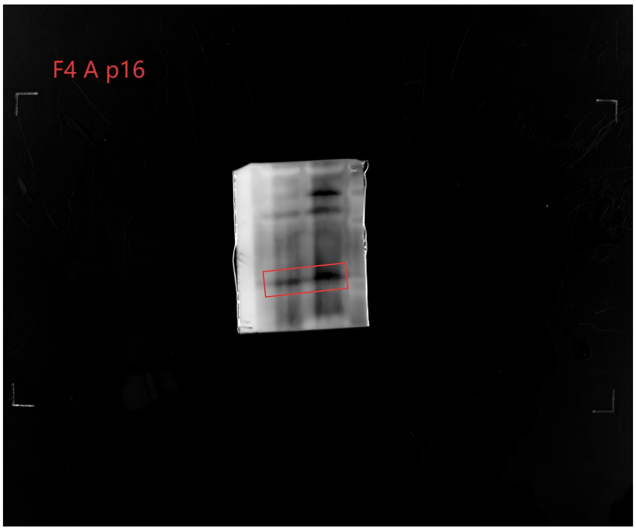
F4 A p16

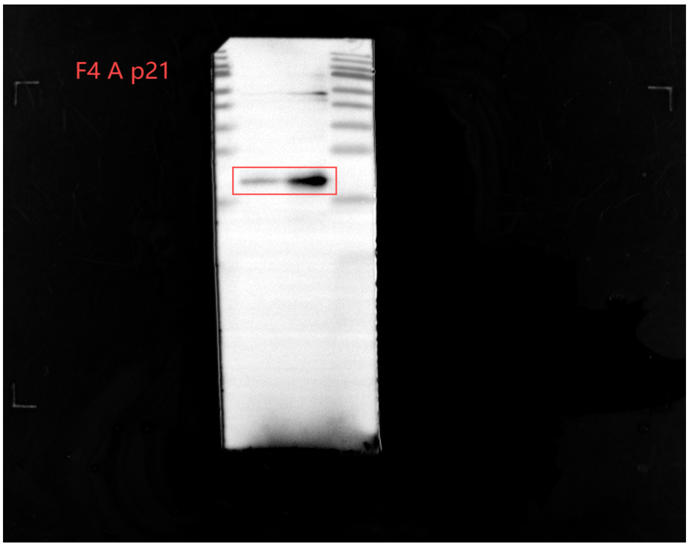
F4 A p21

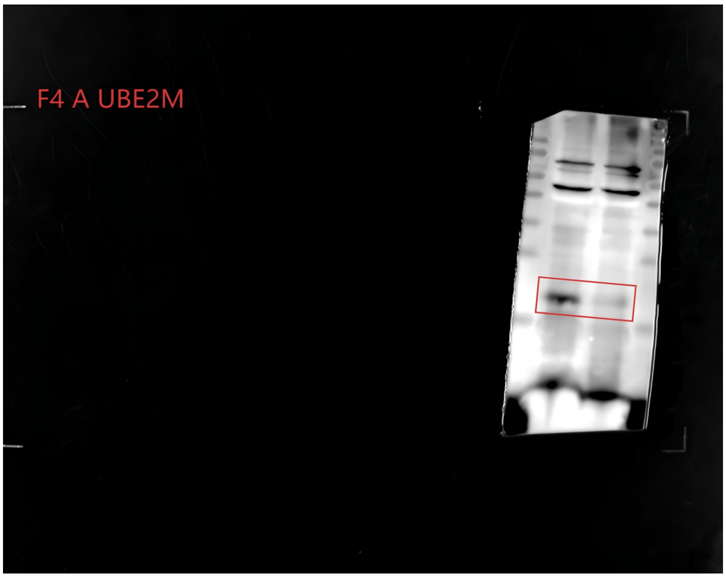
F4 A UBE2M

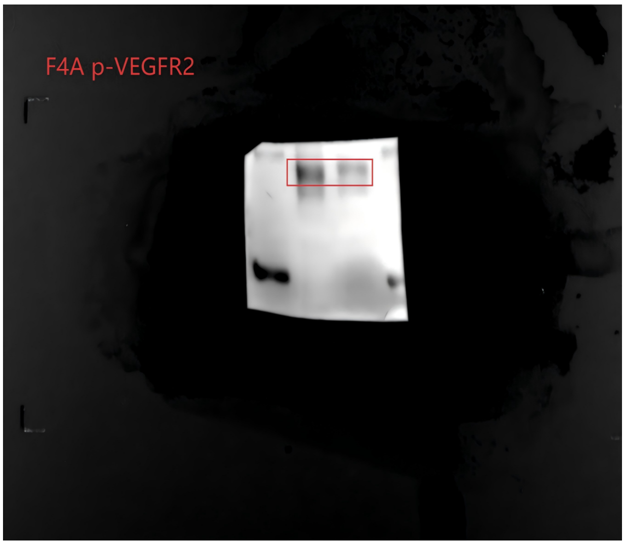
F4A p-VEGFR2

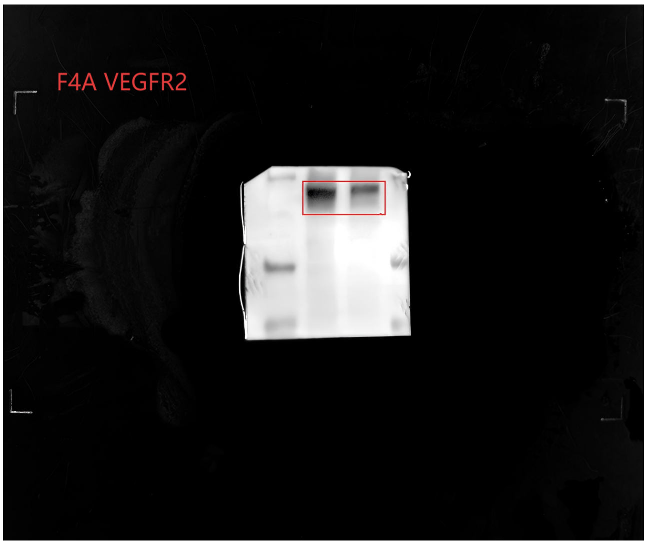
F4A VEGFR2

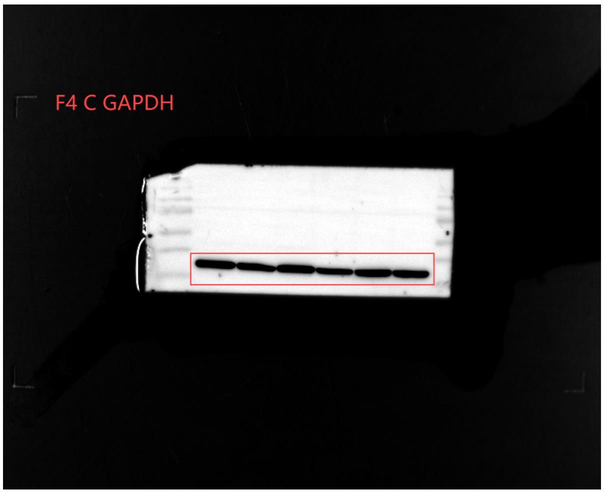
F4 C GAPDH

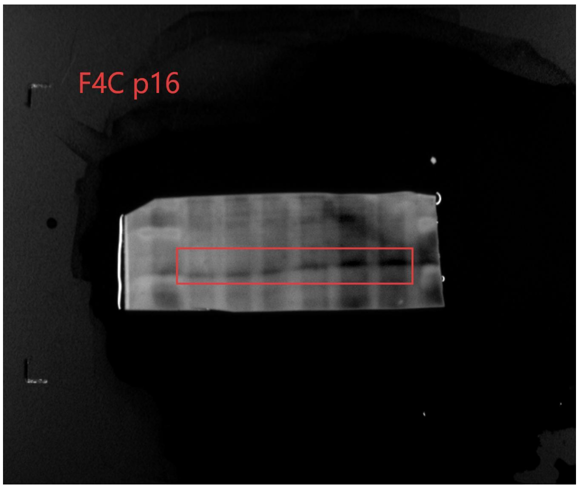
F4C p16

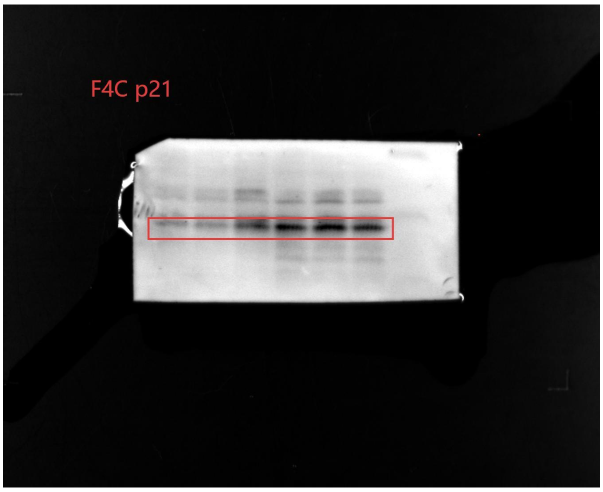
F4C p21

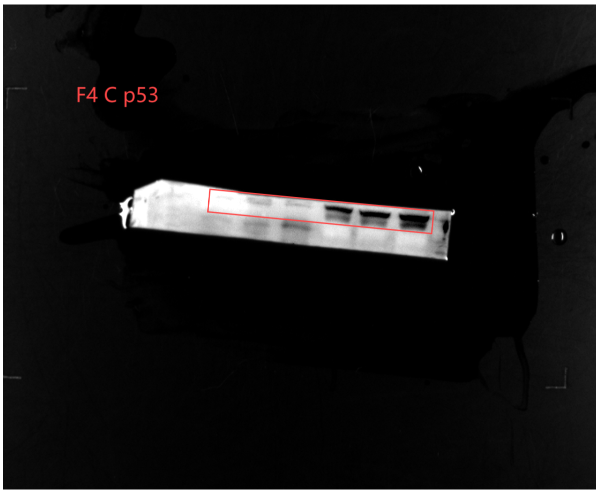
F4 C p53

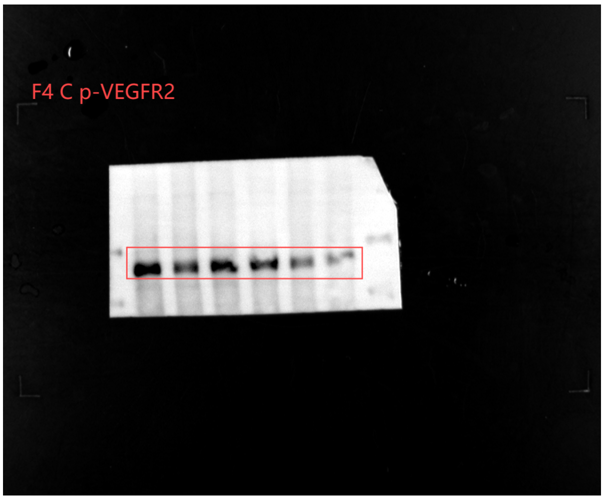
F4 C p-VEGFR2

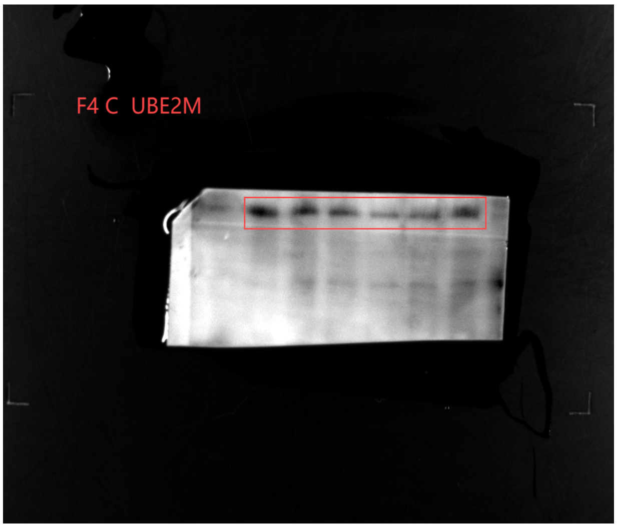
F4 C UBE2M

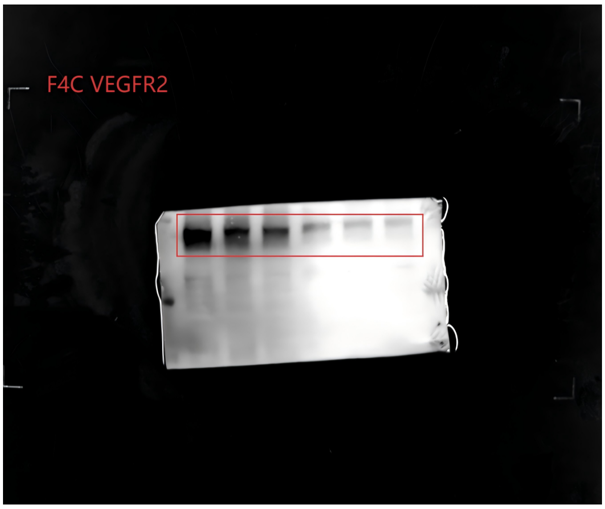
F4C VEGFR2

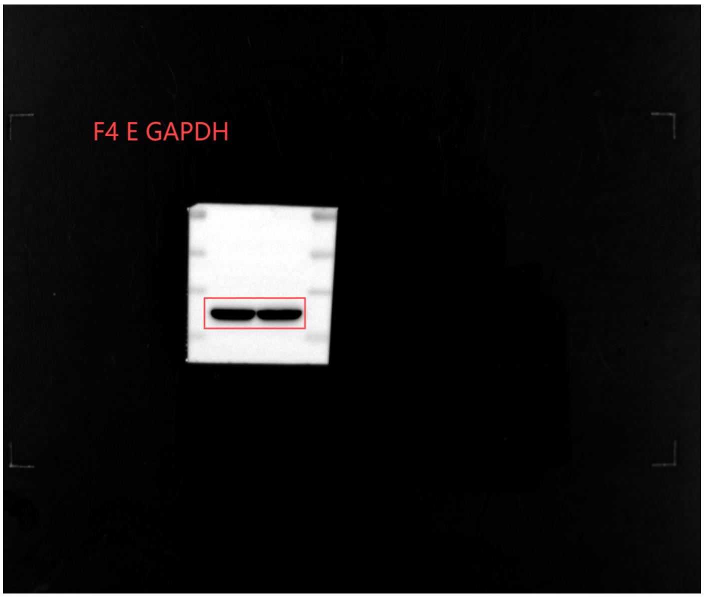
F4 E GAPDH

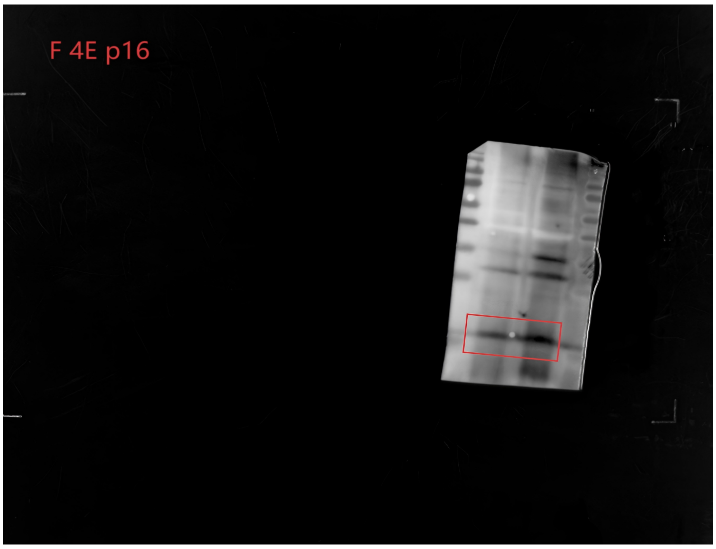
F 4E p16

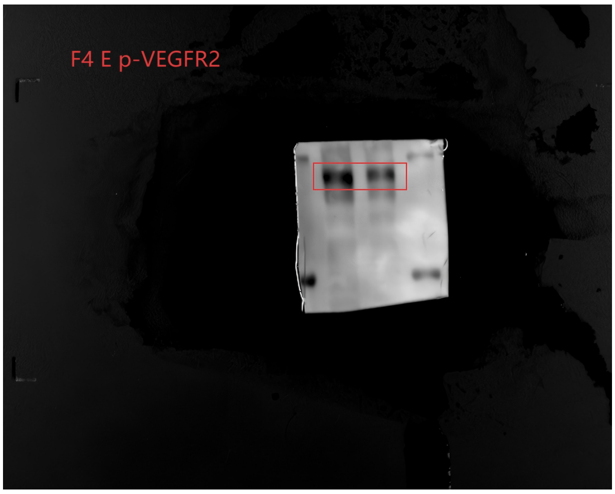
F4 E p-VEGFR2

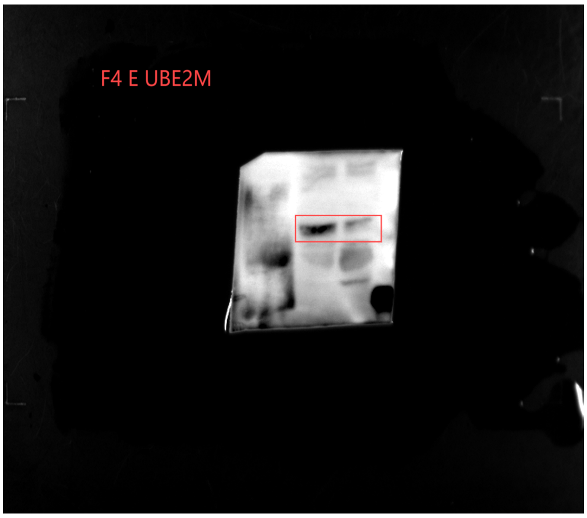
F4 E UBE2M

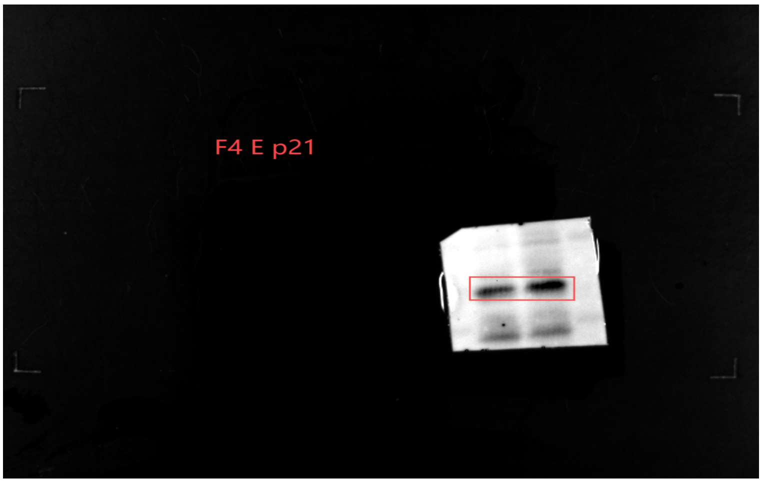
F4 E p21

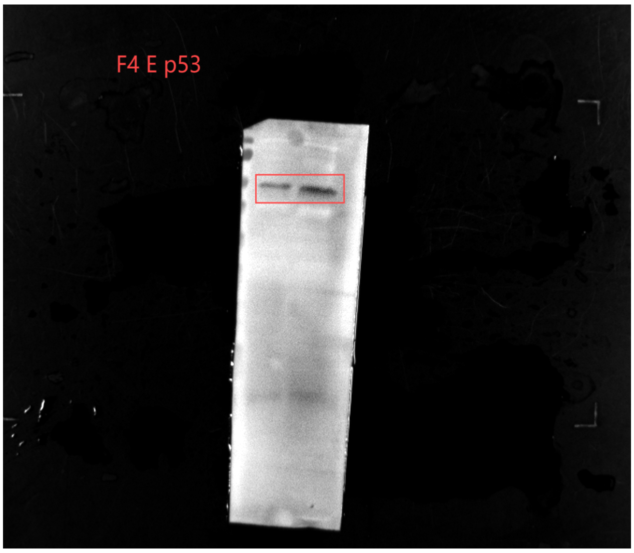
F4 E p53

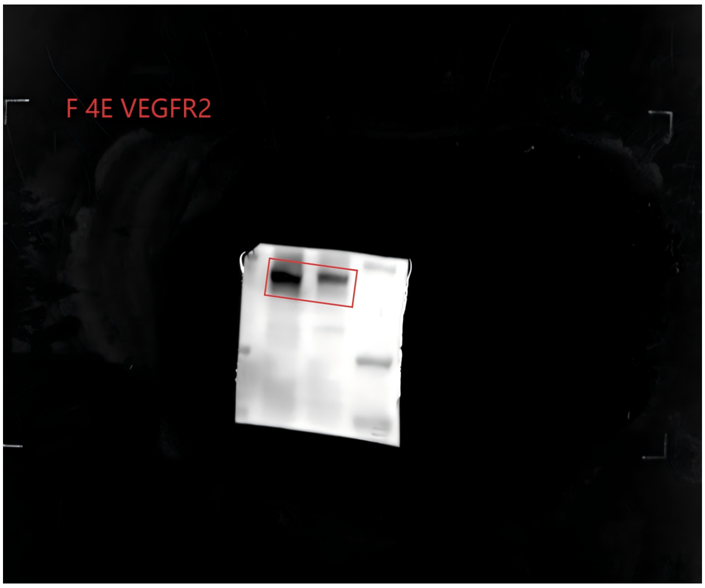
F 4E VEGFR2

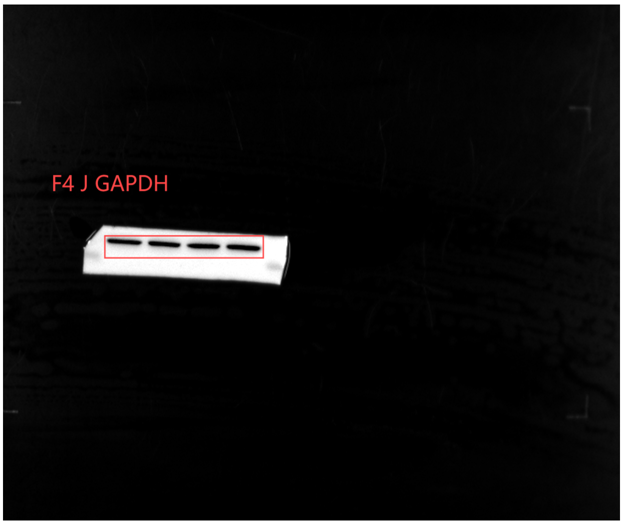
F4 J GAPDH

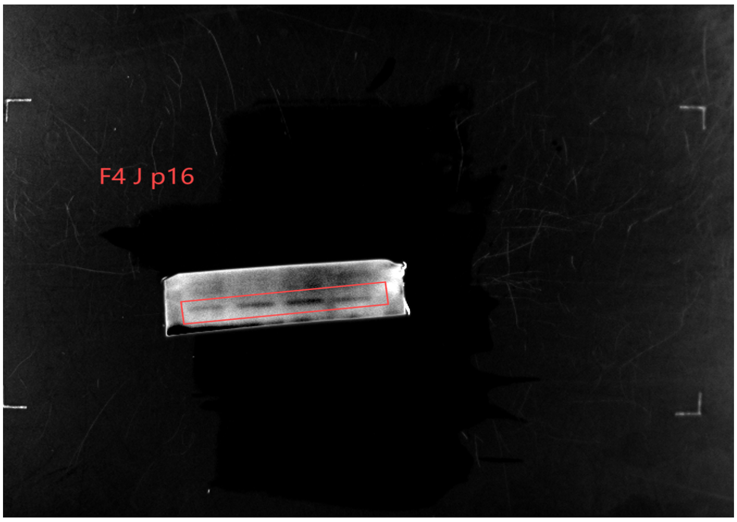
F4 J p16

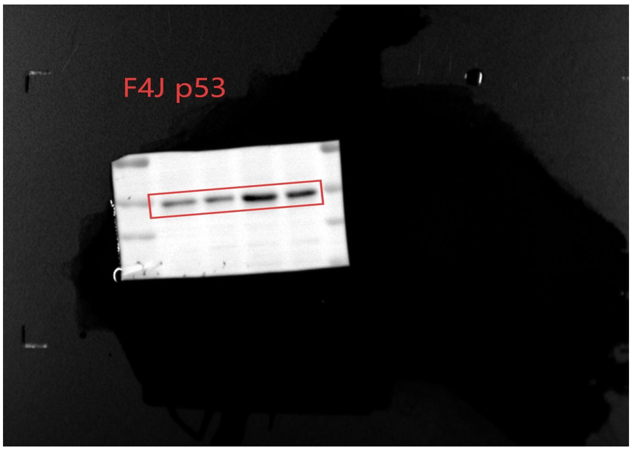
F4J p53

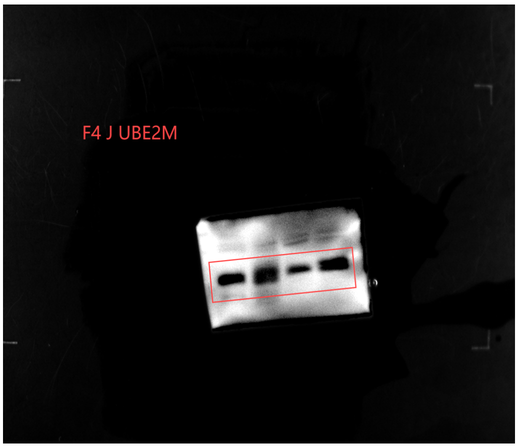
F4 J UBE2M

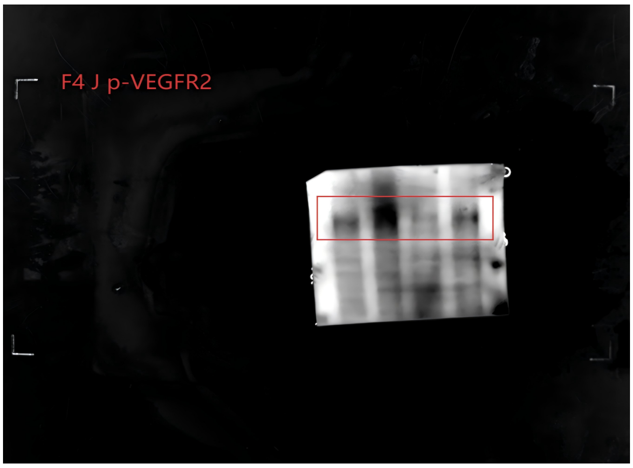
F4 J p-VEGFR2

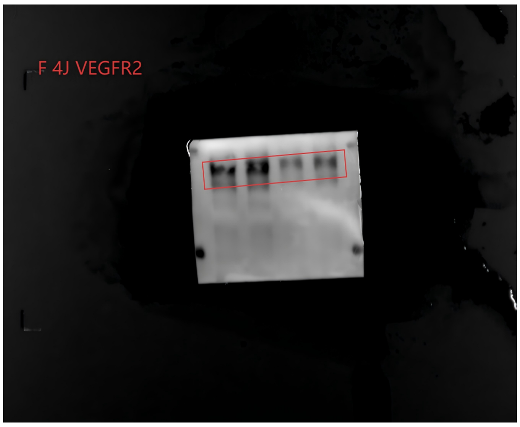
F 4J VEGFR2

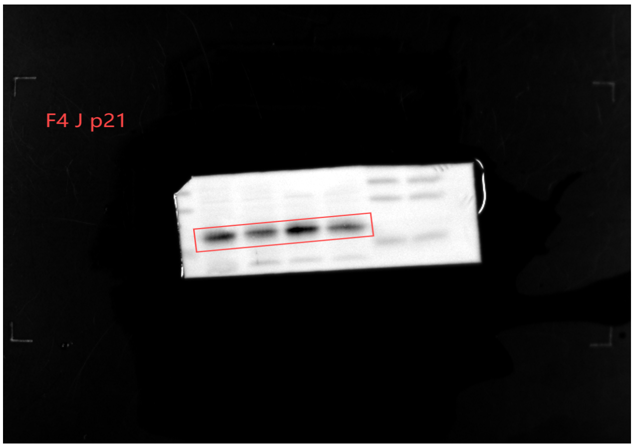
F4 J p21

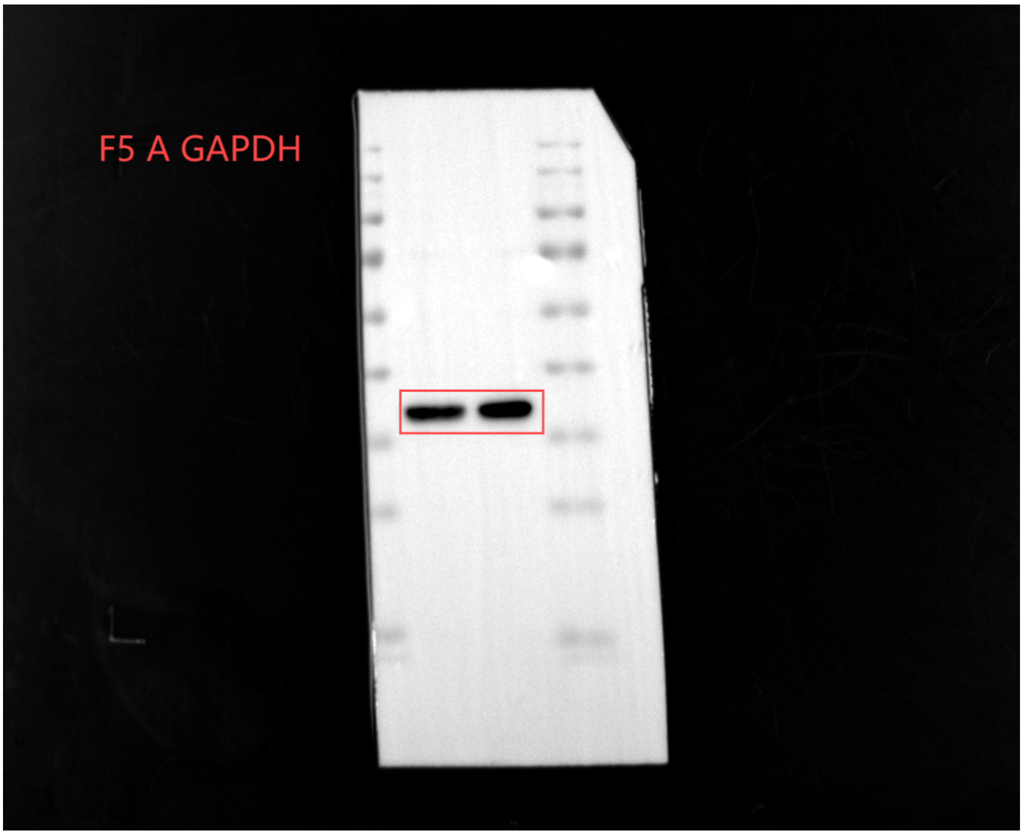
F5 A GAPDH

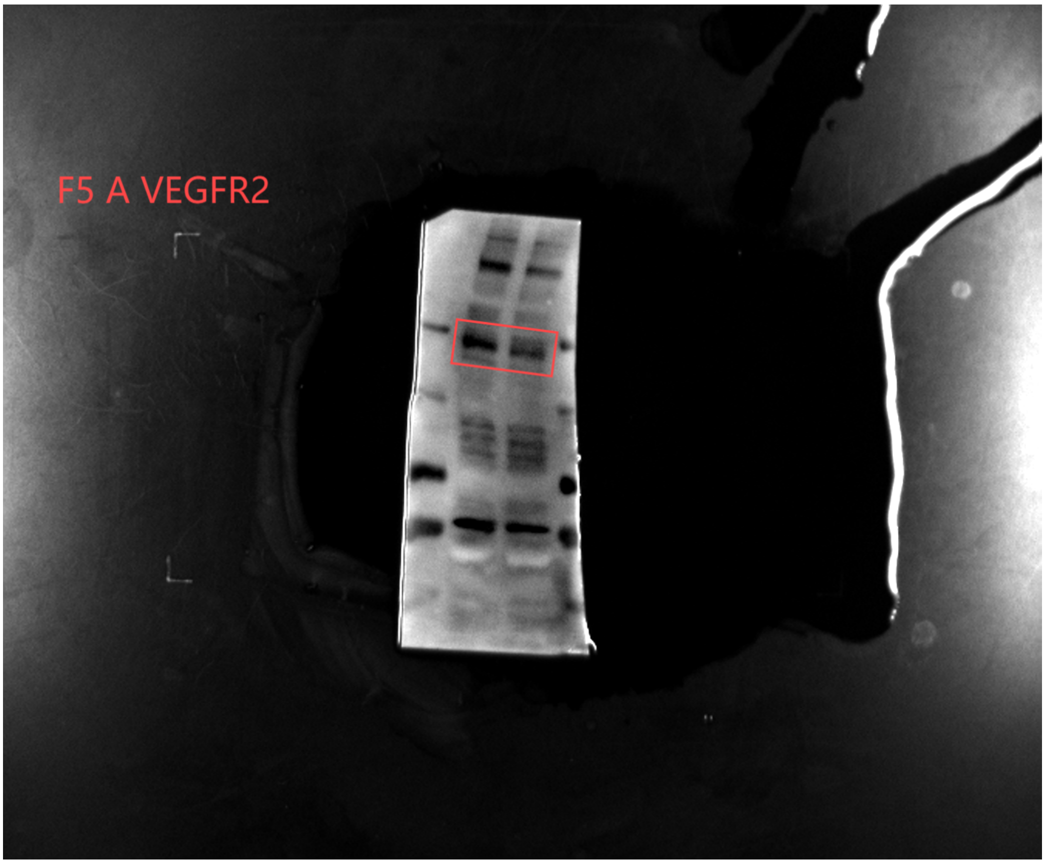
F5 A VEGFR2

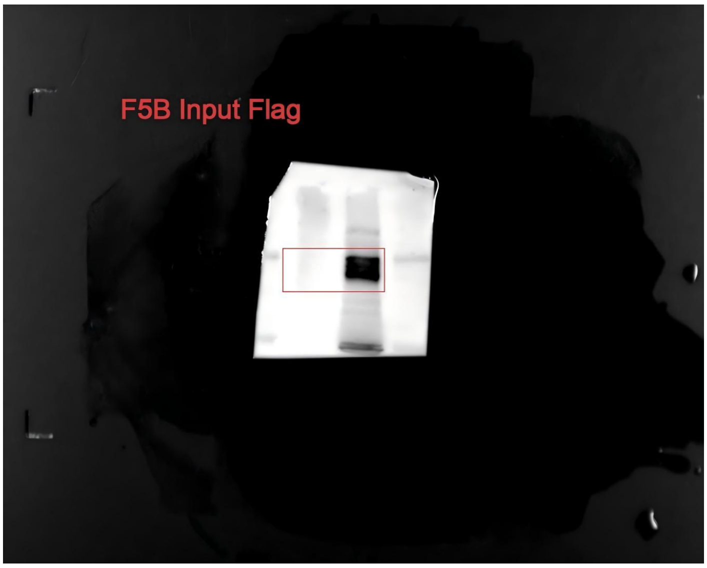
F5B Input Flag

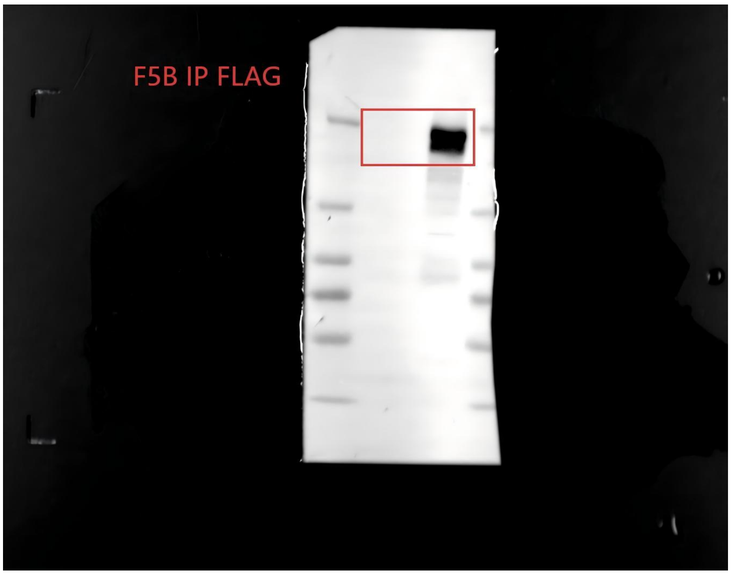
F5B IP FLAG

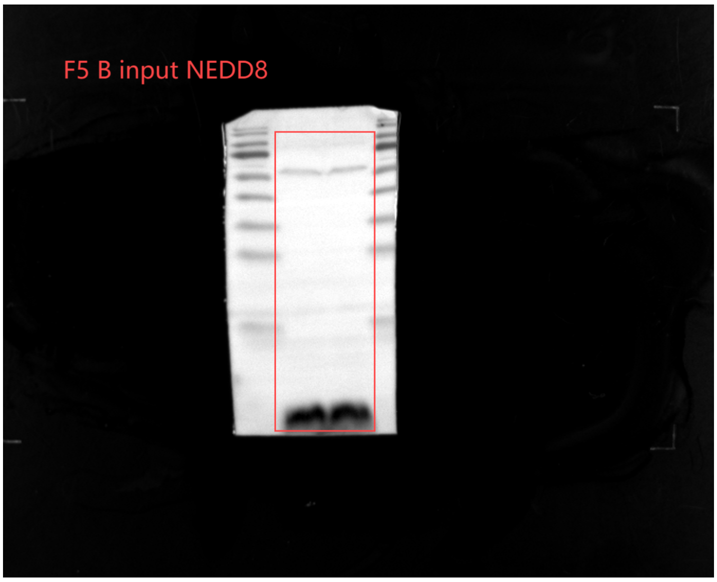
F5 B input NEDD8

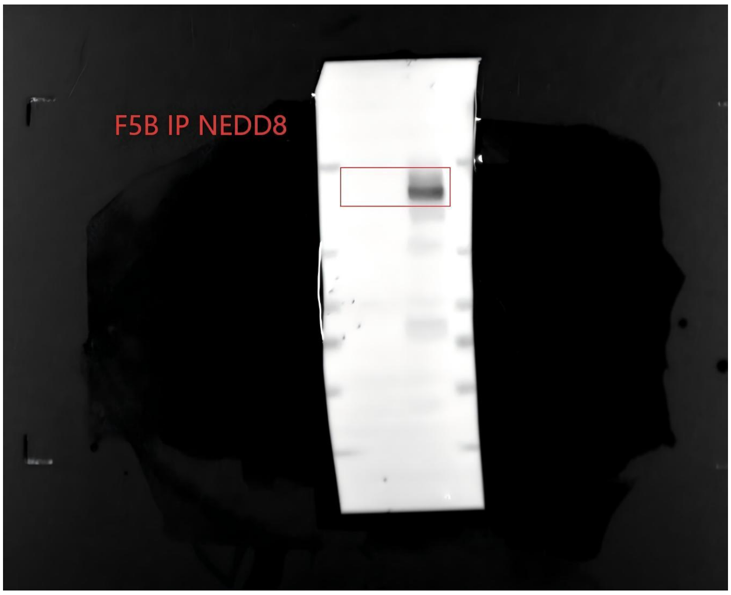
F5B IP NEDD8

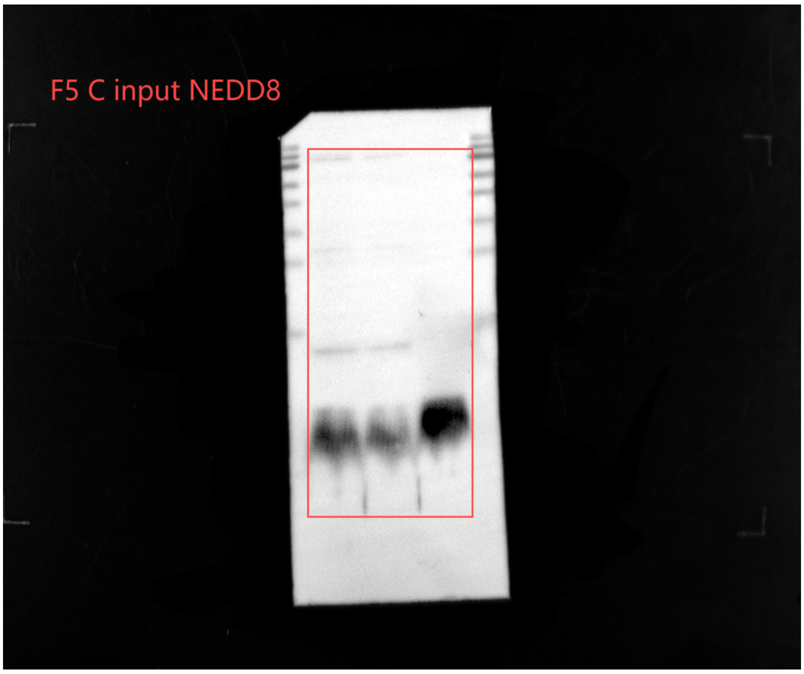
F5 C input NEDD8

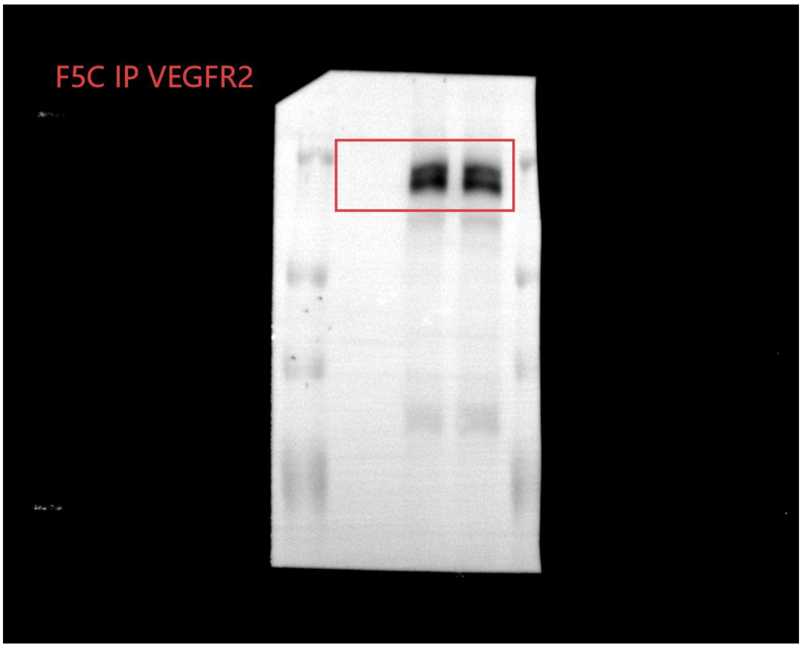
F5C IP VEGFR2

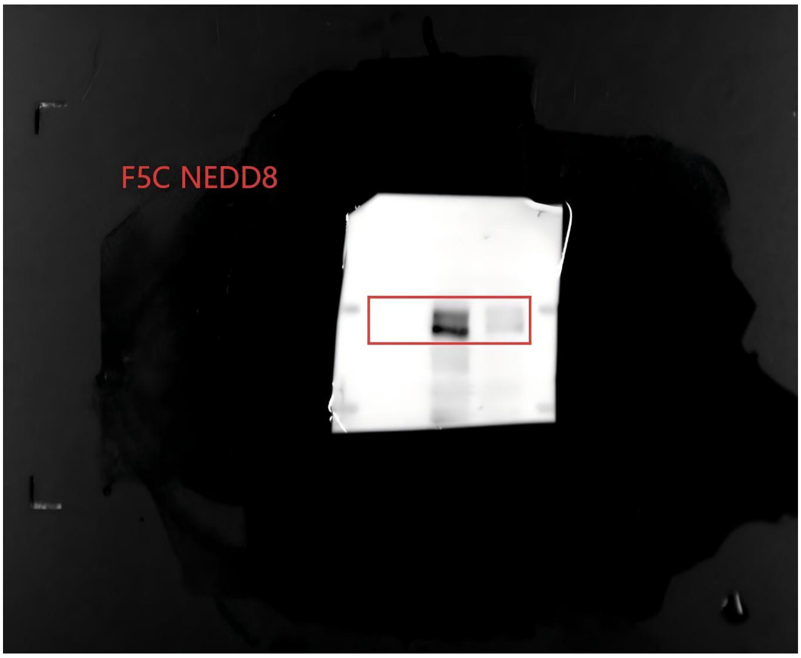
F5C NEDD8

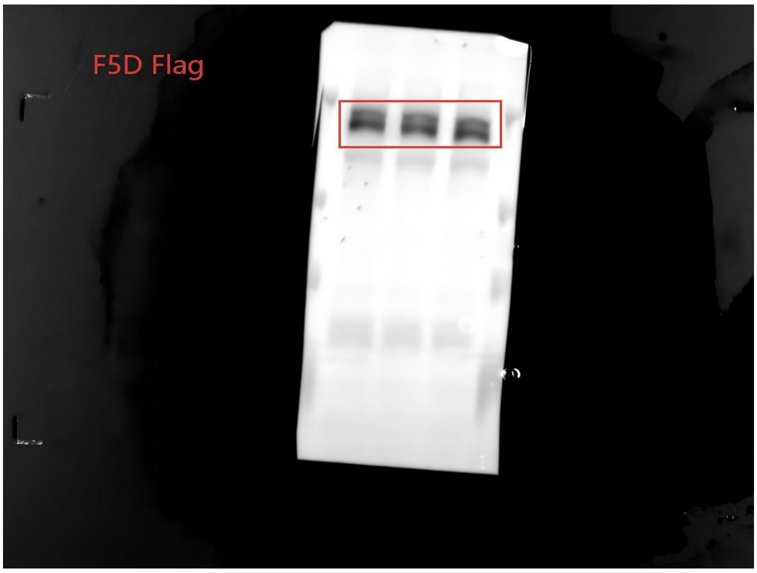
F5D Flag

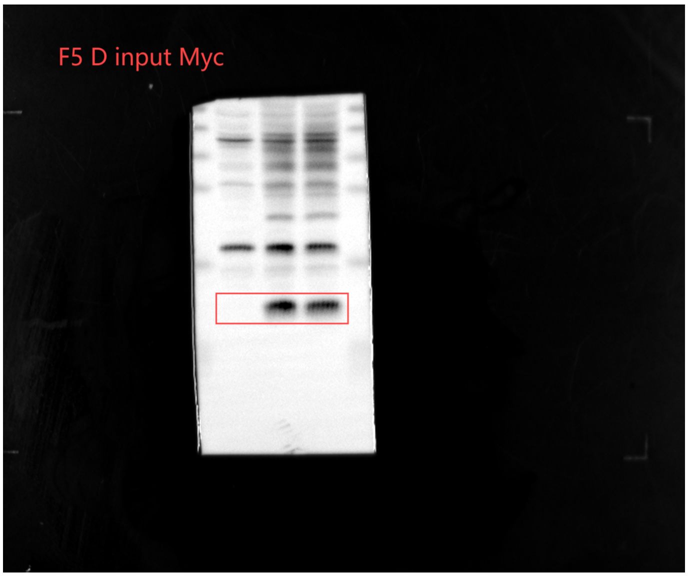
F5 D input Myc

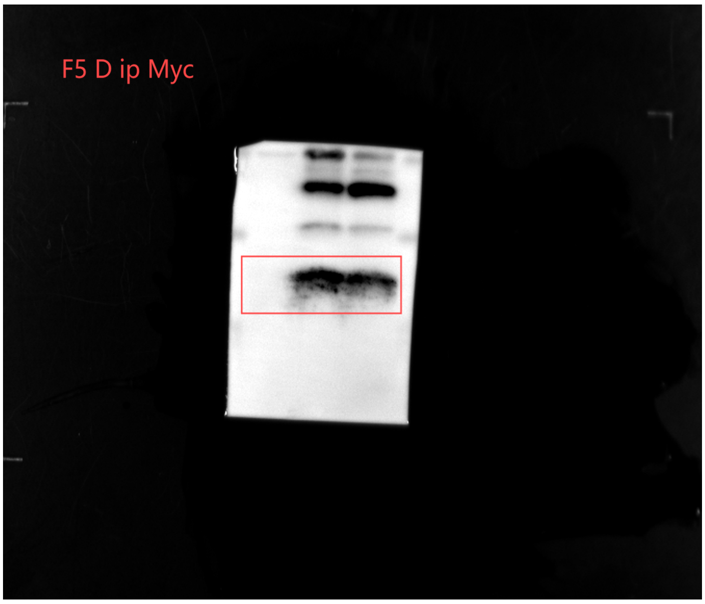
F5 D ip Myc

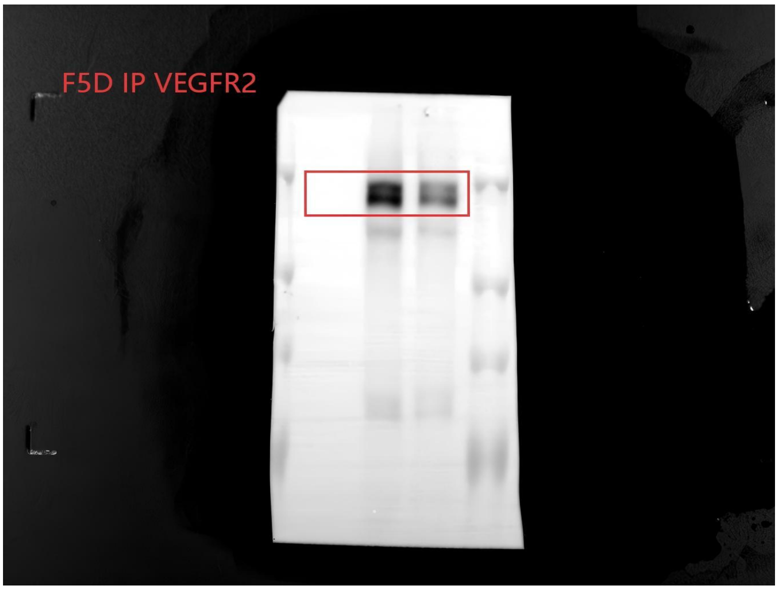
F5D IP VEGFR2

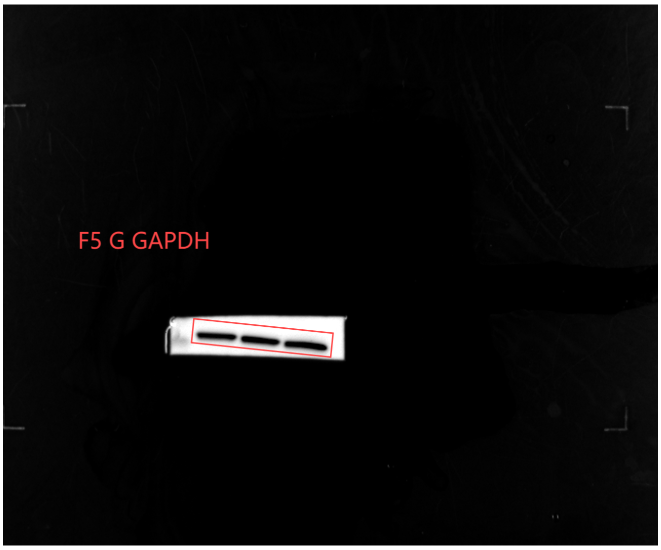
F5 G GAPDH

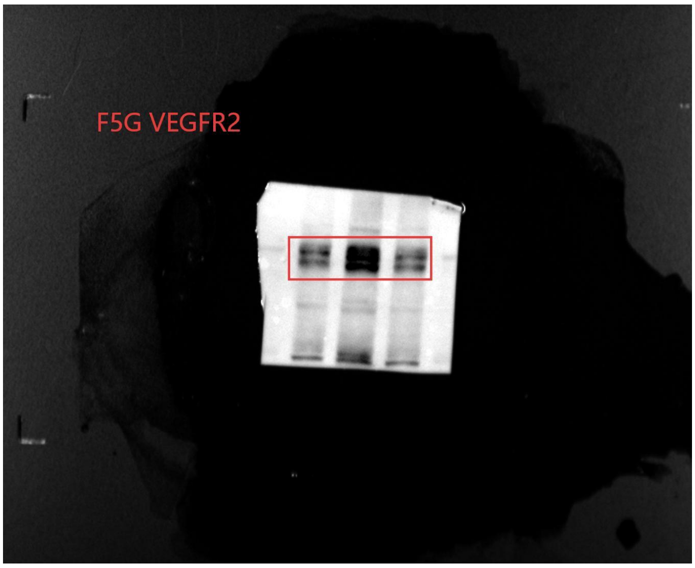
F5G VEGFR2

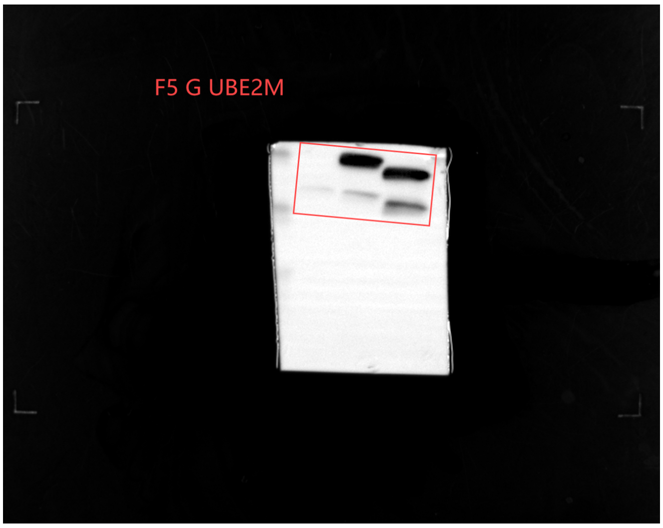
F5 G UBE2M

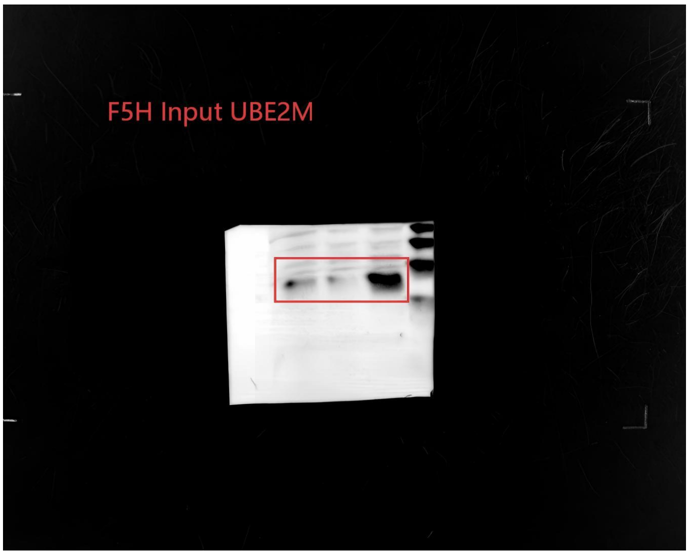
F5H Input UBE2M

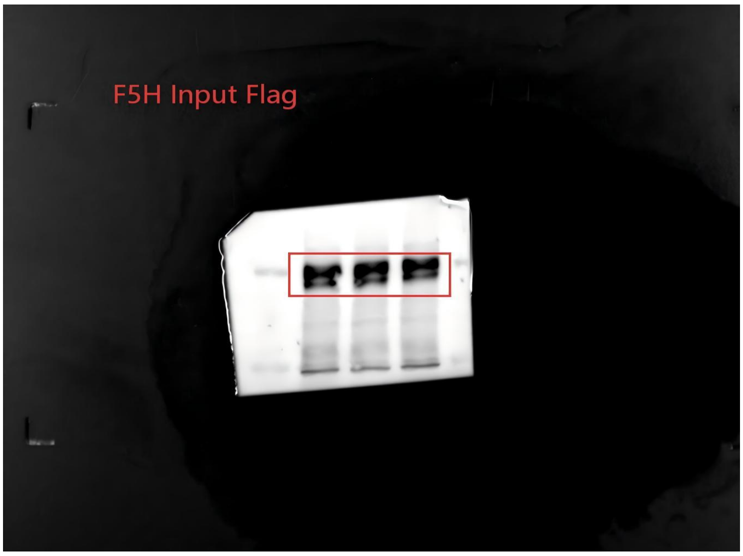
F5H Input Flag

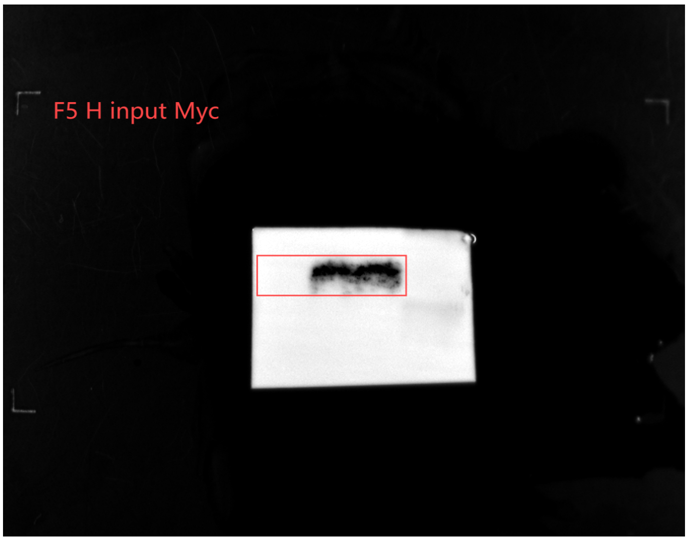
F5 H input Myc

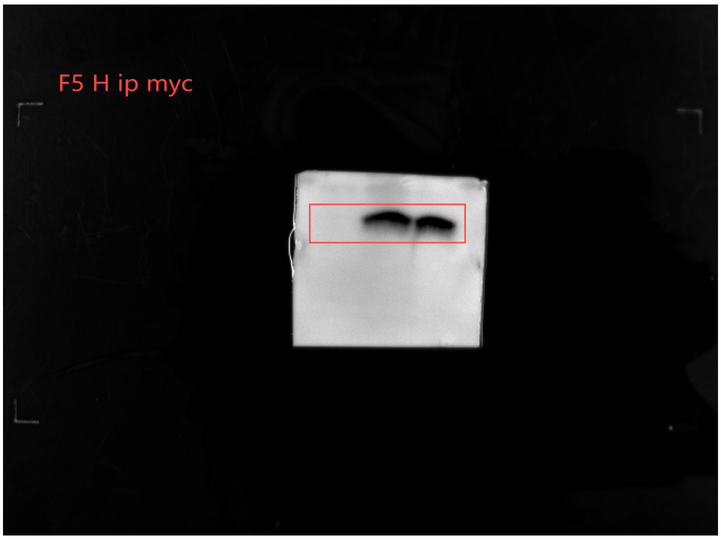
F5 H ip myc

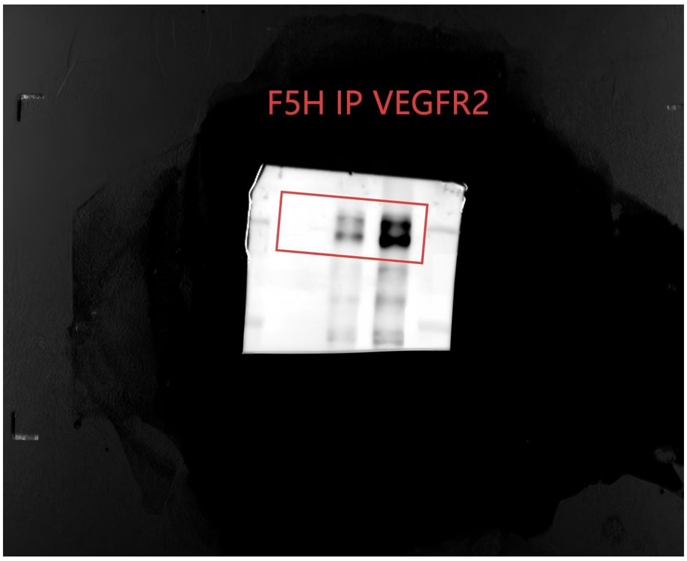
F5H IP VEGFR2

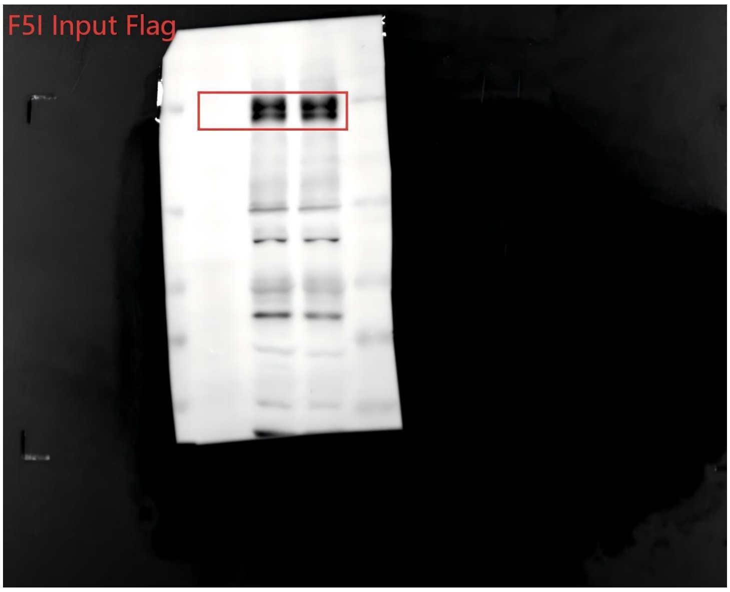
F5I Input Flag

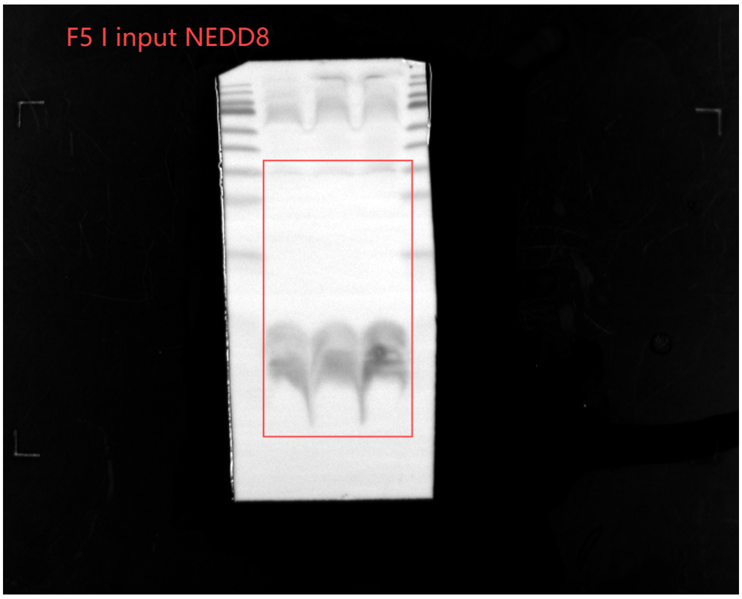
F5 I input NEDD8

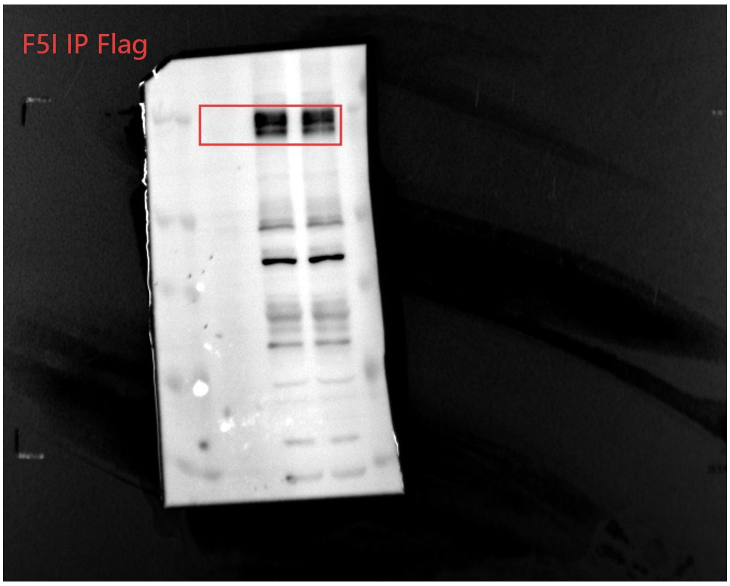
F5I IP Flag

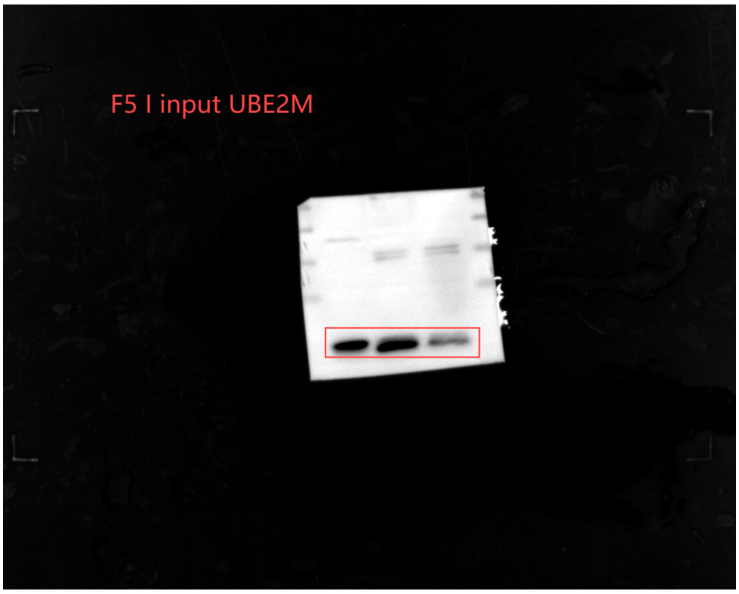
F5 I input UBE2M

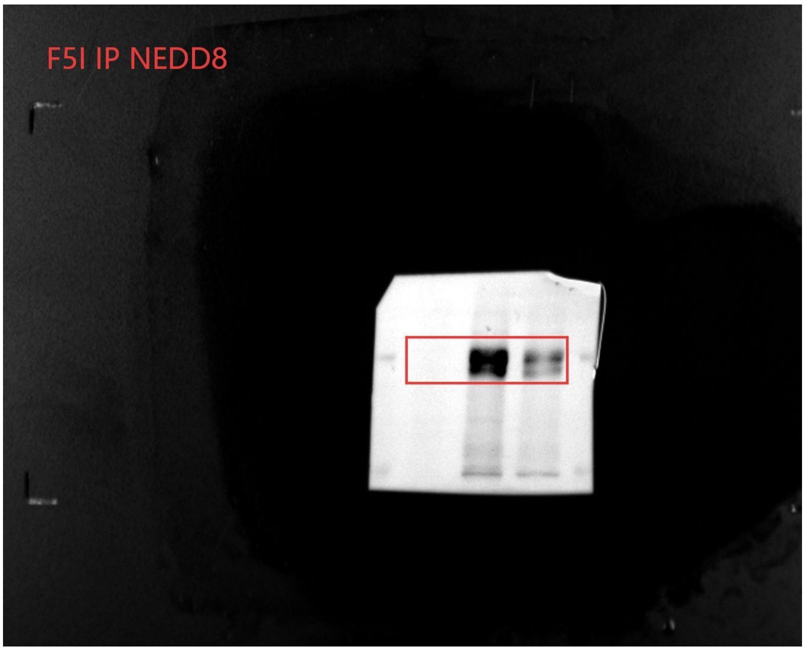
F5I IP NEDD8

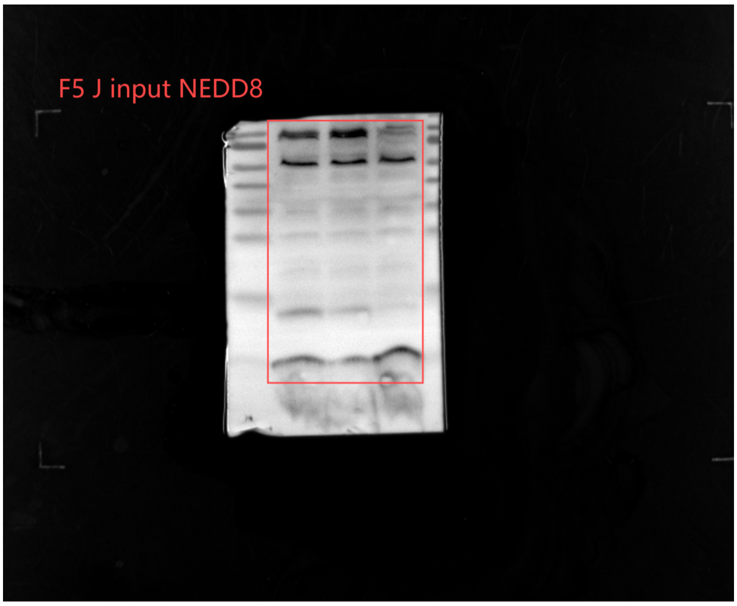
F5 J input NEDD8

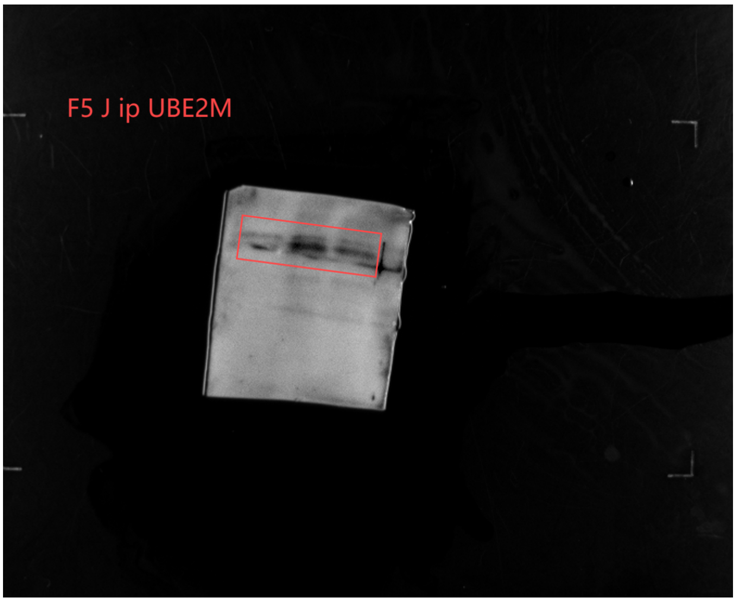
F5 J ip UBE2M

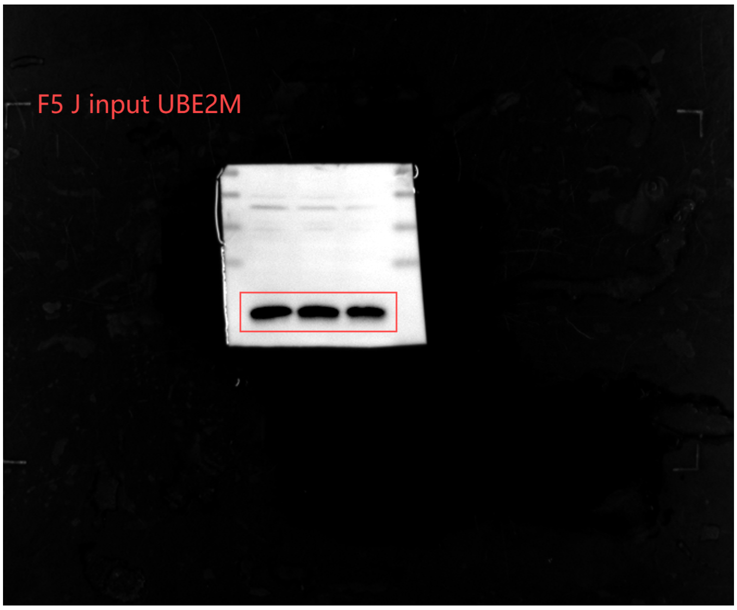
F5 J input UBE2M

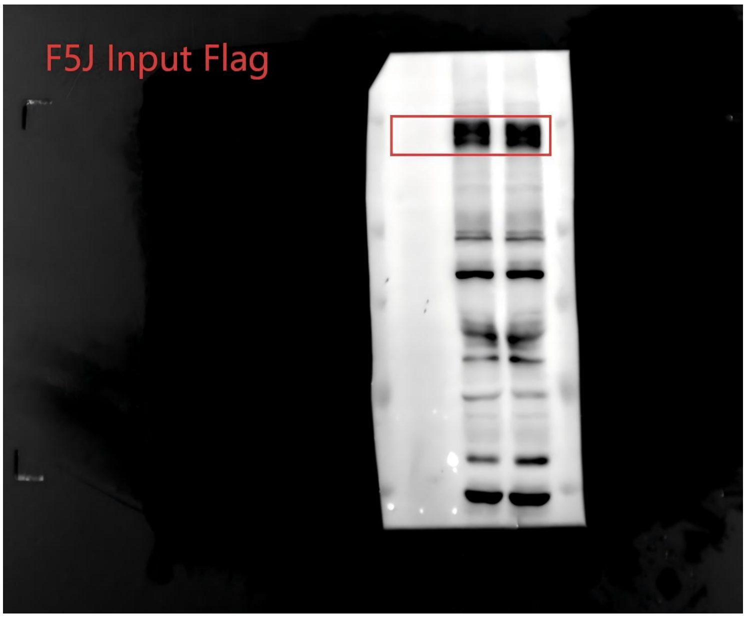
F5J Input Flag

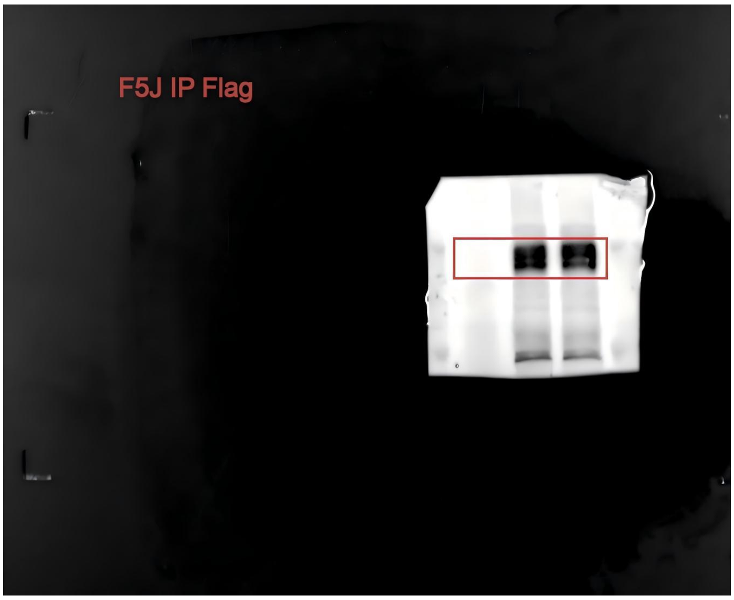
F5J IP Flag

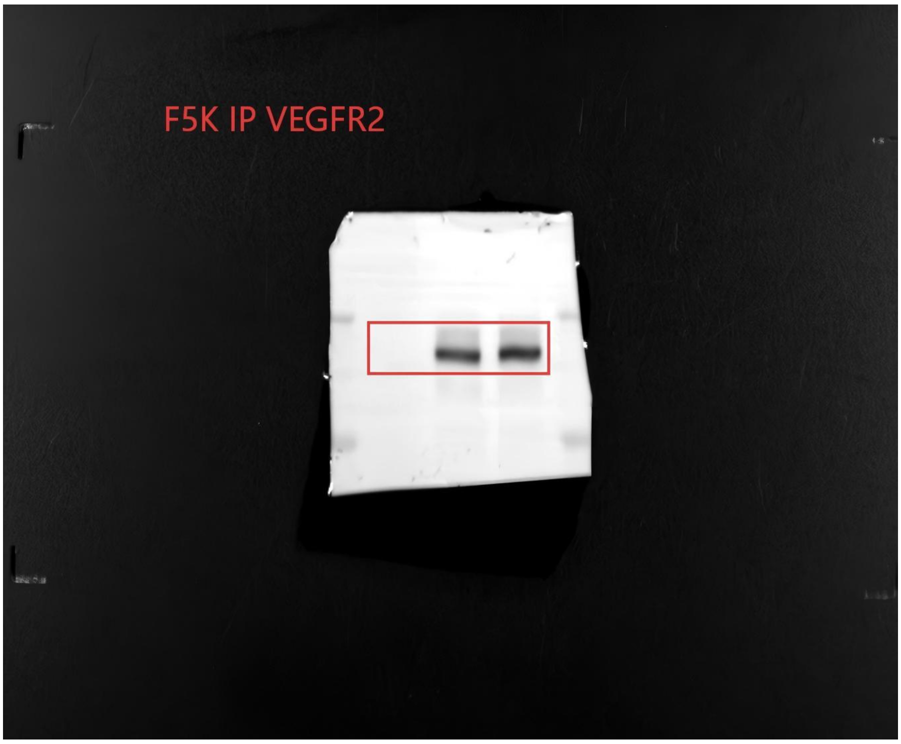
F5K IP VEGFR2

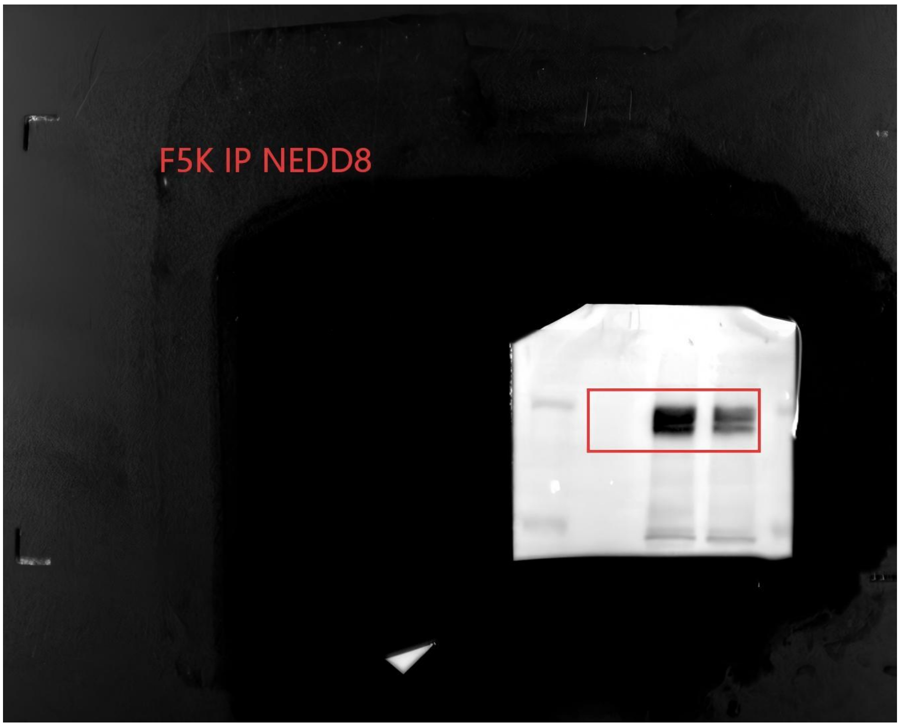
F5K IP NEDD8

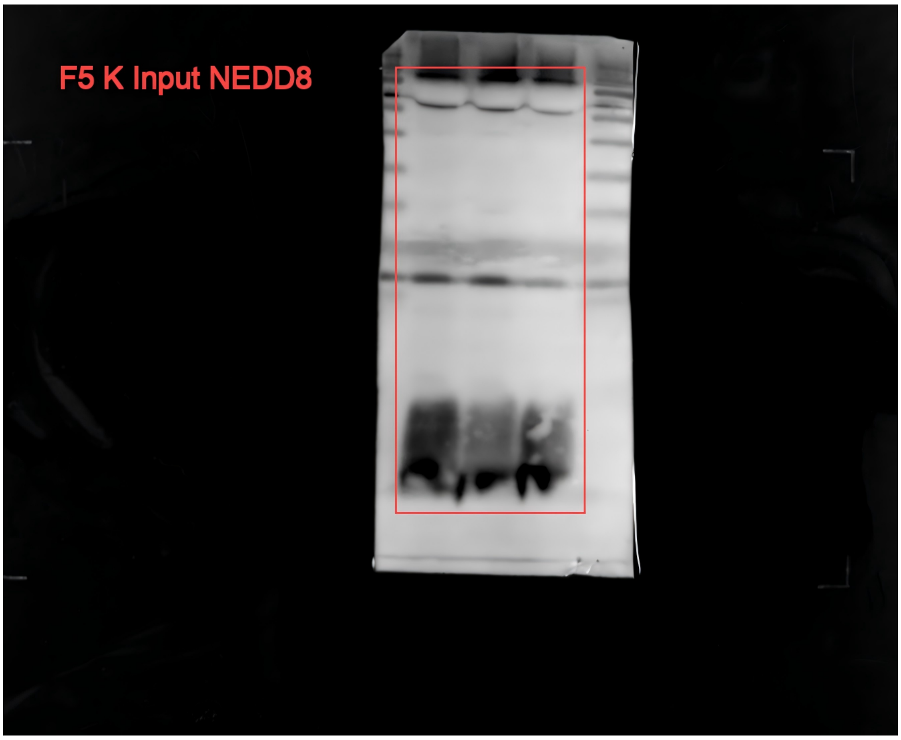
F5 K Input NEDD8

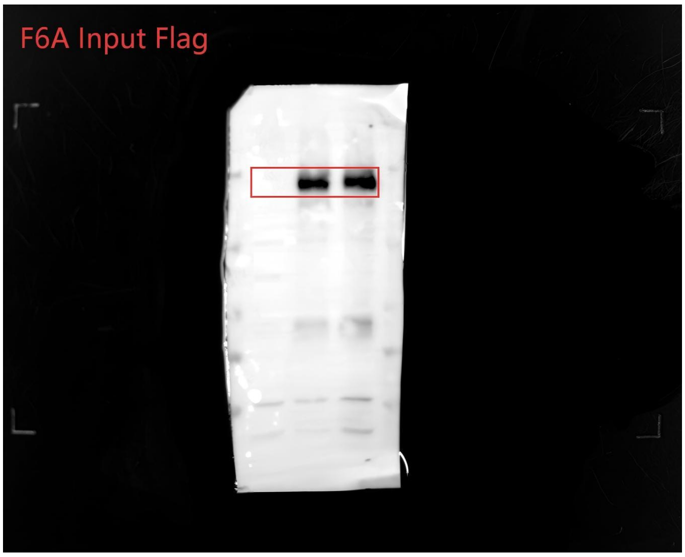
F6A Input Flag

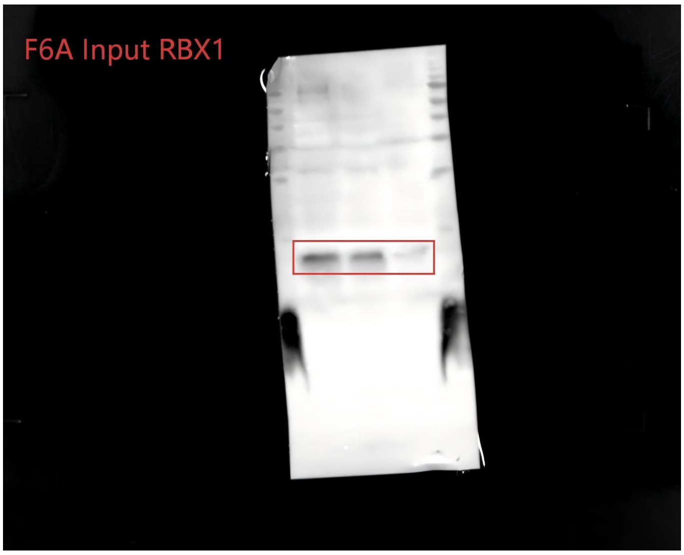
F6A Input RBX1

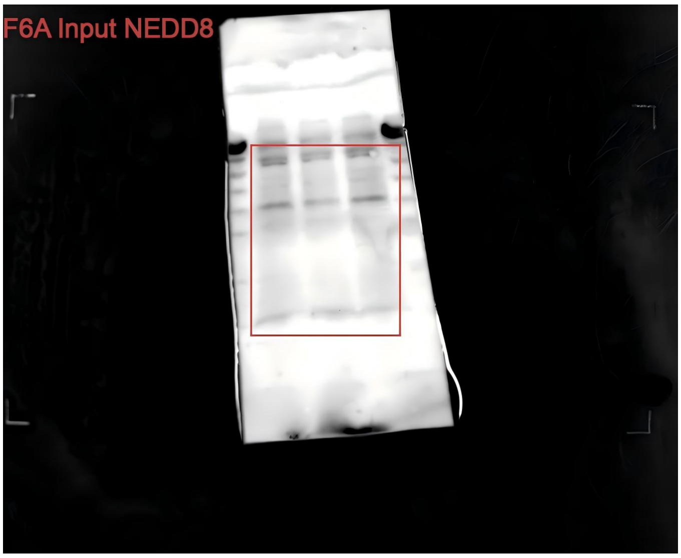
F6A Input NEDD8

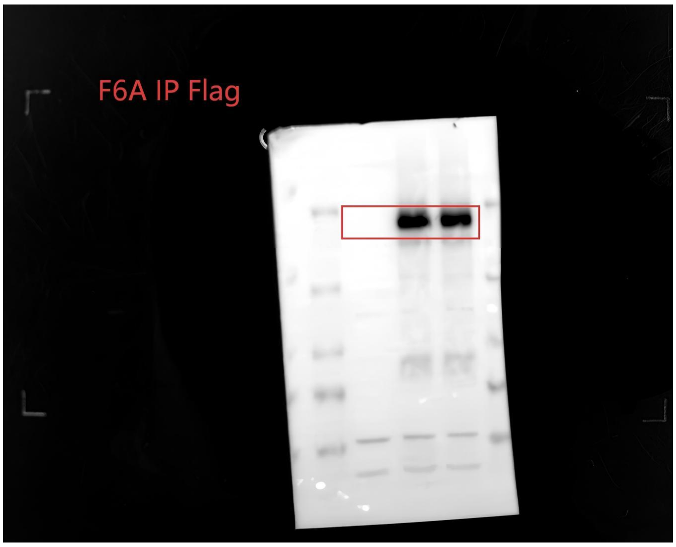
F6A IP Flag

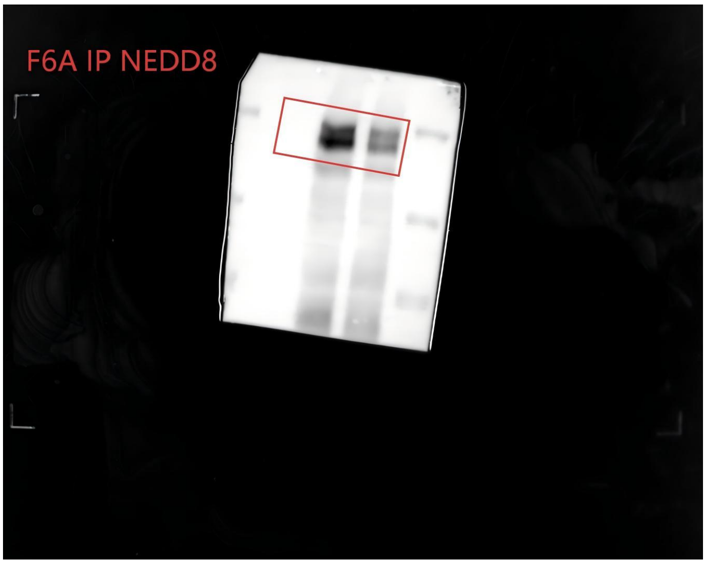
F6A IP NEDD8

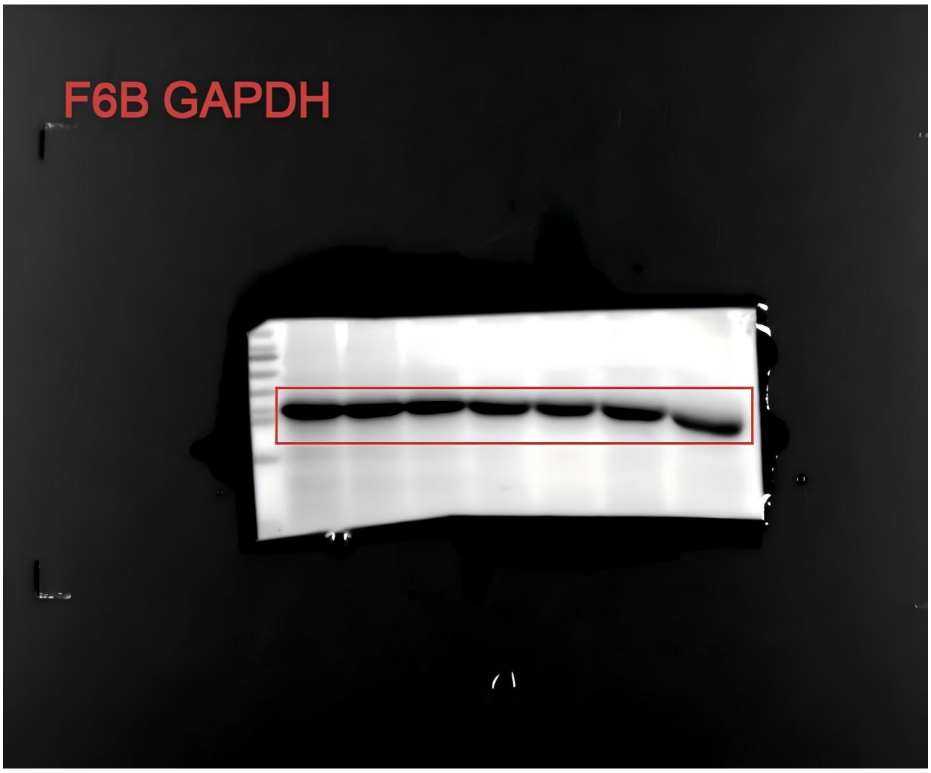
F6B GAPDH

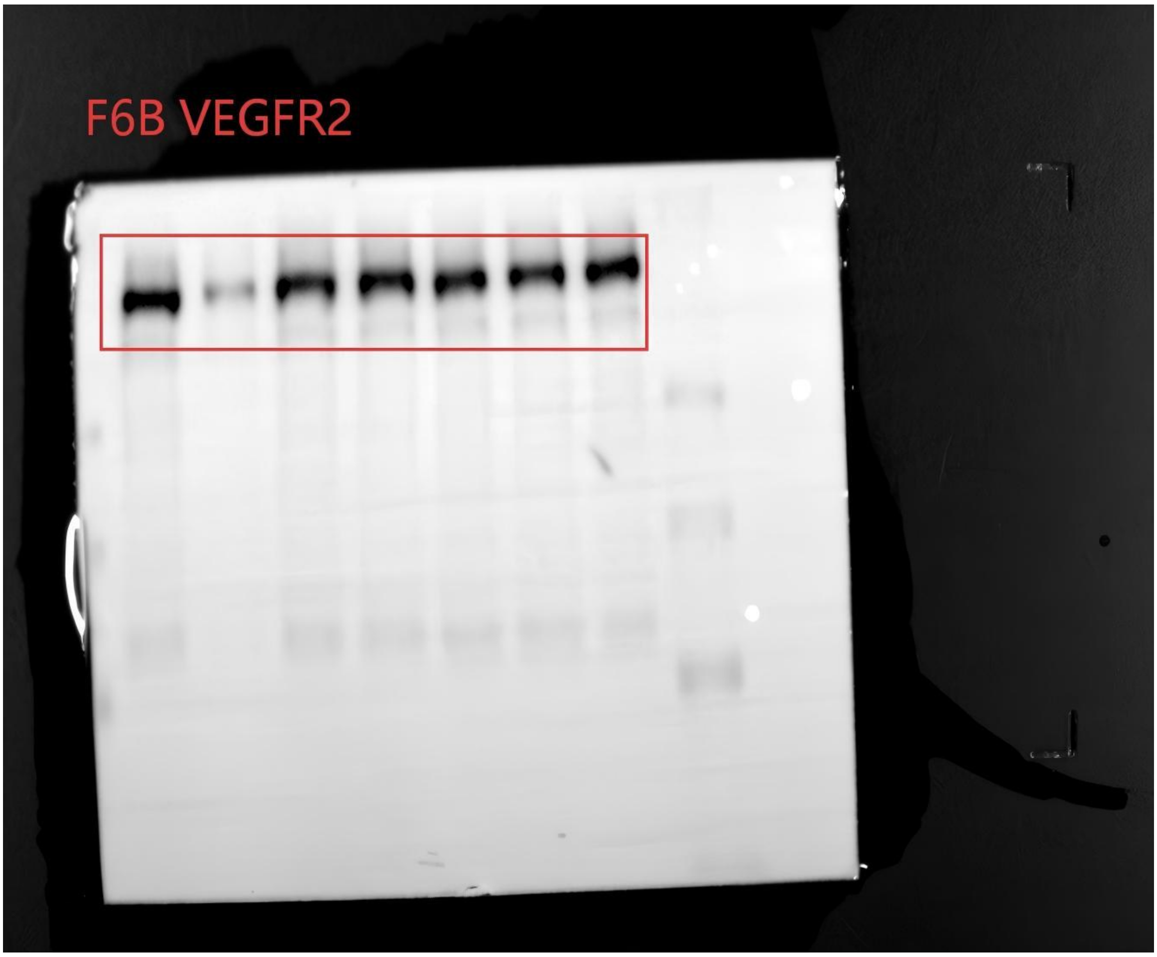
F6B VEGFR2

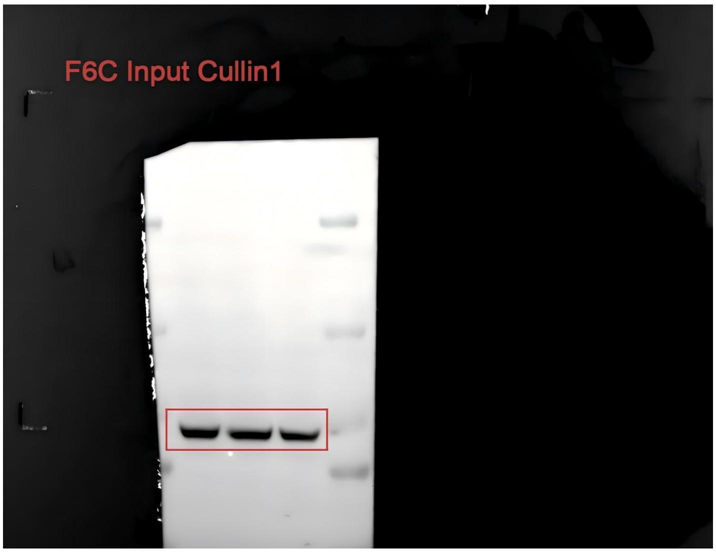
F6C Input Cullin1

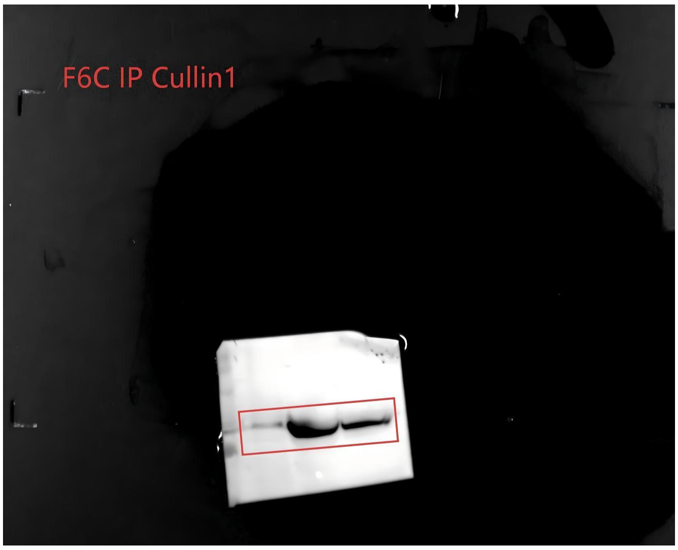
F6C IP Cullin1

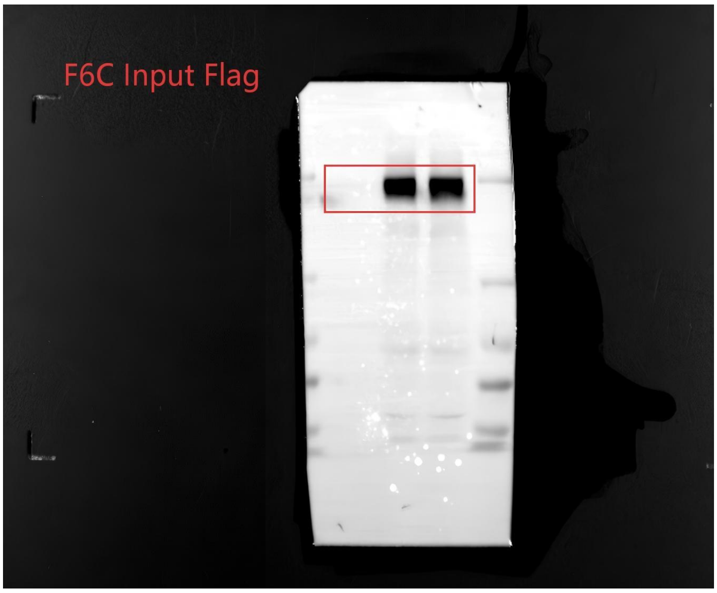
F6C Input Flag

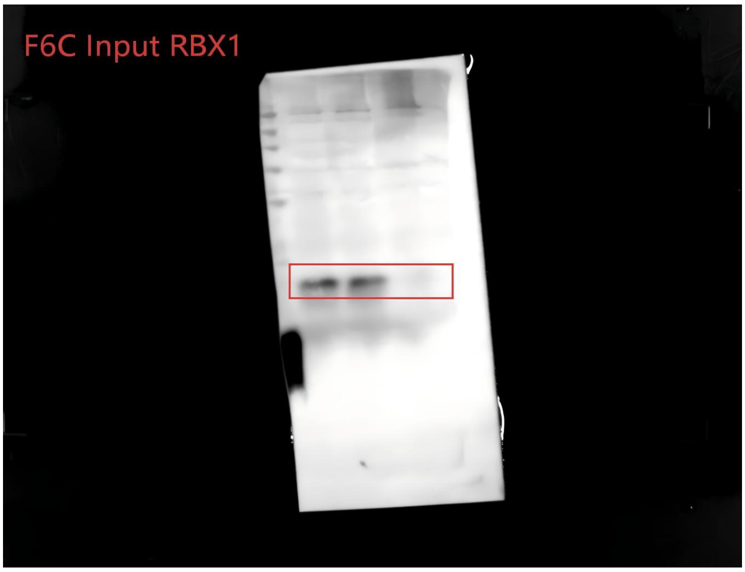
F6C Input RBX1

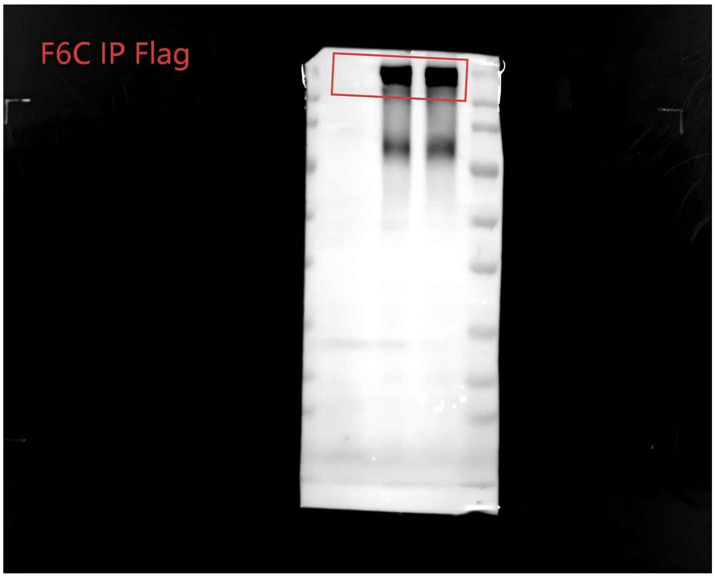
F6C IP Flag

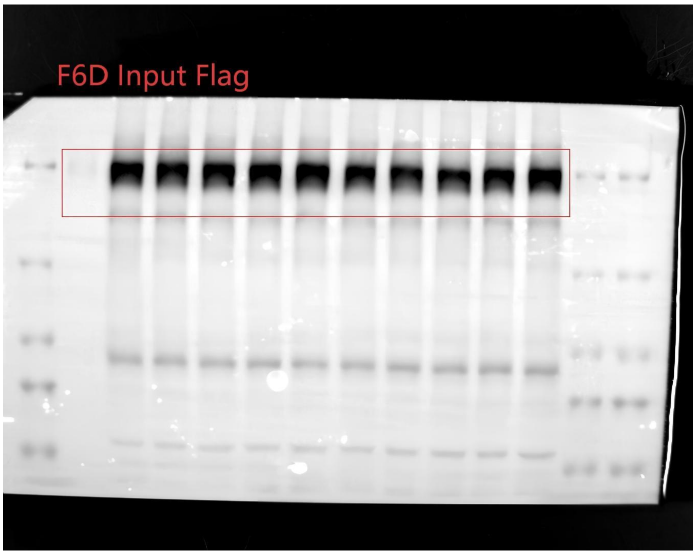
F6D Input Flag

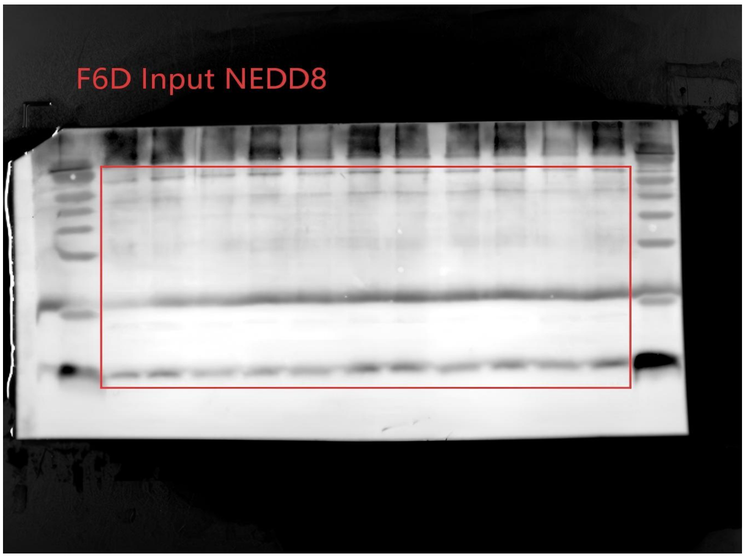
F6D Input NEDD8

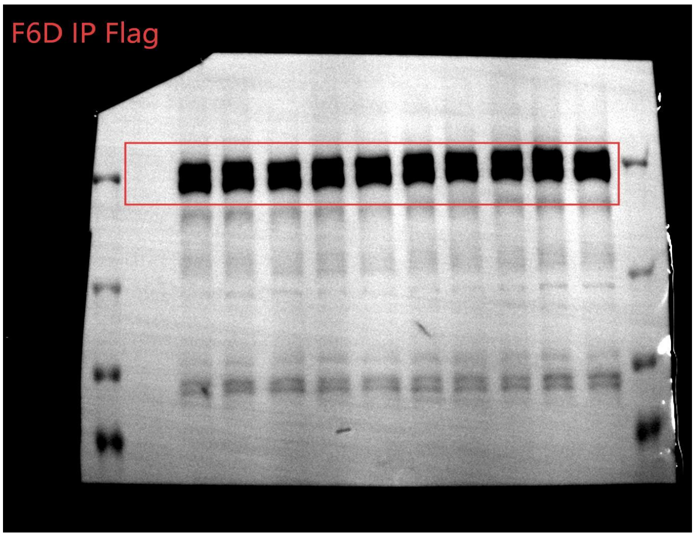
F6D IP Flag

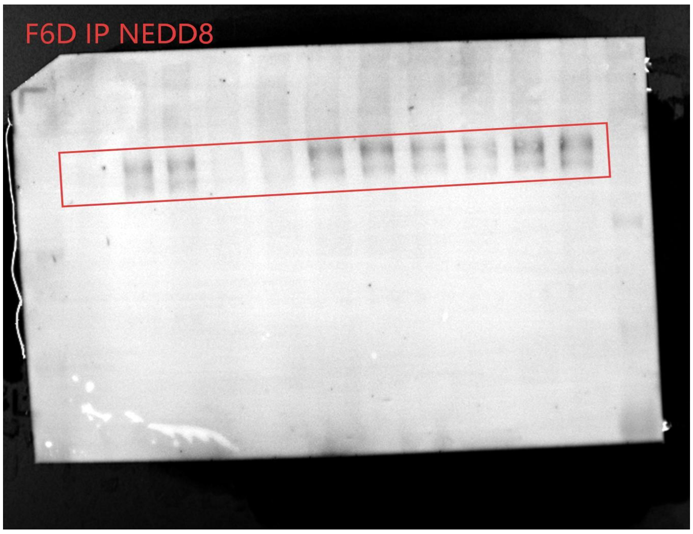
F6D IP NEDD8

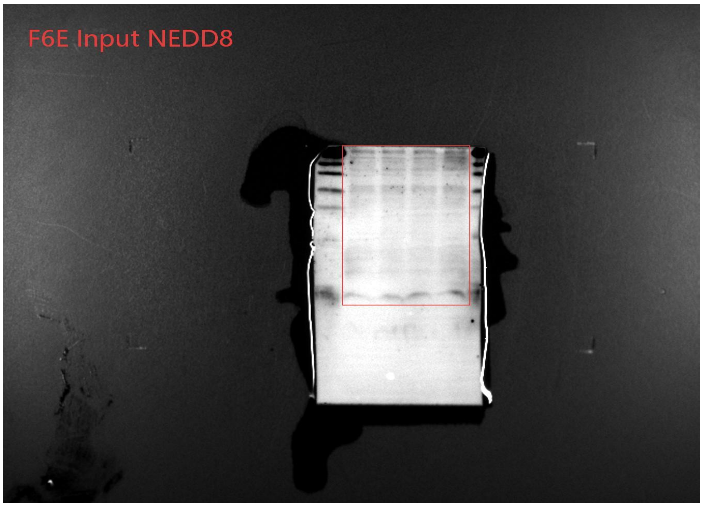
F6E Input NEDD8

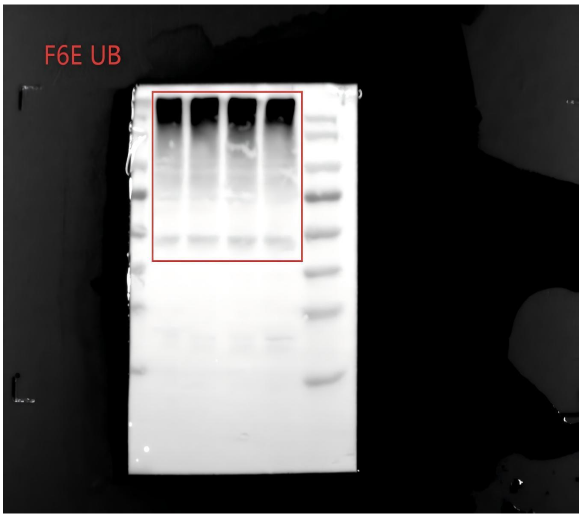
F6E UB

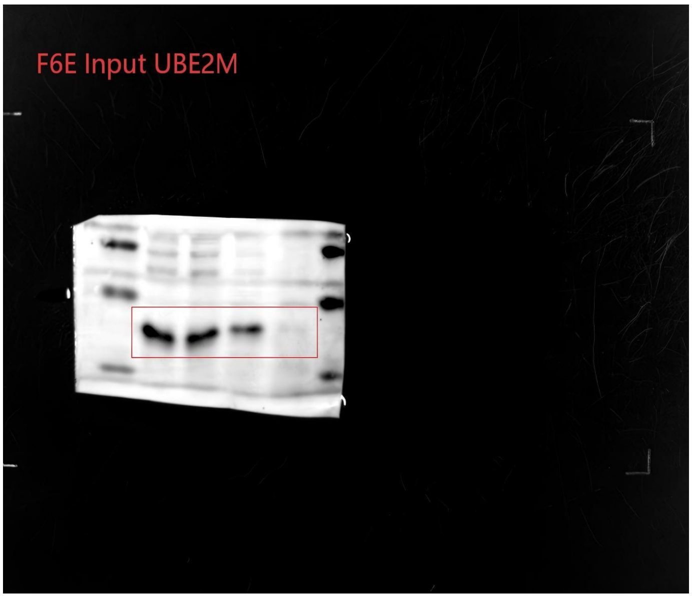
F6E Input UBE2M

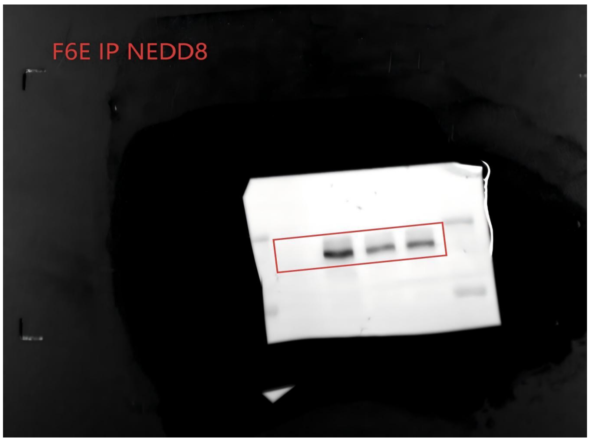
F6E IP NEDD8

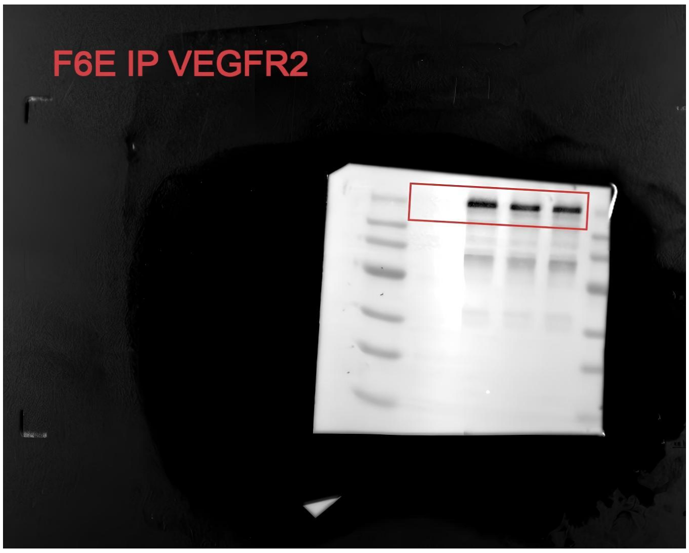
F6E IP VEGFR2

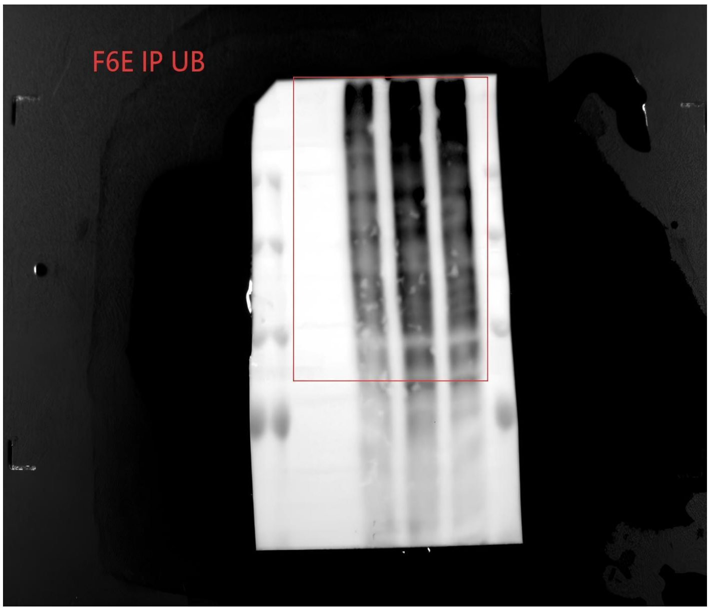
F6E IP UB

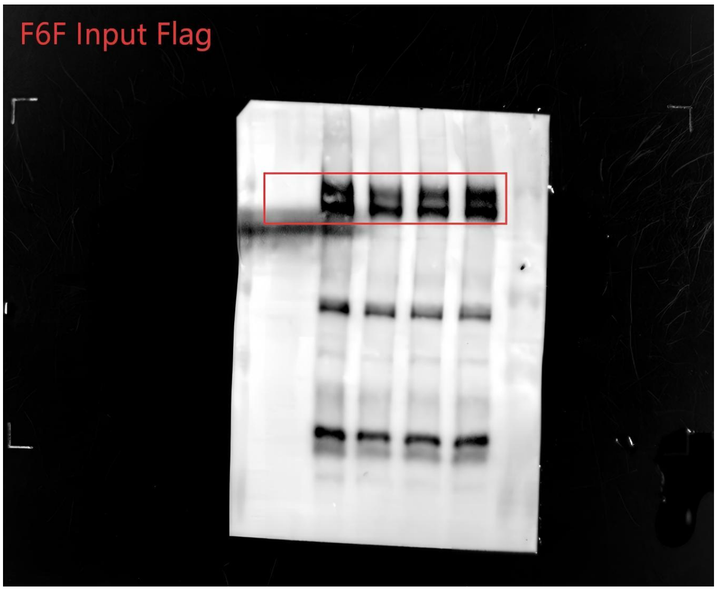
F6F Input Flag

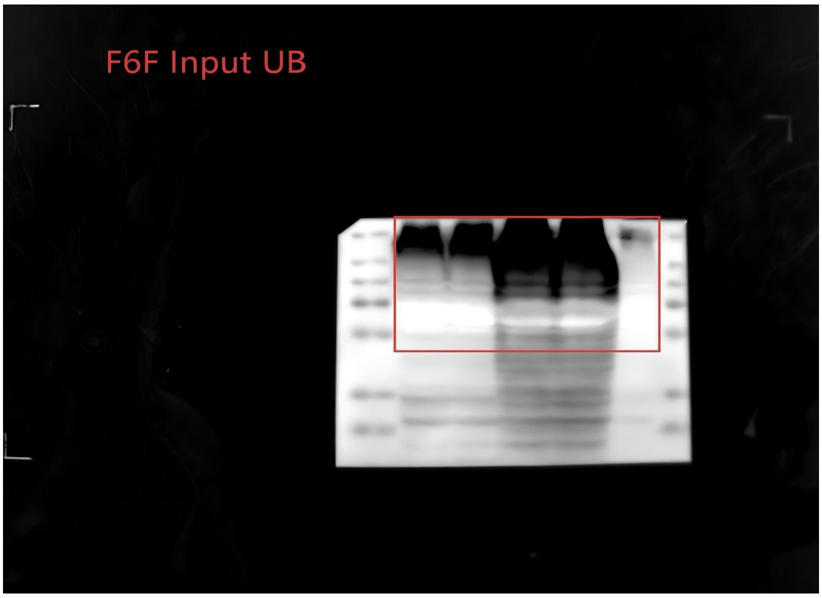
F6F Input UB

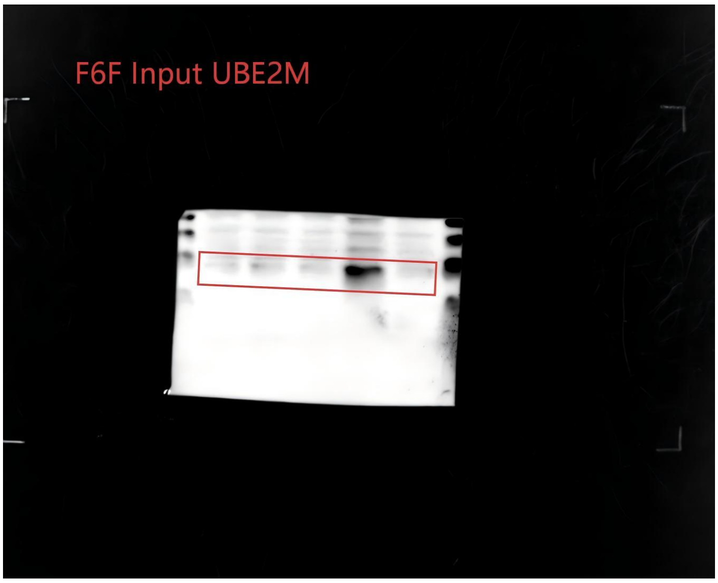
F6F Input UBE2M

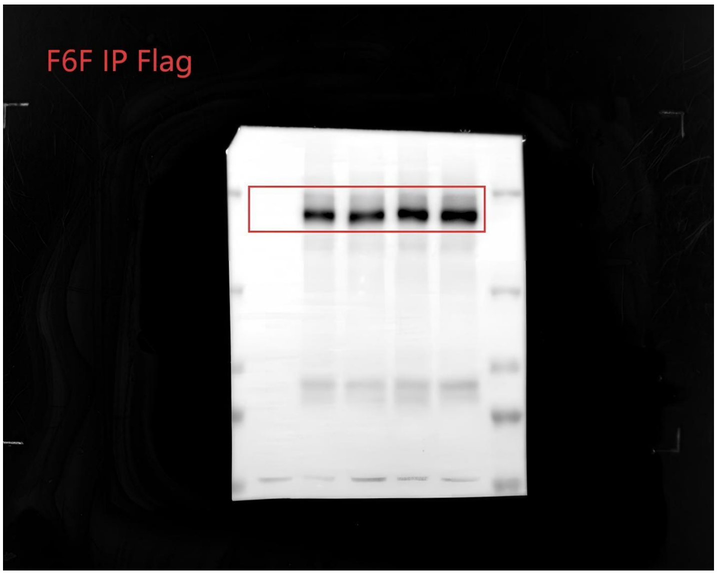
F6F IP Flag

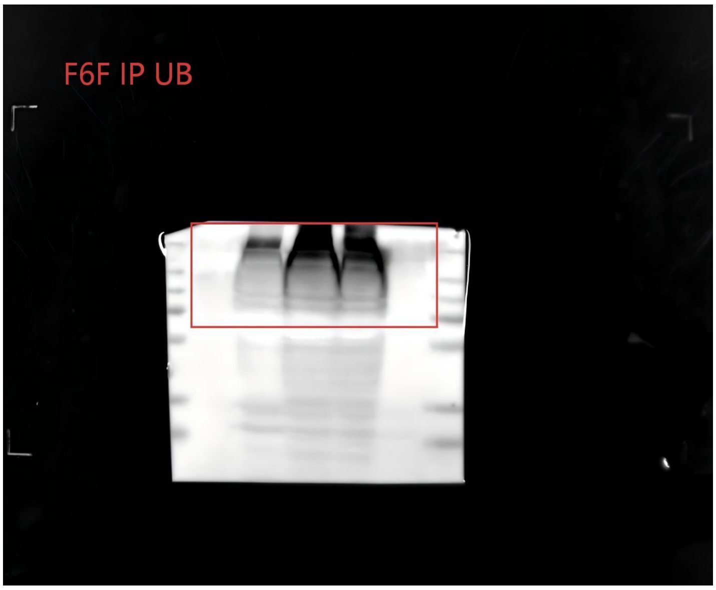
F6F IP UB

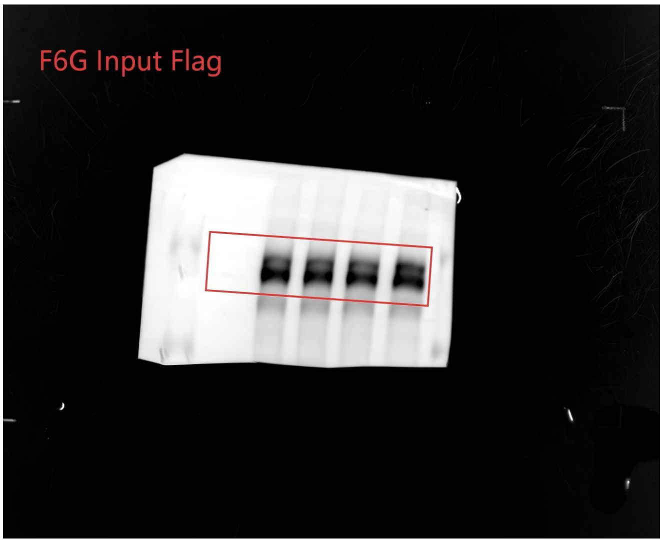
F6G Input Flag

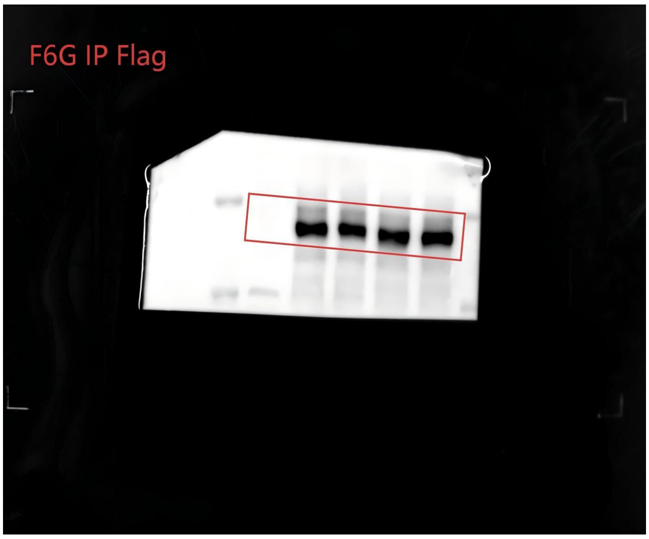
F6G IP Flag

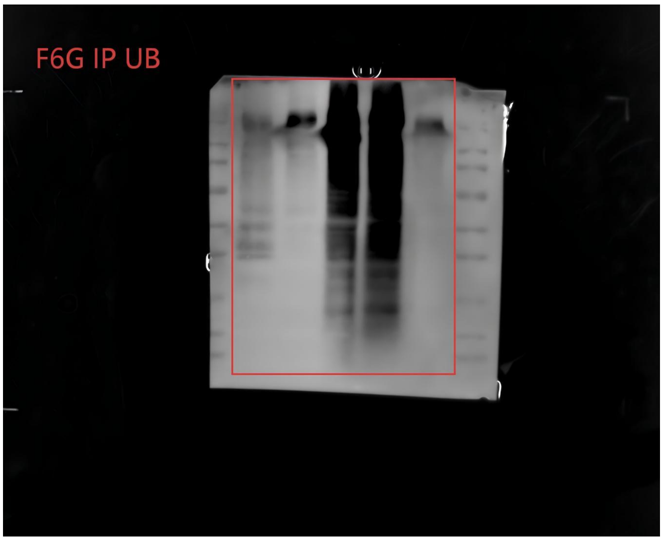
F6G IP UB

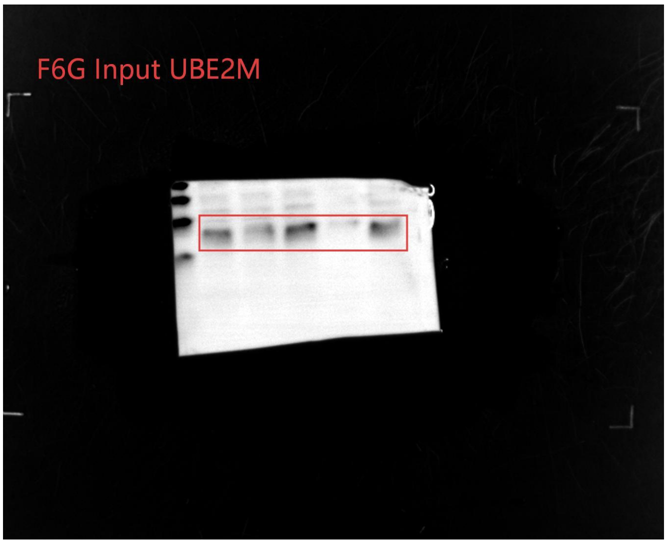
F6G Input UBE2M

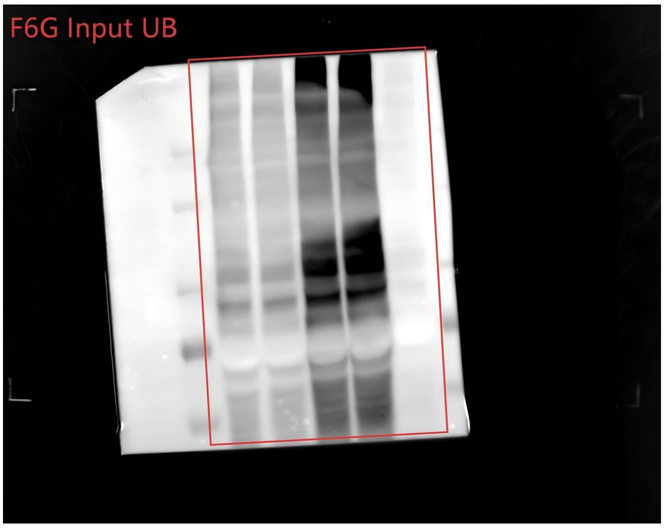
F6G Input UB

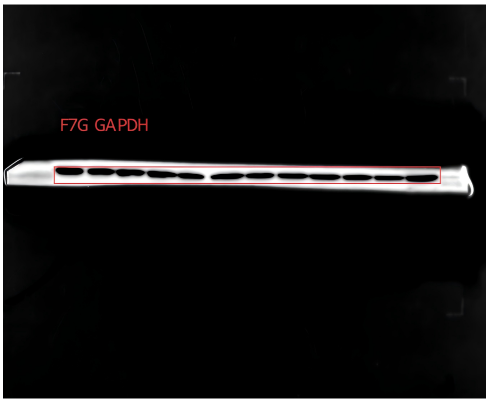
F7G GAPDH
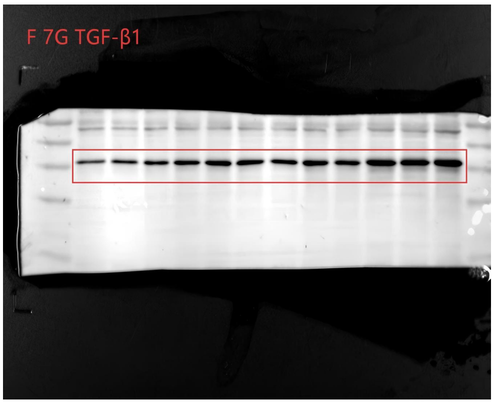
F 7G TGF-β1
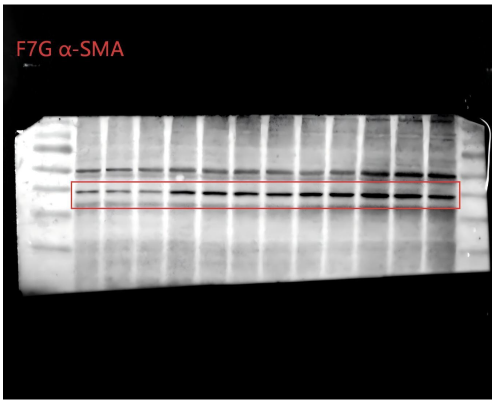
F7G α-SMA
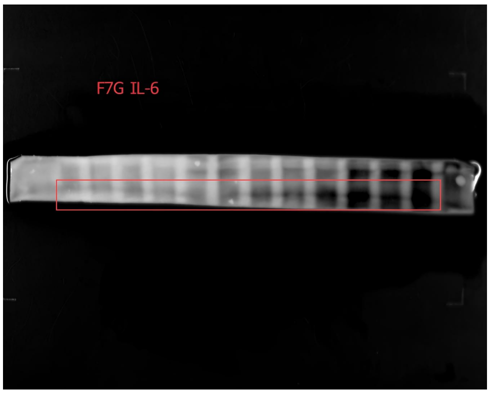
F7G IL-6
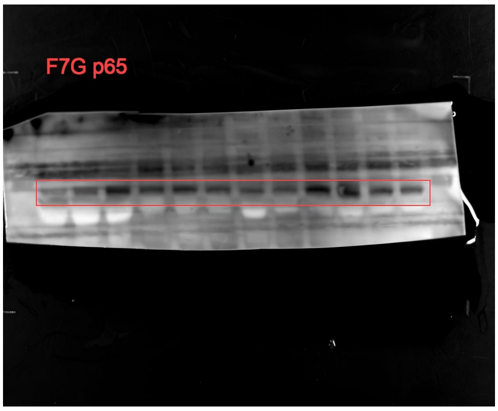
F7G p65
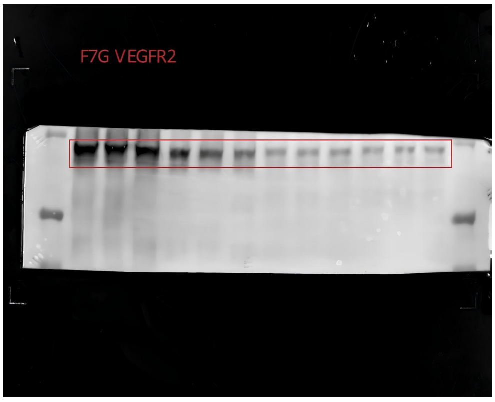
F7G VEGFR2
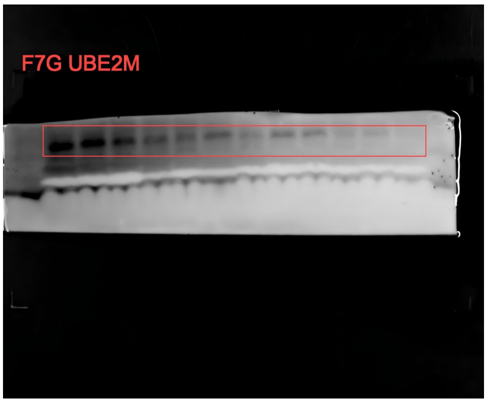
F7G UBE2M
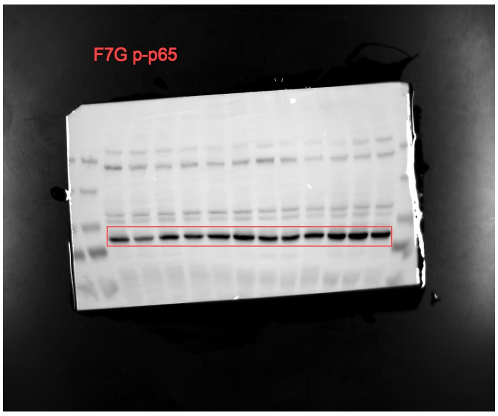
F7G p-p65
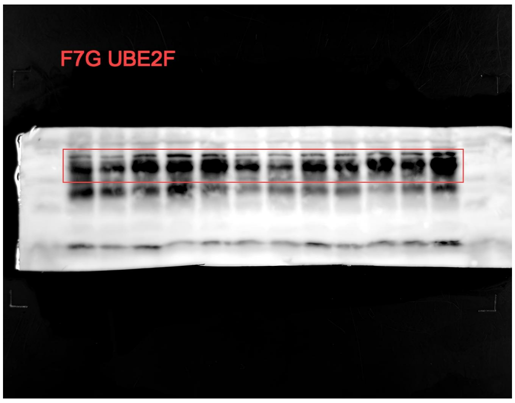
F7G UBE2F
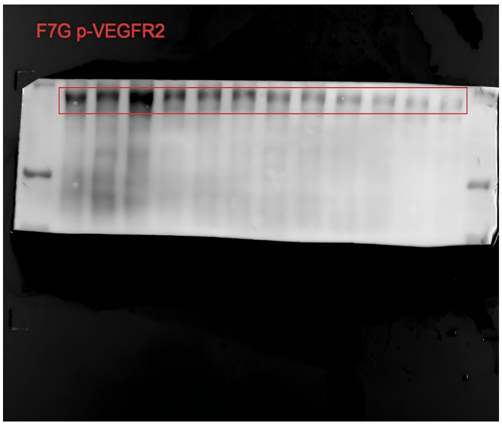
F7G p-VEGFR2

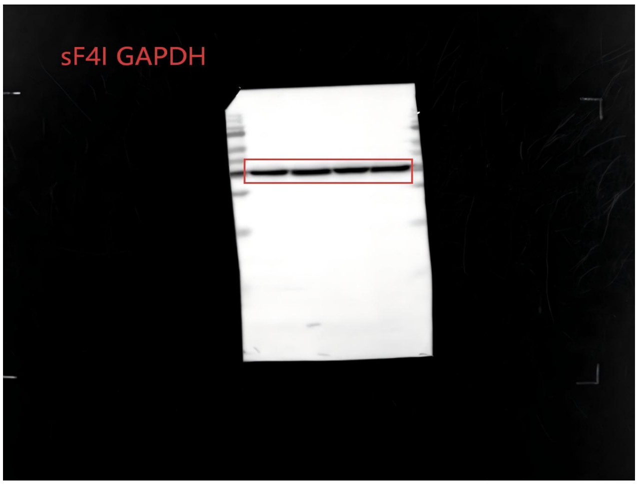
sF4I GAPDH

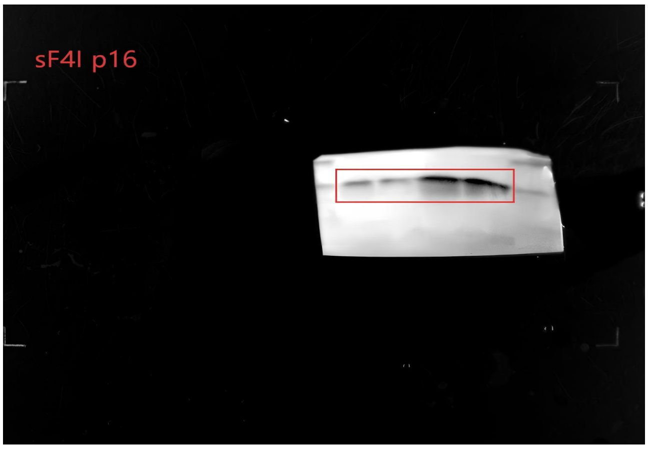
sF4I p16

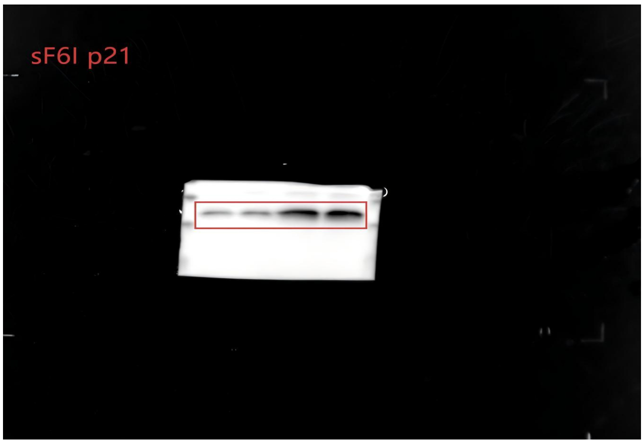
sF6I p21

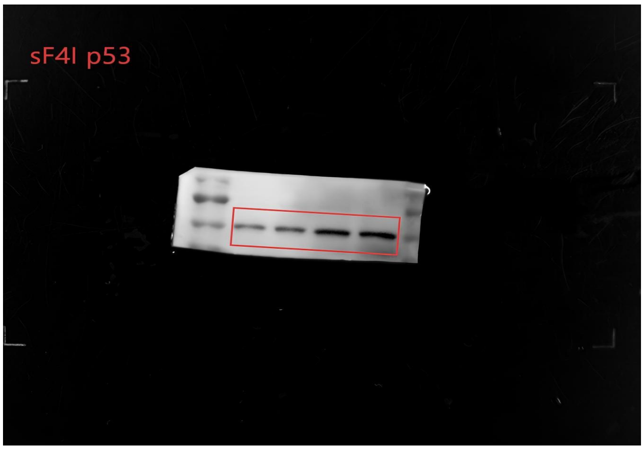
sF4I p53

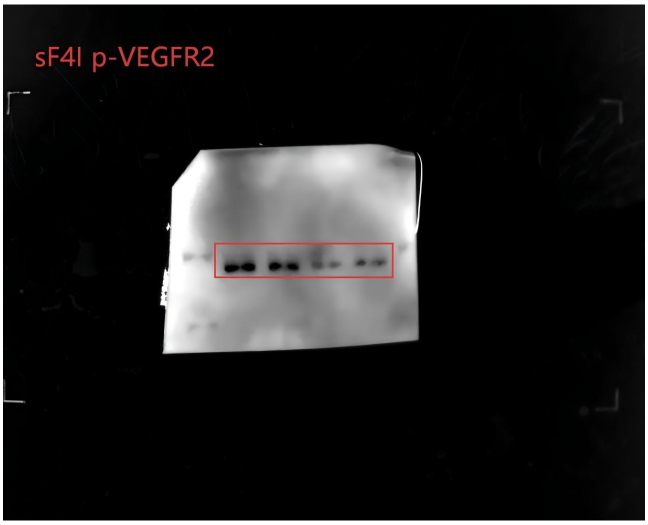
sF4I p-VEGFR2

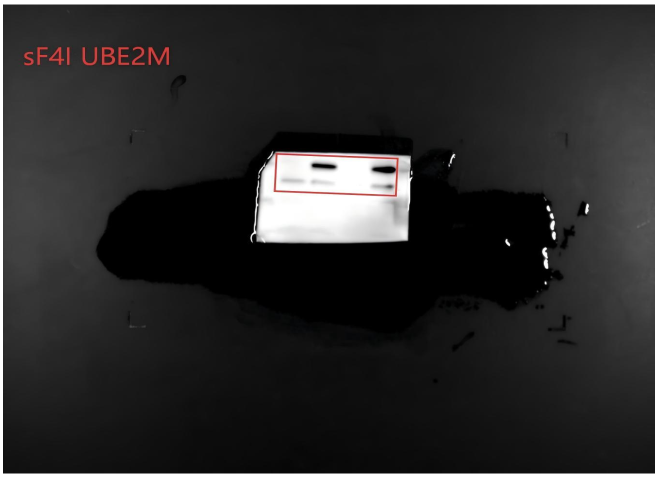
sF4I UBE2M

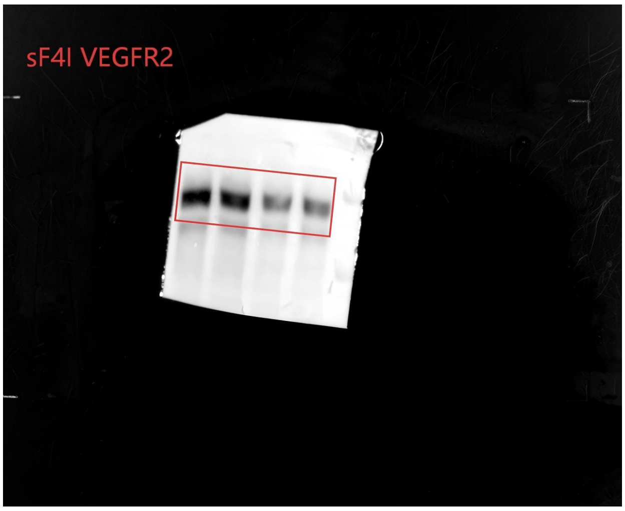
sF4I VEGFR2
